# Anthraquinone-based covalent organic framework as a recyclable direct hydrogen atom transfer photocatalyst for C–H functionalization†

**DOI:** 10.1039/d4sc00241e

**Published:** 2024-02-28

**Authors:** Zitong Wang, Pierce Yeary, Yingjie Fan, Wenbin Lin

**Affiliations:** a Department of Chemistry, The University of Chicago Chicago IL 60637 USA wenbinlin@uchicago.edu

## Abstract

Photocatalytic direct hydrogen atom transfer (d-HAT) is a synthetically important strategy to convert C–H bonds to useful C–X bonds. Herein we report the synthesis of an anthraquinone-based two-dimensional covalent organic framework, DAAQ-COF, as a recyclable d-HAT photocatalyst for C–H functionalization. Powder X-ray diffraction, N_2_ sorption isotherms, solid-state NMR spectra, infrared spectra, and thermogravimetric analysis characterized DAAQ-COF as a crystalline, porous COF with a stable ketoenamine linkage and strong absorption in the visible region. Under visible light irradiation, DAAQ-COF is photo-excited to cleave C(sp^3^)–H or C(sp^2^)–H bonds *via* HAT to generate reactive carbon radicals, which add to different radical acceptors to achieve C–N or C–C coupling reactions. DAAQ-COF is easily recovered from the reaction mixture *via* centrifugation or filtration and used in six consecutive reaction runs without any decrease in catalytic efficiency. The ease of catalyst separation allows sequential conversion of the C–N coupling intermediate to synthetically useful amide, ester, or thioester products. Photophysical and isotope labelling experiments support the d-HAT mechanism of DAAQ-COF-catalyzed C–H bond functionalization.

## Abstract 2

A COF-based catalyst was synthesized to activate C–H bonds for C–C/C–N coupling via hydrogen atom transfer. The catalyst can be easily recycled, allowing downstream modification of the product to realize C–H to C–N/C–S/C–O transformations.

## Graphical abstract

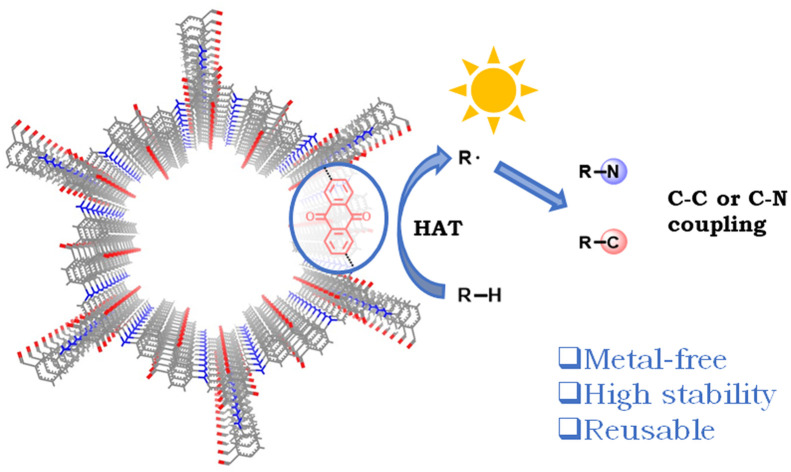

## Introduction

Selective functionalization of C–H bonds is a powerful synthetic method due to the universal presence of C–H bonds in organic compounds.^1–3^ C–H functionalization reactions are typically catalyzed by precious metals (Pd, Rh, Ir, *etc.*) at elevated temperatures owing to the low reactivity of C–H bonds.^4–8^ Photocatalyzed direct hydrogen atom transfer (d-HAT) presents an alternative strategy for C–H functionalization *via* homolytic cleavage of the C–H bond with the excited state of a photocatalyst.^9–11^ A hydrogen atom is abstracted from the substrate by the photoexcited catalyst to generate a reactive carbon radical, which adds to a radical acceptor and then undergoes radical rebound to form the desired C–H functionalized product. In such d-HAT processes, C–H bonds can be functionalized into various C–X bonds (X = C, N, *etc.*) *via* judicious choices of coupling agents. The selectivity of HAT reactions is typically high as the hydrogen abstraction step is dictated by the strength of different C–H bonds.

Carbonyl compounds are an important class of photocatalysts for d-HAT.^12,13^ In the photoexcited state, the C=O group can abstract a hydrogen atom from a substrate to form a HO–C˙ intermediate and a substrate radical to initiate the d-HAT cycle. Carbonyl HAT catalysts are more environmentally friendly and sustainable than their inorganic counterparts, such as decatungstate anion ([W_10_O_32_]^4−^), uranyl cation ([UO_2_]^2+^), or porphyrin–antimony oxo complexes, due to their metal-free nature.^14–16^ However, carbonyl HAT catalysts can be deactivated under photocatalytic conditions *via* multimolecular pathways (such as dimerization),^17,18^ and cannot be easily separated from reaction mixtures for reuse in additional catalytic cycles.

Covalent organic frameworks (COFs) are an emerging class of porous materials with an ordered arrangement of organic building blocks. The organic building blocks in COFs are connected with each other through covalent linkages (imine, akene, boronate ester, and others) to endow strong stability and incorporate interesting functions.^19–26^ Unlike traditional crosslinked organic polymers, ordered structures of crystalline COFs can be readily characterized by X-ray and electron diffraction to guide the rational design of functional COFs.^27,28^ For example, catalytic COFs have been designed using catalytically active units as the building units.^29–51^ The high porosity of COFs allows facile access of catalytically active sites to substrates and diffusion of products through COF channels. The use of appropriate linkages for COF construction has allowed the synthesis of stable COF catalysts.^52–55^ Heterogeneous COF-based catalysts can be easily recovered after the reactions and reused in subsequent reaction cycles. These advantages of COFs have motivated us to explore the design of COF-based catalysts for C–N and C–C coupling reactions *via* d-HAT.

Here we report the first example of d-HAT catalyzed by a two-dimensional (2D) COF, DAAQ-COF, based on 2,6-diaminoanthraquinone (DAAQ) building units. Under visible light irradiation, DAAQ-COF activates C(sp^3^)–H and C(sp^2^)–H bonds to generate carbon-based radicals for C–C or C–N coupling reactions. Unlike indirect HAT, no additional oxidant is required in DAAQ-COF-catalyzed coupling reactions. The DAAQ-COF catalyst is easily separated from the reaction mixture, allowing convenient downstream modifications of the product to achieve C–H to C–N/C–S/C–O transformations. The ketoenamine linkage in DAAQ-COF endows high stability under photocatalytic conditions to allow for its recovery and use in six reaction cycles.

## Results and discussion

### Synthesis and characterization of DAAQ-COF

**Fig. 1:**
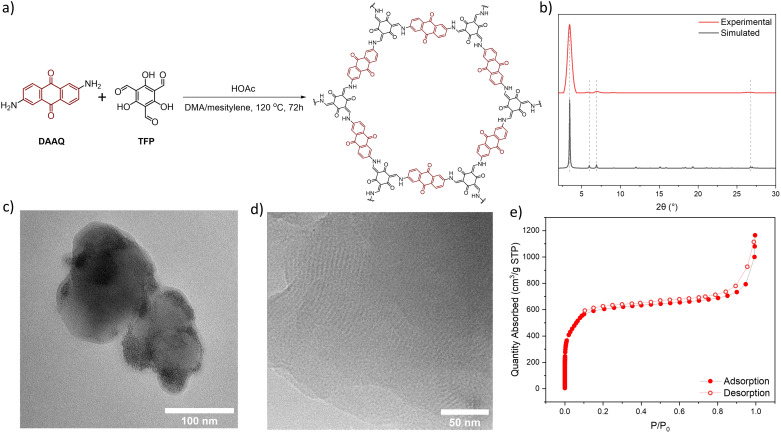
(a) Synthetic scheme of DAAQ-COF. (b) Experimental (red) and simulated (black) powder X-ray diffraction patterns of DAAQ-COF. (c) TEM image of DAAQ-COF. (d) High-resolution TEM image showing lattice fringes of DAAQ-COF. (e) N_2_ sorption isotherms of DAAQ-COF at 77 K.

DAAQ-COF was synthesized solvothermally by heating a mixture of DAAQ and 1,3,5-triformylphloroglucinol (TFP) at 120 °C in dimethylacetamide (DMA) and mesitylene with acetic acid (HOAc) as a modulator.^56,57^DAAQ-COF was collected *via* centrifugation and washed with DMA, and then stirred in DMA at 80 °C for 20 hours to completely remove residual DAAQ and TFP monomers. DAAQ-COF appeared dark red with an absorption maximum around 450 nm, suggesting its potential as a photocatalyst under visible light. DAAQ-COF exhibited high crystallinity with a good agreement between the experimental and simulated powder X-ray diffraction patterns (Fig. 1b). Rietveld fitting afforded a 2D network structure of DAAQ-COF with an interlayer distance of 3.3 Å. The PXRD pattern of DAAQ-COF did not match the simulated pattern based on the staggered (AB) stacking of 2D networks (Fig. S2†). The AA stacking of 2D layers afforded more porous structures with large channels of 24 Å in dimension to facilitate substrate access to the catalytic sites. The high porosity of DAAQ-COF was characterized by N_2_ sorption isotherms, affording a Brunauer–Emmett–Teller surface area of 1999 ± 55 m^2^ g^−1^ (Fig. 1e).

Transmission electron microscopy (TEM) imaging showed that DAAQ-COF possessed a spherical morphology with a diameter of ∼100 nm and exhibited lattice fringes of ∼3 nm in spacing (Fig. 1c and d), which was consistent with the lattice parameter of DAAQ-COF. The lattice fringes correspond to the in-plane periodicity of the 2D COF. Solid-state NMR spectrum of DAAQ-COF supported the complete imine condensation of DAAQ and TFP monomers and tautomerization of the TFP units (Fig. S8†) with C=O peaks at *δ* = 183 and 180 ppm for the DAAQ and tautomerized TFP moieties, respectively. The C=C peaks for the tautomerized TFP moieties appeared at *δ* = 142 and 108 ppm. The comparison of FT-IR spectra of the two monomers and DAAQ-COF revealed the disappearance of *ν*(C–H)_aldehyde_ at 2899 cm^−1^ due to the formation of enamine bonds (Fig. S7†). This β-ketoenamine-based linkage is more stable than the imine linkage in many COFs, which is important for catalytic applications.^58,59^ Thermogravimetric analysis was conducted by ramping the temperature from 20 °C to 800 °C at a 1.5 °C min^−1^ in air, and the result showed that DAAQ-COF was stable up to 350 °C and lost 100% of its weight by 500 °C (Fig. S9†).

### Catalytic performance of DAAQ-COF

**Fig. 2:**
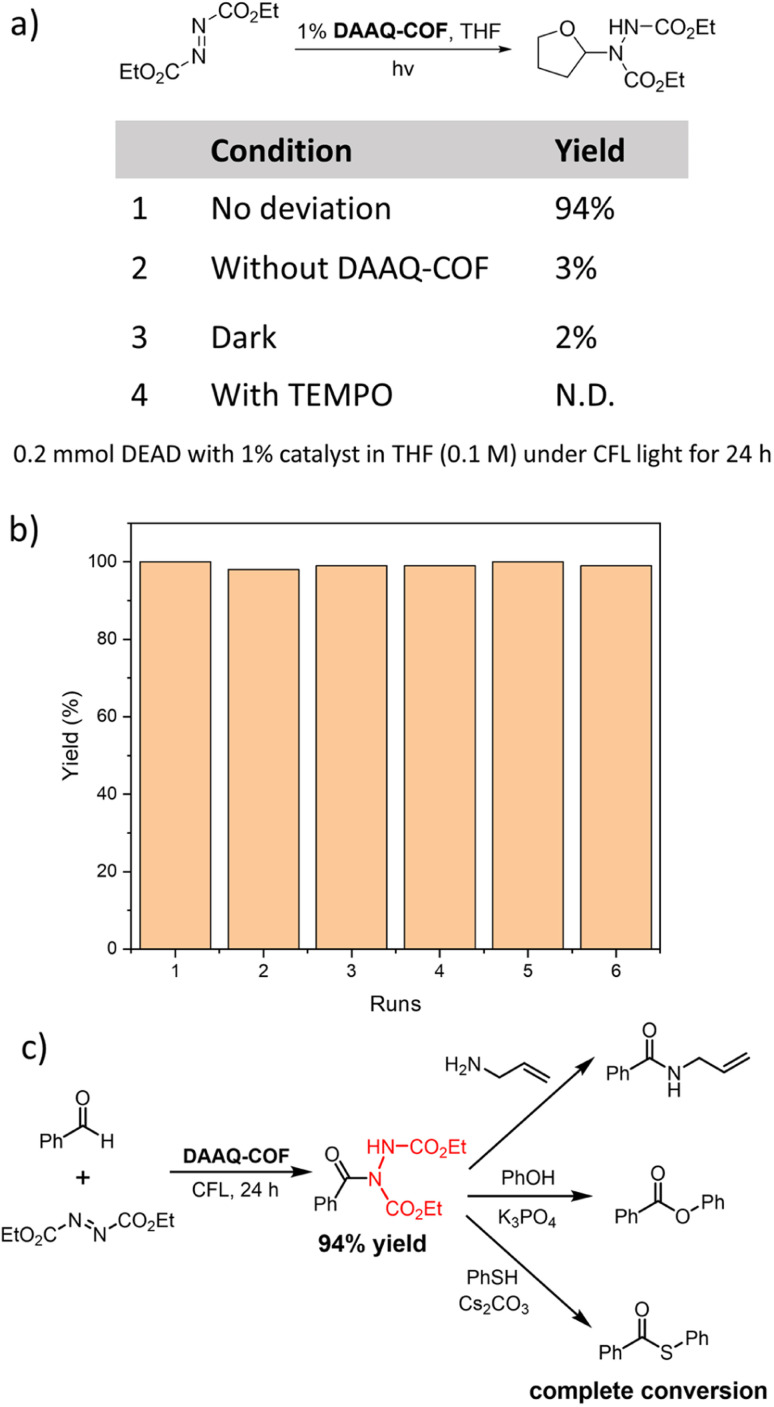
(a) Control experiments of DAAQ-COF catalyzed C–N coupling between THF and DEAD. (b) Yields of the C–N coupling product between THF and DEAD in six consecutive reaction runs with the recovered DAAQ-COF as catalyst. (c) A sequential approach to convert the C–H bond in benzaldehyde to C–O, C–S and C–N bonds without rigorous isolation of the intermediates.

As anthraquinone (AQ) was reported as a d-HAT catalyst, we tested the HAT catalytic performance of DAAQ-COF. The formation of C–N bonds is an important organic transformation in the synthesis of pharmaceutical compounds.^60^ We selected the coupling between diethyl azodicarboxylate (DEAD) and tetrahydrofuran (THF) as the model reaction to test the ability of the photoexcited DAAQ-COF in homolytically breaking the C–H bond and abstracting a hydrogen atom from THF. With 1% loading of DAAQ-COF catalyst, the yellow color of DEAD solution faded in 24 hours under CFL light irradiation, and the C–N coupling product was obtained in 94% yield. Without DAAQ-COF, there was only 3% conversion of DEAD, excluding the possibility that DEAD could spontaneously react with THF under compact florescent light (CFL) irradiation. A control experiment without light gave only 2% yield of the coupling product, with most of the starting material remaining unreacted. The addition of (2,2,6,6-tetramethylpiperidin-1-yl)oxyl (TEMPO) as a radical scavenger completely shut down the reaction without any conversion of DEAD, indicating the involvement of radical species in this d-HAT reaction. After the photocatalytic coupling reaction between DEAD and THF, DAAQ-COF was collected by centrifugation and washed with THF to remove trapped organic compounds. The recycled DAAQ-COF was used as the catalyst in five subsequent runs without any decrease of catalytic efficiency (Fig. 2b). The PXRD pattern of the recovered DAAQ-COF after six reaction runs demonstrated the retention of its crystallinity after d-HAT reactions. In contrast to homogeneous catalysts which are typically difficult to recover from reaction mixtures, DAAQ-COF was stable under photocatalytic conditions and retained its crystallinity after the reactions (Fig. S13†), allowing straightforward recovery from the reaction mixtures for reuse in subsequent reaction runs. Thus, DAAQ-COF shows great advantages over homogeneous d-HAT catalysts.

We also examined the substrate scope of DAAQ-COF-catalyzed C–N coupling reactions (Table 1). Both C(sp^3^)–H bonds and C(sp^2^)–H bonds were efficiently activated by DAAQ-COF*via* HAT to undergo C–N coupling with DEAD. Several activated C(sp^3^)–H bonds in THF (1a), dioxolane (1b), indane (1c), isochroman (1d) and various aldehydes (1e–1n) underwent d-HAT to afford C–N couple products in moderate to high yields. DAAQ-COF also showed high tolerance to different electron-withdrawing and electron-donating functional groups on aryl aldehyde substrates and exhibited great catalytic performance on aliphatic aldehydes as well.

**Table 1:** DAAQ-COF-catalyzed C–N and C–C coupling reactions with DEAD^a^ and the activated pyridine^b^

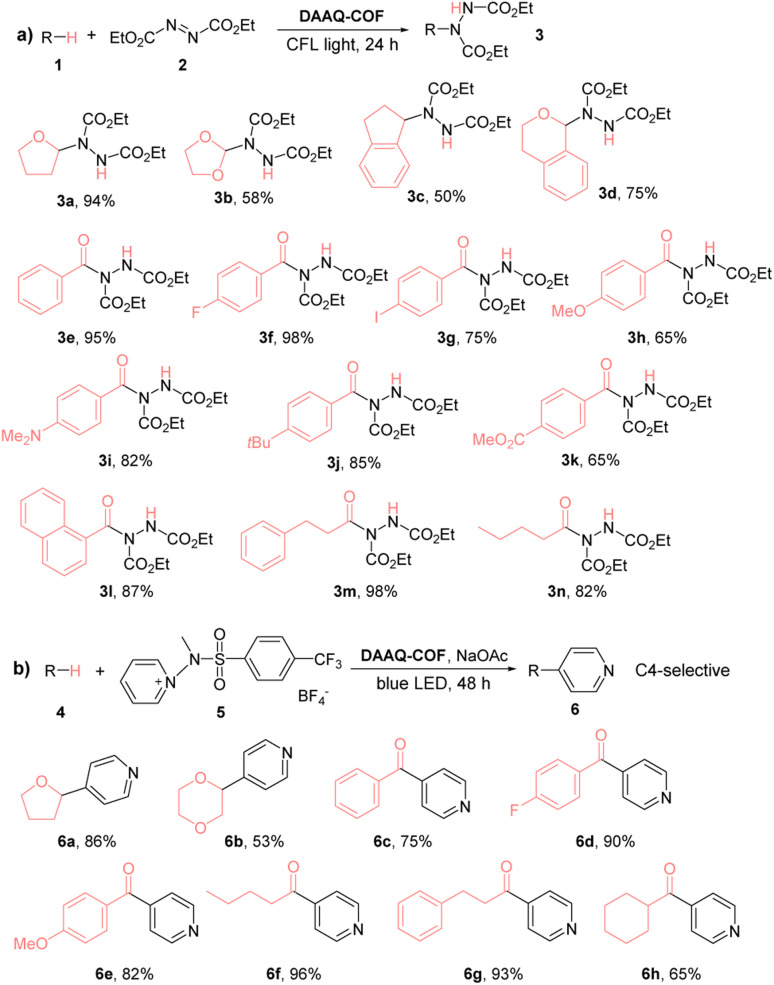

a Reactions were performed at 0.2 mmol scale with 1 mol% DAAQ-COF for 24 h in the corresponding solvents (THF for 3a, dioxolane for 3b, acetone for 3c–n).

b Reactions were performed at 0.2 mmol scale with 2 mol% loading of DAAQ-COF for 48 h in the corresponding solvents (THF for 6a, 1 : 1 dioxane/acetonitrile for 6b, acetonitrile for 6c–h). Isolated yields for all reactions.

As DAAQ-COF can be easily removed from reaction mixtures by centrifugation or filtration, we tested the feasibility of sequential synthesis *via* direct conversion of the generated hydrazine imide to other products.^61,62^ After the C–N coupling between benzaldehyde and DEAD under photocatalytic condition, DAAQ-COF was removed from the reaction mixture by filtration, followed by the addition of nucleophilic reactants to the hydrazine imide intermediate. Through reactions with allylamine, phenol, and thiophenol, the hydrazine imide was quantitatively converted to the synthetically useful amide, ester, or thioester, respectively (Fig. 2c). This sequential approach elaborated unfunctionalized C–H bonds to useful C–X bonds under a photocatalytic condition with a recyclable metal-free catalyst.

We next examined the HAT efficiency of DAAQ-COF in C–C coupling reactions. After transferring a hydrogen atom from C(sp^3^)–H bonds and C(sp^2^)–H bonds to the photoexcited DAAQ-COF catalyst, the generated carbon radicals reacted with activated pyridine to afford C–H pyridylation products with exclusive C4-selectivity (>98%).^63^ As shown in Table 1b, 2% loading of DAAQ-COF catalyzed C–C coupling between the activated pyridine and THF or dioxane to afford pyridyl furane 6a in 86% yield or pyridyl dioxane 6b in 53% yield, respectively. Aryl aldehydes with different substituents, including hydrogen, electron-withdrawing fluorine, and electron-donating methoxy group were tolerated in the reactions to give products 6c–6e in 75–90% yields. Aliphatic aldehydes were also readily pyridylated to afford products 6f–6h in high yields.

### Mechanistic study

**Fig. 3:**
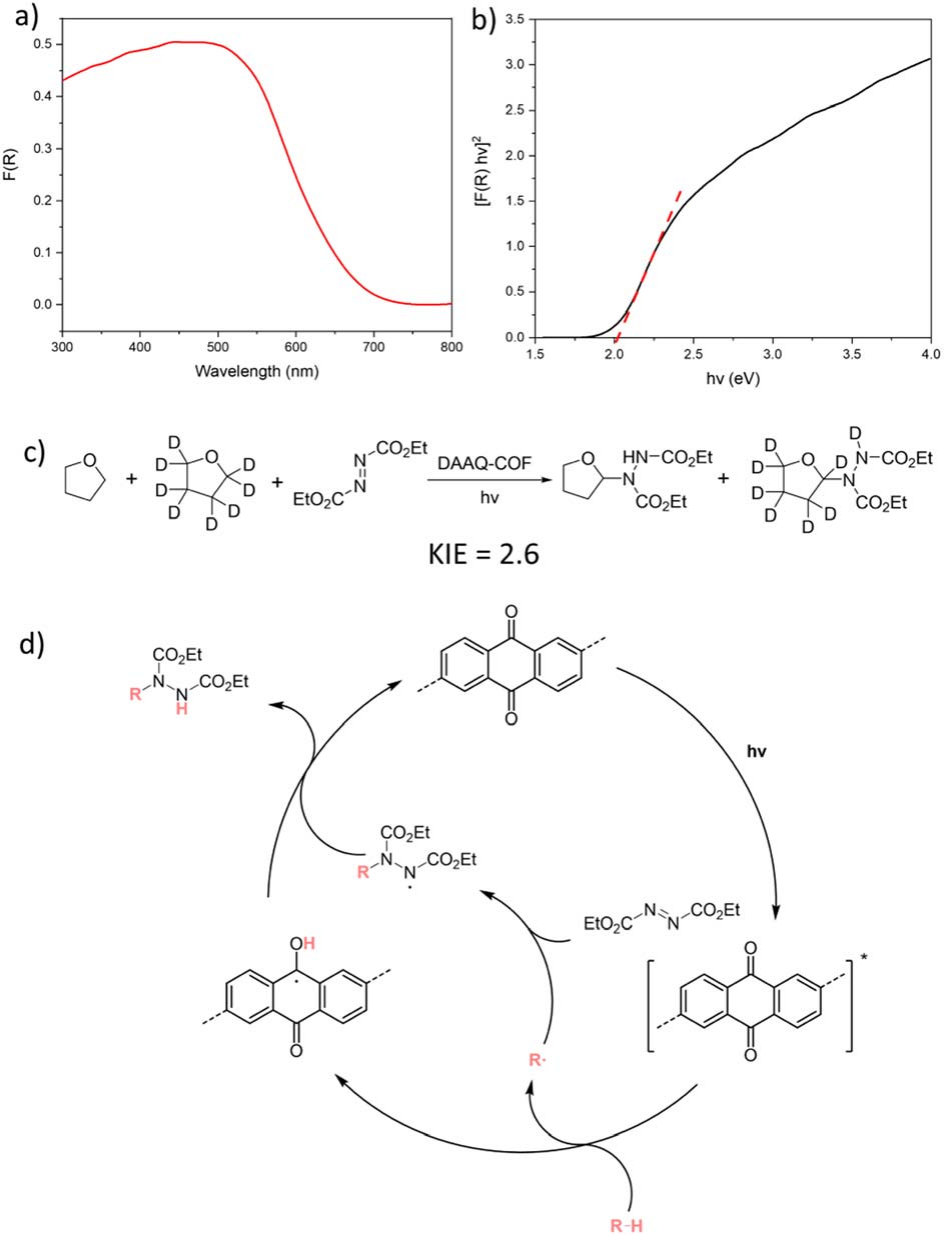
(a) Diffuse reflectance UV-vis spectrum of DAAQ-COF. (b) Tauc plot from the diffuse reflectance UV-vis spectrum of DAAQ-COF. (c) Kinetic isotope experiment of DAAQ-COF-catalyzed C–N coupling reaction between DEAD and a mixture of THF and *d*_8_-THF in a one-pot reaction. (d) Proposed mechanism for DAAQ-COF-catalyzed C–N coupling with DEAD as the radical acceptor.

Photophysical and kinetic isotope effect (KIE) experiments were conducted to elucidate the mechanism of DAAQ-COF-catalyzed d-HAT reactions. The diffuse reflectance UV-vis spectrum of DAAQ-COF exhibited a broad absorption in the visible light region with the maxima at 440 to 480 nm (Fig. 3a). The optical band gap of DAAQ-COF was estimated to be 2.02 eV with the Tauc plot (Fig. 3b). DAAQ-COF exhibited emissions at 453 nm and 545 nm with 400 nm excitation (Fig. S16†). These results show that DAAQ-COF can be irradiated by visible light to its excited state to initiate the catalytic cycle. No significant quenching was observed between the excited DAAQ-COF and benzaldehyde, suggesting no rapid electron transfer or energy transfer between the photoexcited catalyst and the substrate (Fig. S17†). The KIE experiment on DAAQ-COF-catalyzed C–N coupling reaction was conducted using DEAD and a 1 : 1 mixture of THF and *d*_8_-THF.^13,64^ The intermolecular KIE was determined to be 2.6 by calculating the product distribution from the NMR spectrum (Fig. 3c). As this value is a typical of primary KIEs, we believe that the cleavage of the C–H bond in the substrate is the rate-determining step in the d-HAT processes. Based on these findings, we propose a catalytic cycle for the photocatalyzed C–N coupling reaction in Fig. 3d. Under visible light irradiation, the excited AQ moiety in DAAQ-COF homolytically cleaves the C–H bond in the substrate *via* abstracting a hydrogen atom. The resulting substrate radical is quenched by the radical acceptor DEAD to generate a N-based radical, which abstracts a hydrogen atom to the H-AQ˙ species to form the C–N coupling product and regenerate the AQ catalyst. Similarly, in the C–C coupling reaction, the substrate radical can be quenched by 5 to realize pyridylation. The leaving amine radical undergoes reverse hydrogen atom transfer and converts the H-AQ˙ species back to the AQ catalyst (Fig. S20†). Light on/off experiment ruled out the involvement of a radical chain process in the C–C coupling reaction, as no product was generated after the light was turned off (Fig. S19†).

## Conclusions

In this work, we synthesized an anthraquinone-based 2D COF as a d-HAT catalyst. Under visible light irradiation, DAAQ-COF cleaves the C–H bond of the substrate to generate a carbon radical, which further reacts with different radical acceptors such as DEAD or activated pyridine to realize C–N or C–C coupling reactions. As a heterogenous catalyst, DAAQ-COF is easily separated from the mixture by filtration or centrifugation and reused in five reaction runs without any decrease in catalytic performance. The C–N coupling product in the filtrate directly react with nucleophilic reagents to realize C–H bond functionalization. This work highlights the potential of designer COFs in catalytic C–H functionalization *via* d-HAT processes.

## Data availability

All the data supporting this article have been included in the main text and the ESI.†

## Author contributions

Z. Wang and W. Lin conceived the project and wrote the manuscript. Z. Wang and P. Yeary synthesized the material conducted catalytic experiments. Z. Wang and Y. Fan characterized the material.

## Conflicts of interest

There are no conflicts to declare.

## Supplementary Material

SC-015-D4SC00241E-s001
