# Supplementary material for: Anthraquinone-based covalent organic framework as a recyclable direct hydrogen atom transfer photocatalyst for C–H functionalization
Source: Chem Sci. 2024 Feb 28;15(13):4920–5. doi: 10.1039/d4sc00241e (PMC10966989; doi:10.1039/d4sc00241e)
Supplement: SC-015-D4SC00241E-s001 [file SC-015-D4SC00241E-s001.pdf]

Electronic Supplementary Material (ESI) for Chemical Science.
This journal is © The Royal Society of Chemistry 2024

Supporting Information

Anthraquinone-Based Covalent Organic Framework as a Recyclable Direct Hydrogen Atom Transfer Photocatalyst for C-H Functionalization

Zitong Wang, Pierce Yeary, Yingjie Fan, and Wenbin Lin*

Department of Chemistry, The University of Chicago, 929 East 57th Street, Chicago, IL, 60637, USA

Email: wenbinlin@uchicago.edu

Contents

1. Materials and Methods 2
2. Material Synthesis and Characterization 3
3. General Procedures for Catalytic Reactions and Control Experiments 15
4. Mechanistic Study 21
5. Product Characterization 26
6. References 36

1. Materials and Methods

All starting materials were purchased from Sigma-Aldrich and Fisher (USA) unless otherwise noted and used without further purification. Powder X-ray diffraction (PXRD) data were collected on SAXSLAB's GANESHA in the transmission mode. Transmission electron microscopy (TEM) was carried out on a TECNAI Spirit microscope. N_2 sorption measurements were carried out on a Micromeritics 3FLEX instrument at 77 K. Solid-state ^{13}C NMR data was acquired on a three channel 400 MHz Bruker Avance III HD system at Northwestern University. Thermogravimetric analysis (TGA) was performed in air using a Shimadzu TGA-50 equipped with a platinum pan and heated at a rate of 1.5 °C/min. Fourier-transform infrared (FT-IR) spectra were collected using a Thermo NEXUS 670 Near-, Far-, and Mid-FTIR with attenuated total reflectance (ATR) accessory (for powder samples). ^{1}H NMR, ^{19}F NMR and ^{13}C NMR spectra were recorded on a Bruker DRX at 400, 376, and 101 MHz, respectively, and referenced to the resonances resulting from the solvents. ^{1}H NMR spectra were reported as follows: chemical shift (δ ppm), multiplicity (s = singlet, d = doublet, t = triplet, m = multiplet, dd = doublet of doublets, br = broad peak), coupling constants (Hz), and integration. Emission spectra were recorded on a Shimadzu RF-5301PC spectrofluorophotometer. Diffuse reflectance UV-vis spectrum was collected on a CARY 5000 spectrophotometer. A powder sample was mixed with KBr as the non-absorbing matrix and loaded in a Praying Mantis diffuse reflectance cell. The Kubelka-Munk conversion of the raw diffuse reflectance spectrum was obtained by applying the formula $F(R) = (1-R)^2/2R$. Deuterated reagents and solvents used for kinetic isotope effect (KIE) experiments and NMR studies were purchased from Cambridge Isotope Laboratories, Inc. Mass spectrometric analyses were conducted using positive-ion electrospray ionization on a Bruker BioTOF Mass Spectrometer. Materials Studio was used to visualize COF structures and PXRD data processing and fitting.

2. Material Synthesis and Characterization

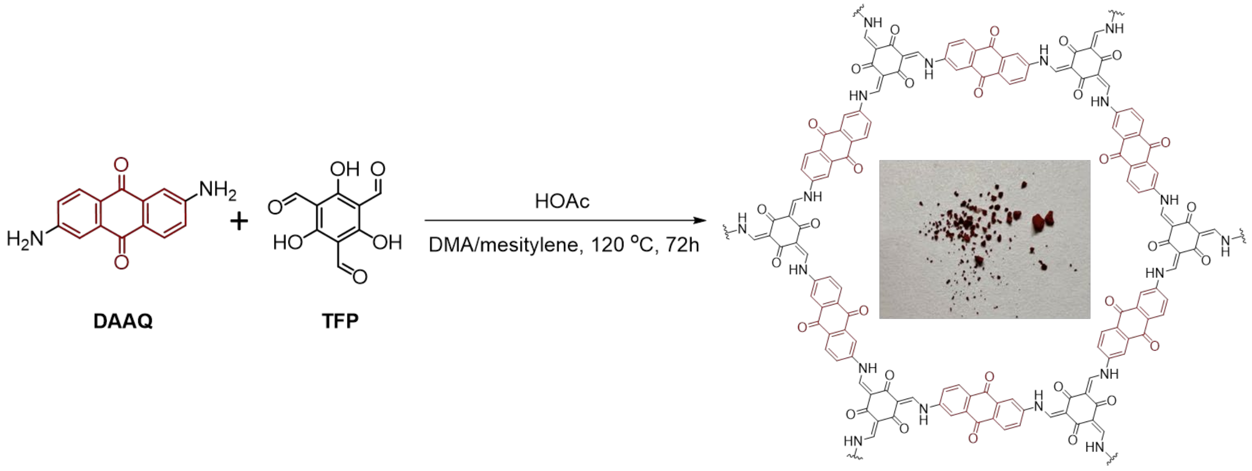

Synthesis of DAAQ-COF. DAAQ-COF was synthesized via a modified method based on the previous literatures.[1-2] A 10 mL Schlenk tube was charged with 2,6-diaminoanthraquinone (DAAQ, 104 mg, 0.44 mmol) and 1,3,5-triformylphloroglucinol (TFP, 60 mg, 0.29 mmol), dimethylacetamide (DMA, 2.6 mL) and mesitylene (1.0 mL). The mixture was briefly sonicated, and 0.37 mL of 6 M acetic acid (in water) was added as modulator. After another brief sonication, the mixture was degassed through three freeze-pump-thaw cycles using a liquid nitrogen bath. The tube was sealed under vacuum and heated at 120 °C for 3 days. After cooling to room temperature, the precipitate was collected via centrifugation and washed with 10 mL of DMA twice. The resultant powder was dispersed in 20 mL DMA and the mixture was stirred at 80 °C for 20 hours to remove any monomer trapped in the COF. After that, the COF powder was washed with 10 mL of acetone three times to remove DMA, and then dried under vacuum at room temperature. The product was isolated as dark-red solid. Yield: 103 mg (68%).

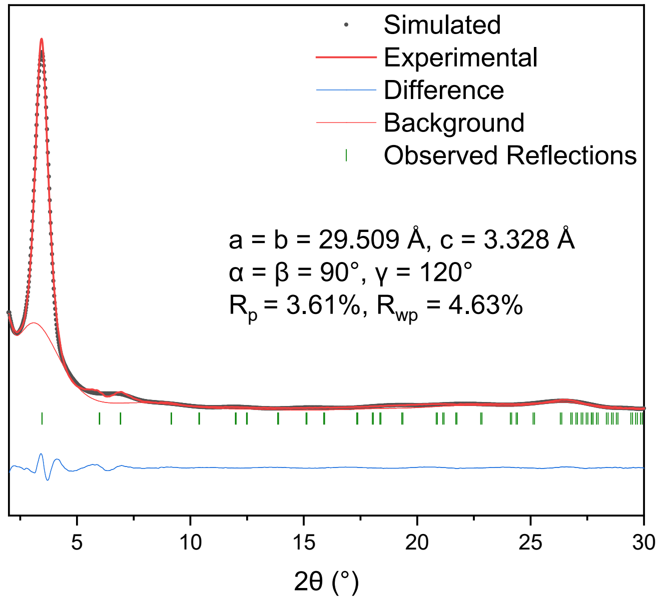

Figure S1. Simulated and experimental PXRD patterns of DAAQ-COF after Rietveld refinement. The detailed Rietveld refinement results for DAAQ-COF are shown in Table S1.

Table S1. Fractional atomic coordinates for the structural model of DAAQ-COF obtained from Rietveld refinements,

Space group: P6/M<br>a = b = 29.509 Å, c = 3.328 Å<br>α = β = 90°, γ = 120°			
Atom	x	y	z
C1	0.68157	0.38844	0.00000
C2	0.62626	0.34699	0.00000
C3	0.58639	0.35507	0.00000
N4	0.59334	0.40462	0.00000

C5	0.55265	0.41433	0.00000
C6	0.49938	0.37562	0.00000
C7	0.44474	0.46256	0.00000
C8	0.48107	0.44494	0.00000
C9	0.53494	0.48234	0.00000
C10	0.46317	0.39149	0.00000
C11	0.56935	0.46638	0.00000
O13	0.6956	0.43478	0.00000
H18	0.62881	0.43782	0.00000
O12	0.39651	0.43032	0.00000
H14	0.54587	0.32186	0.00000
H15	0.48337	0.33295	0.00000
H16	0.42075	0.36135	0.00000
H17	0.60965	0.49421	0.00000

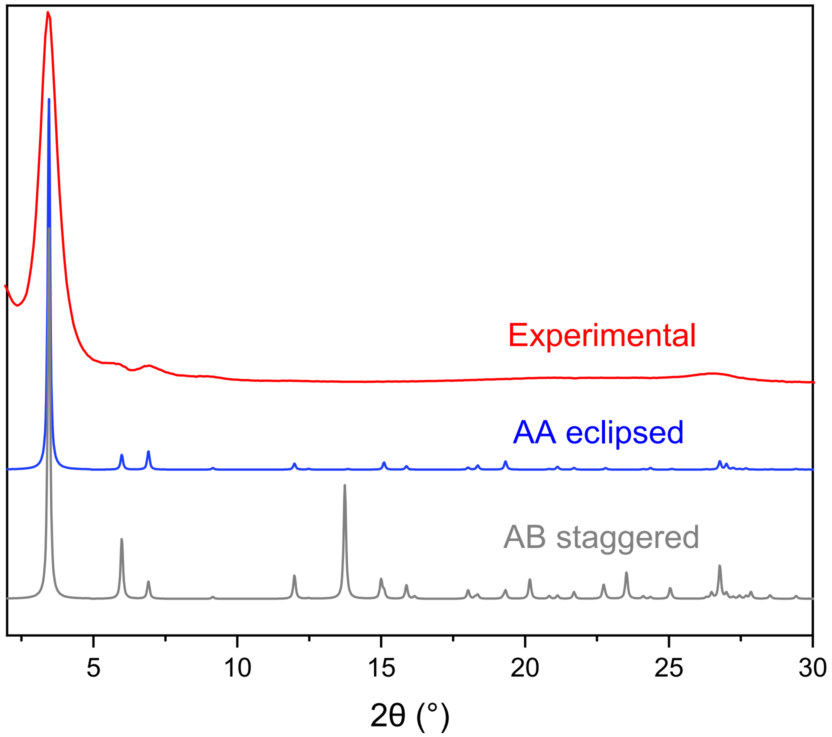

Figure S2. Experimental PXRD pattern of **DAAQ-COF** (red) in comparison with the simulated patterns based on AA stacking (blue) and AB stacking (grey) of 2D networks. The AA inclined stacking mode is also disfavored due to the energetic penalty from the decreased π-π interaction.

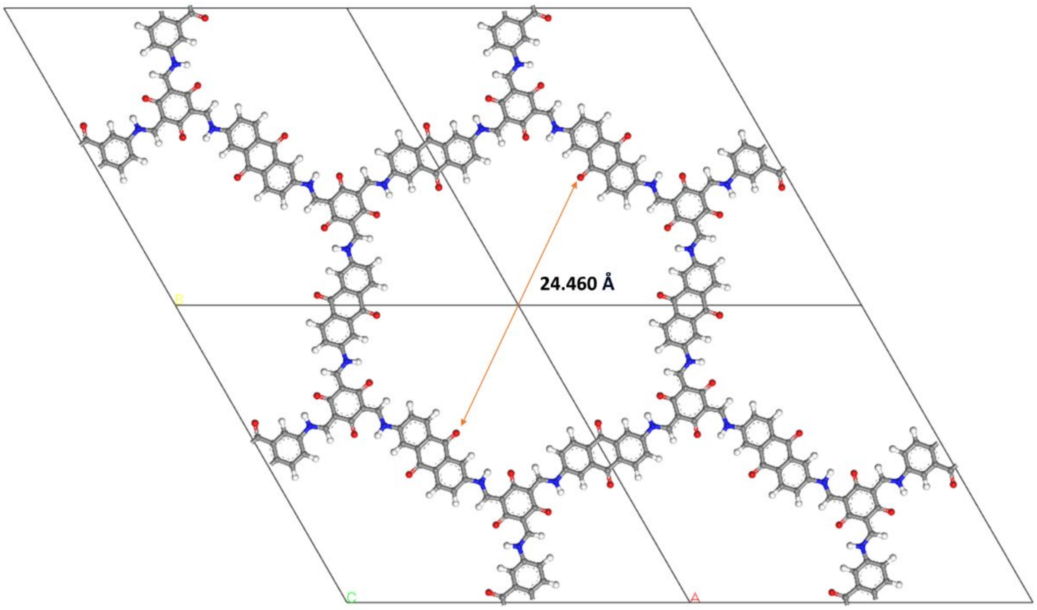

Figure S3. Structural model of DAAQ-COF from Rietveld refinement.

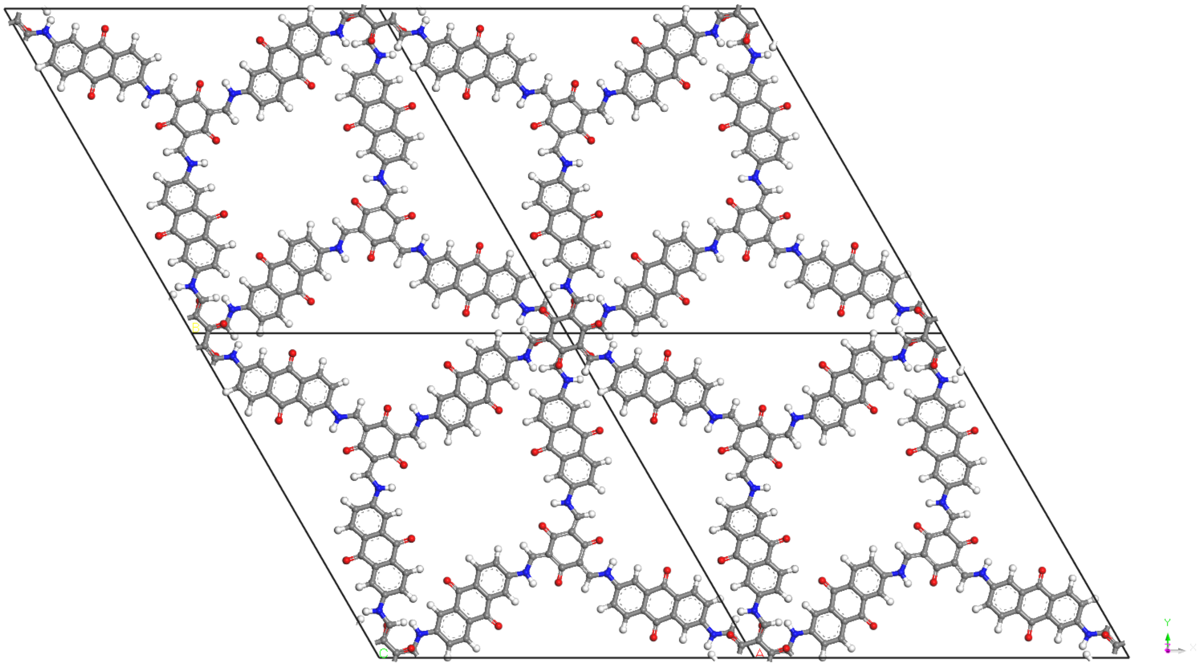

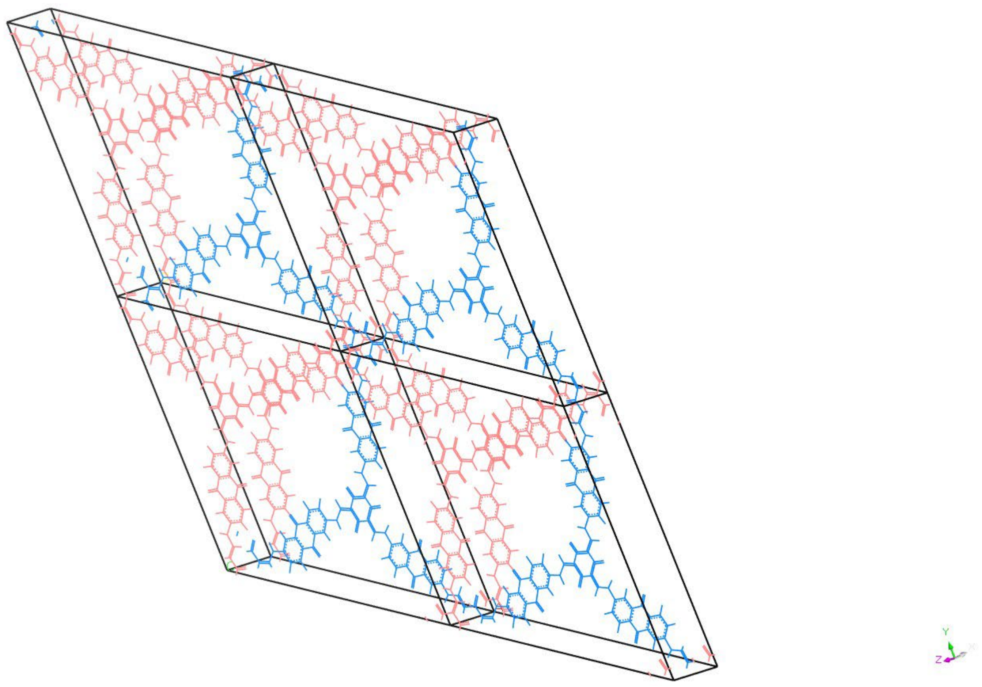

Figure S4. A proposed structural model of DAAQ-COF with AB stacking mode (top). The AB arrangement (red for A, blue for B) was also showed in a different view (bottom).

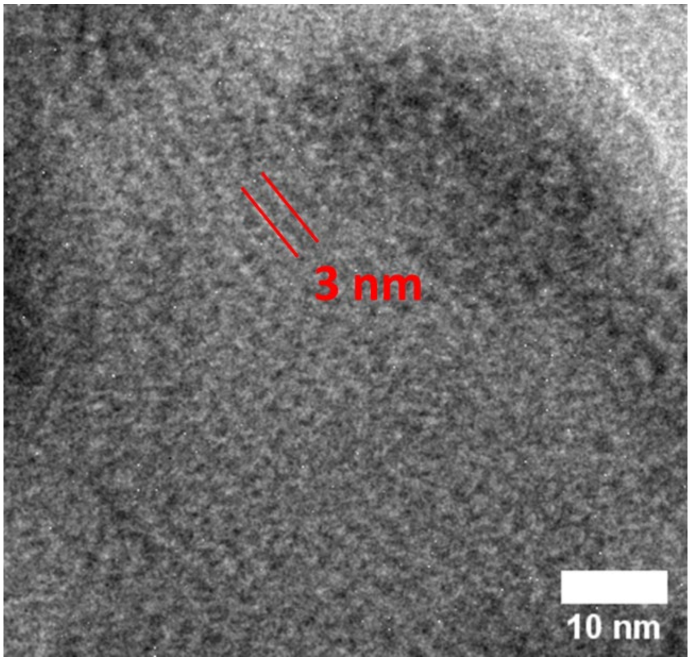

Figure S5. TEM image of **DAAQ-COF** at a 10 nm zoom scale to show the 3 nm distance between fringes.

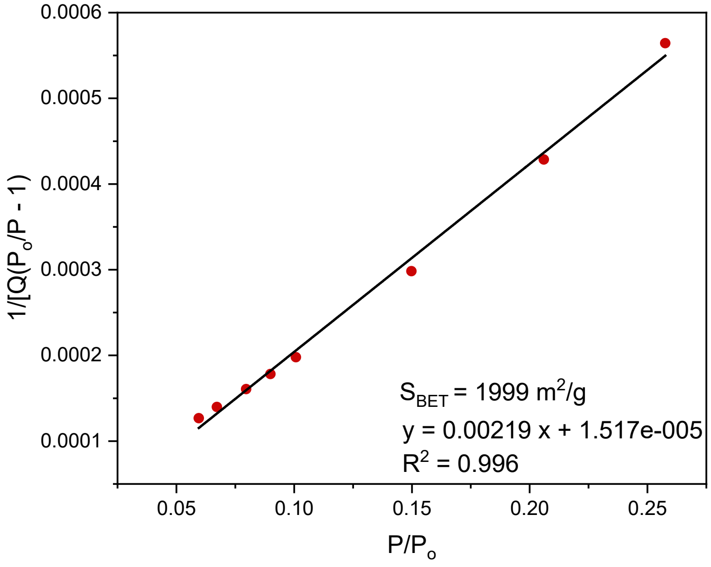

Figure S6. BET surface area plot of DAAQ-COF.

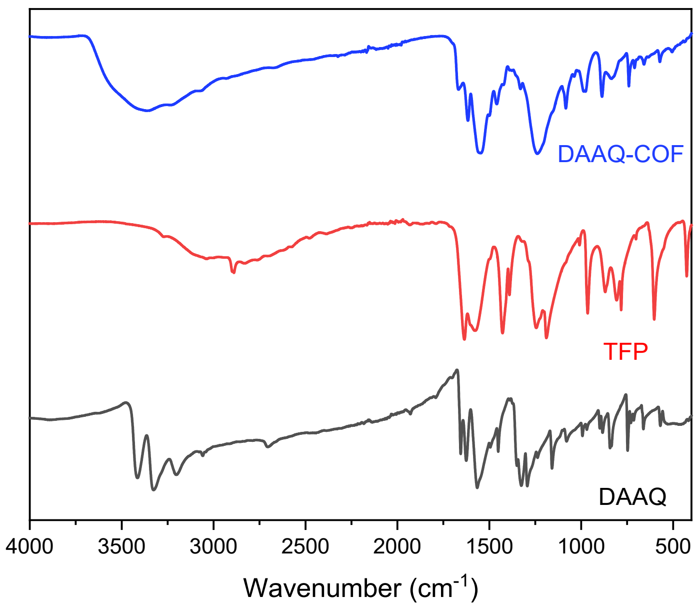

Figure S7. FT-IR spectra for DAAQ (black), TFP (red), and DAAQ-COF (blue). The disappearance of the characteristic doublet peak of formyl C-O stretching at ~2800 cm^{-1} supported imine condensation in COF synthesis.[1]

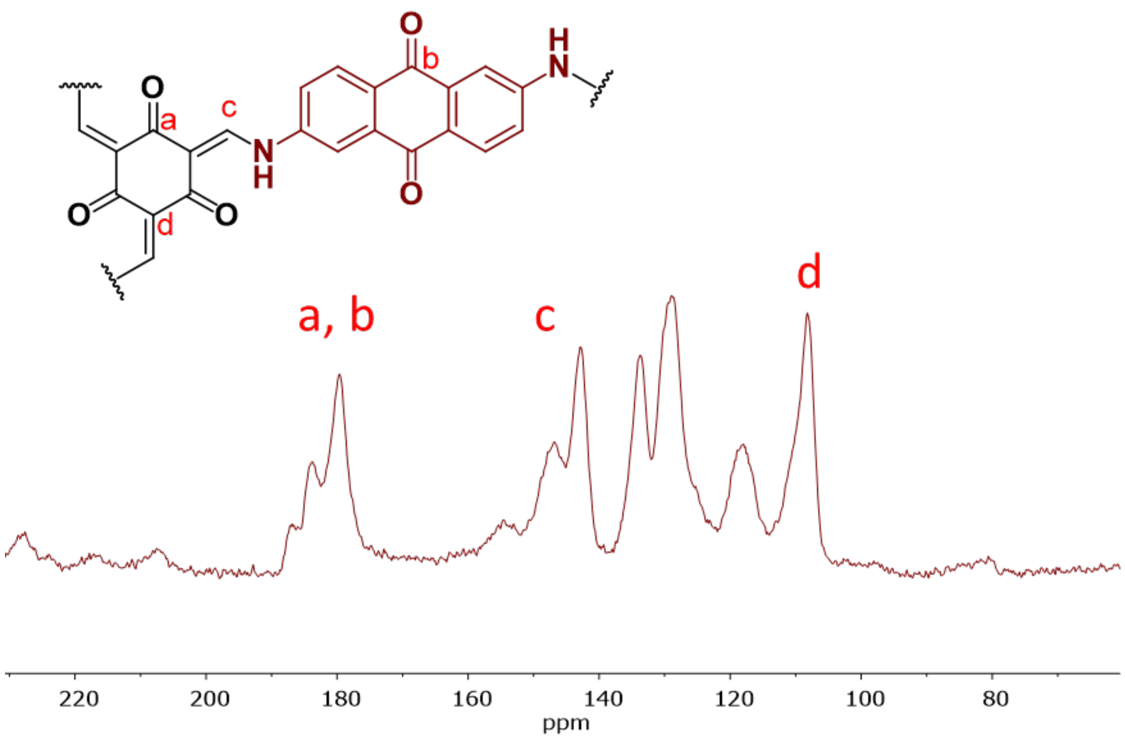

Figure S8. Solid-state ^{13}C NMR spectrum of DAAQ-COF. The result matches well with the previous report.[1]

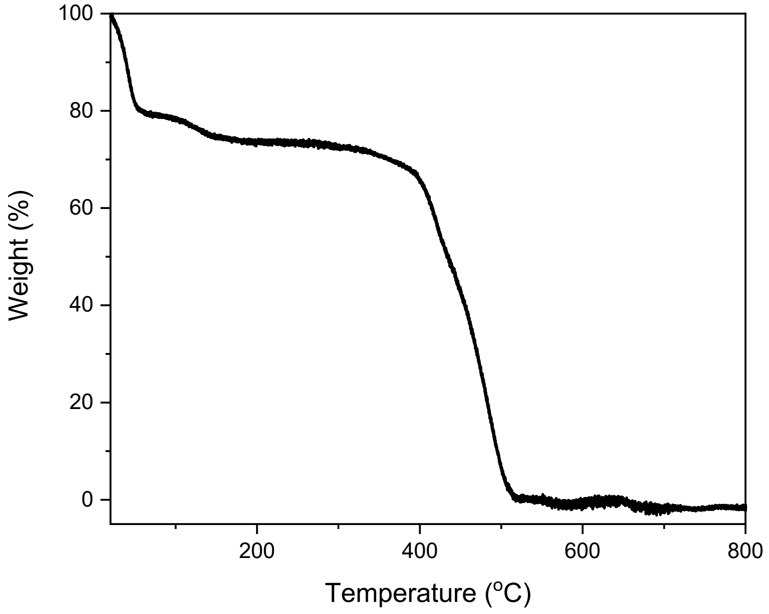

Figure S9. TGA analysis of DAAQ-COF. The result matches well with the previous report.[1]

Synthesis of the activated pyridine as substrate for DAAQ-COF-catalyzed C-C coupling.

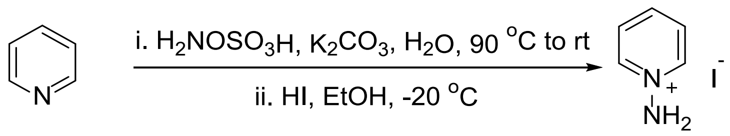

The N-aminopyridinium iodide was synthesized based on the previous literature.[3] 11.3 g (0.1 mol, 1 eq.) of hydroxylamine-O-sulfonic acid was dissolved in 64 mL of cold water. 24 mL (24 g, 0.3 mol, 3 eq.) of pyridine was added to the aqueous solution. The mixture then heated at 90 °C for 20 minutes, and then cooled to room temperature with stirring. 13.8 g (0.1 mol, 1 eq.) of K_2CO_3 was added to the reaction mixture. H_2O and excess pyridine were removed under vacuum and heating. 120 mL of ethanol was then added to the residue and the insoluble potassium salt was removed by filtration. Then, 14 mL (22 g, 0.1 mol, 1 eq.) of 57% hydroiodic acid was added to the filtrate. The solution was stored at -20 °C for 1 hour. The precipitated solid was collected by filtration and purified by recrystallization from 100 mL of ethanol. The N-aminopyridinium iodide product

appeared as pale-pink needle-like crystals. Yield: 1.3 g (58%). ^{1}H NMR (400 MHz, DMSO-d_6): δ 8.76 (m, 2H), 8.45 (s, 2H), 8.28 (m, 1H), 8.02 (m, 2H). The minor impurity is pyridinium salt which can be removed in the next step.

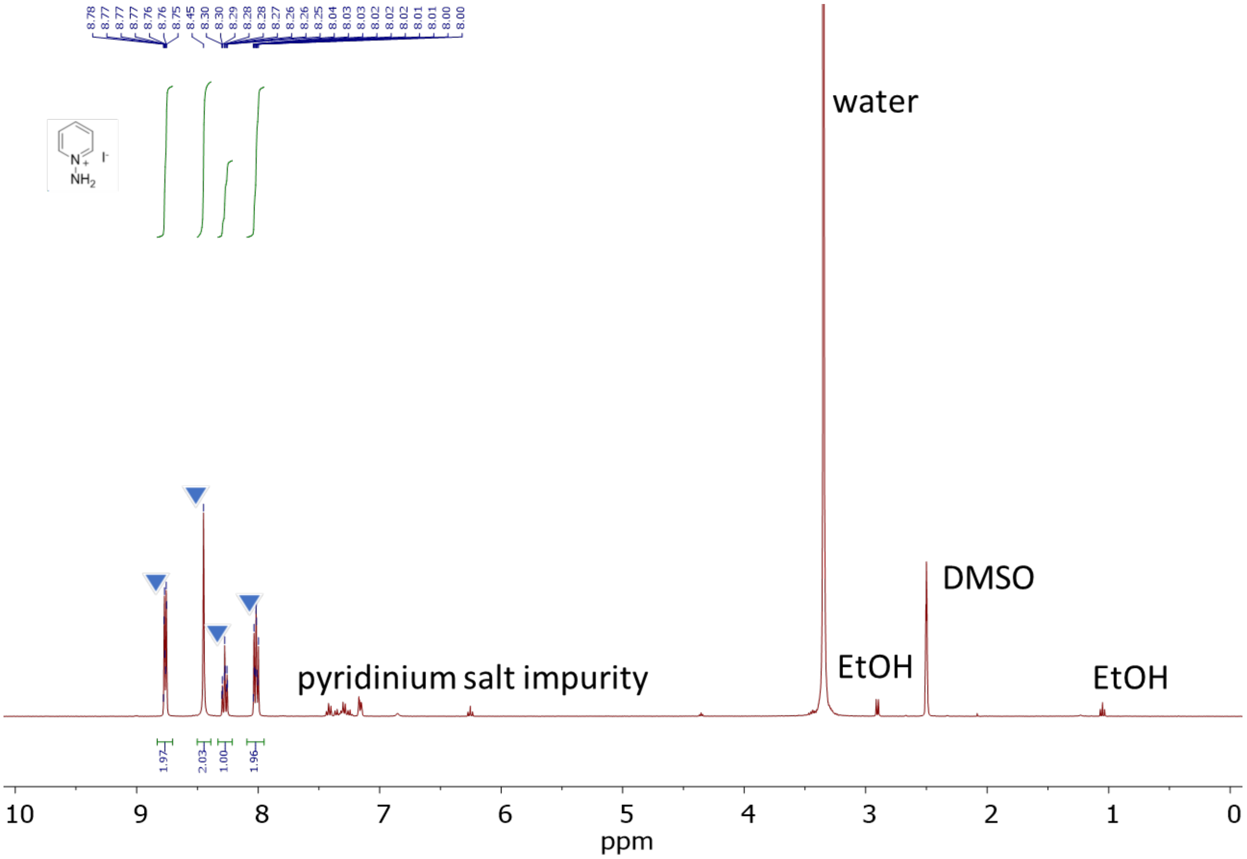

Figure S10. ^{1}H NMR of N-aminopyridinium iodide in DMSO-d_6 (400 MHz).

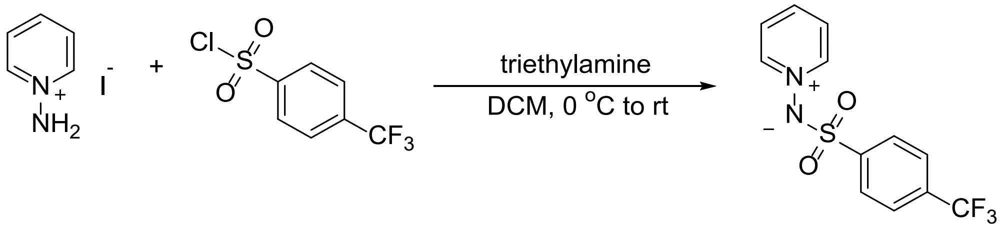

N-aminopyridinium iodide (2.22 g, 10 mmol, 1 eq.) was dispersed in 50 mL of dichloromethane. 3.07 mL of triethylamine (22 mmol, 2.2 eq.) and trifluoromethyl sulfonyl chloride (2.45 g, 10 mmol, 1 eq.) were added to the dispersion at 0 °C. The reaction mixture was stirred at room temperature for 24 hours. The reaction mixture was diluted with 1M NaOH aqueous solution and extracted with 30 mL of dichloromethane three times. The organic layer collected was dried over Na_2SO_4, filtered, and concentrated under vacuum. The crude product was purified by column chromatography using DCM and MeOH (20:1) as eluent. Yield: 2.69 g (89%). ^{1}H NMR (400 MHz, chloroform-*d*): δ 8.45 (d, J = 5.83 Hz, 2H), 8.04 (m, 1H), 8.28 (7.85, J = 8.14 Hz, 2H), 7.64 (m, 4H).

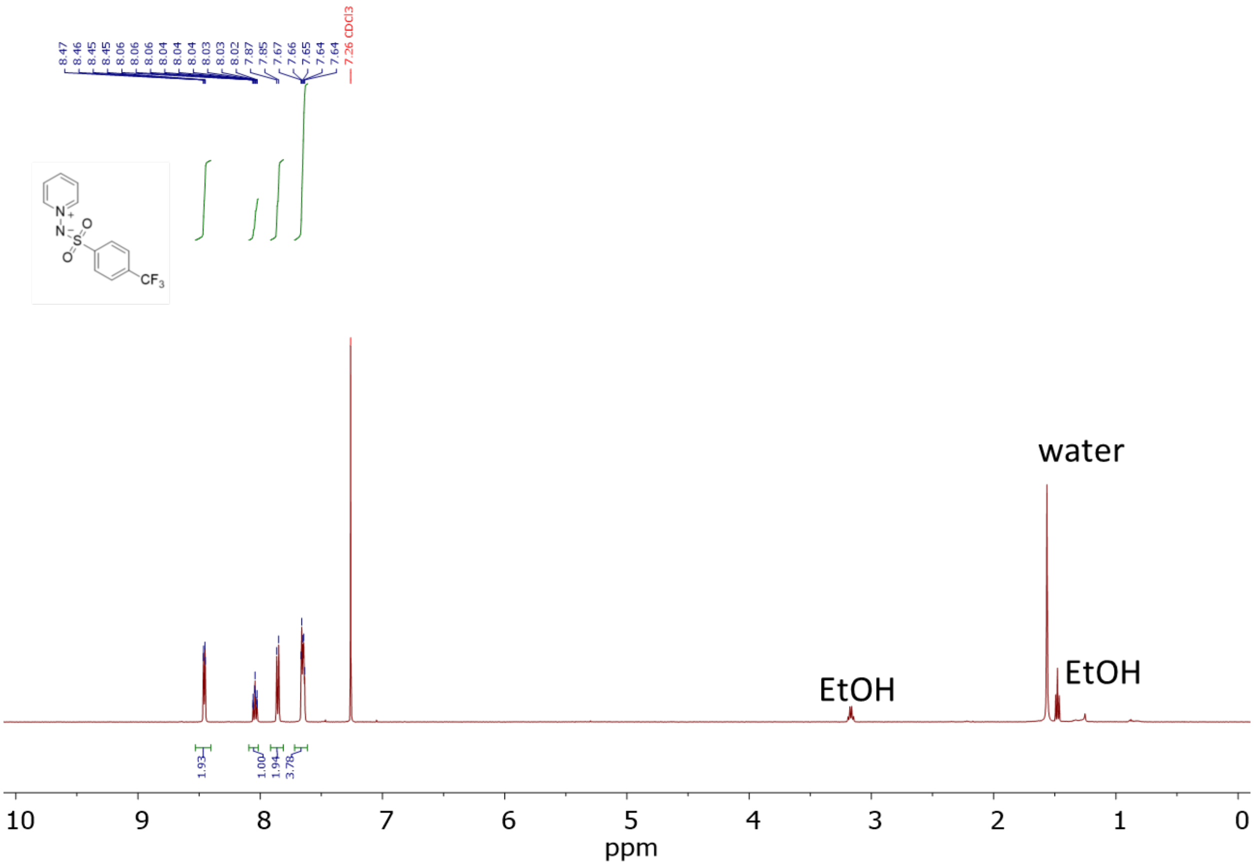

Figure S11. ^{1}H NMR of pyridinium sulfonamidate in $CDCl_3$ (400 MHz).

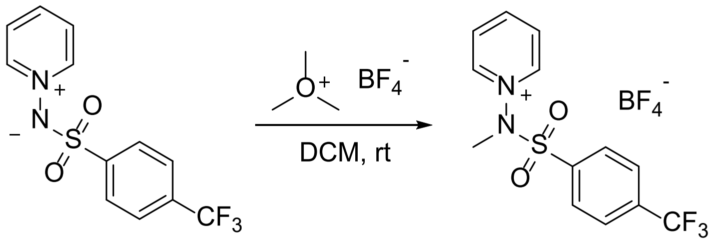

The pyridinium sulfonamidates (2.75 g, 9.1 mmol, 1 eq.) was dissolved in 90 mL of DCM. Trimethyloxonium tetrafluoroborate (1.48 g, 10 mmol, 1.1 eq.) was added to the solution at room temperature. After stirring for 24 hours at room temperature, the reaction mixture was concentrated under vacuum. The product was purified by recrystallization with diethyl ether from the DCM and MeOH (20:1) solution at -20 °C as white crystals. Yield: 3.42 g (93%). ^{1}H NMR (400 MHz, DMSO-d_6): 9.20 (d, J = 6.03 Hz, 2H), 8.86 (t, J = 7.53 Hz, 1H), 8.30 (t, J =7.53 Hz, 2H), 8.12 (d, J = 8.34 Hz, 2H), 8.02 (d, J = 8.34 Hz, 2H), 3.64 (s, 3H).

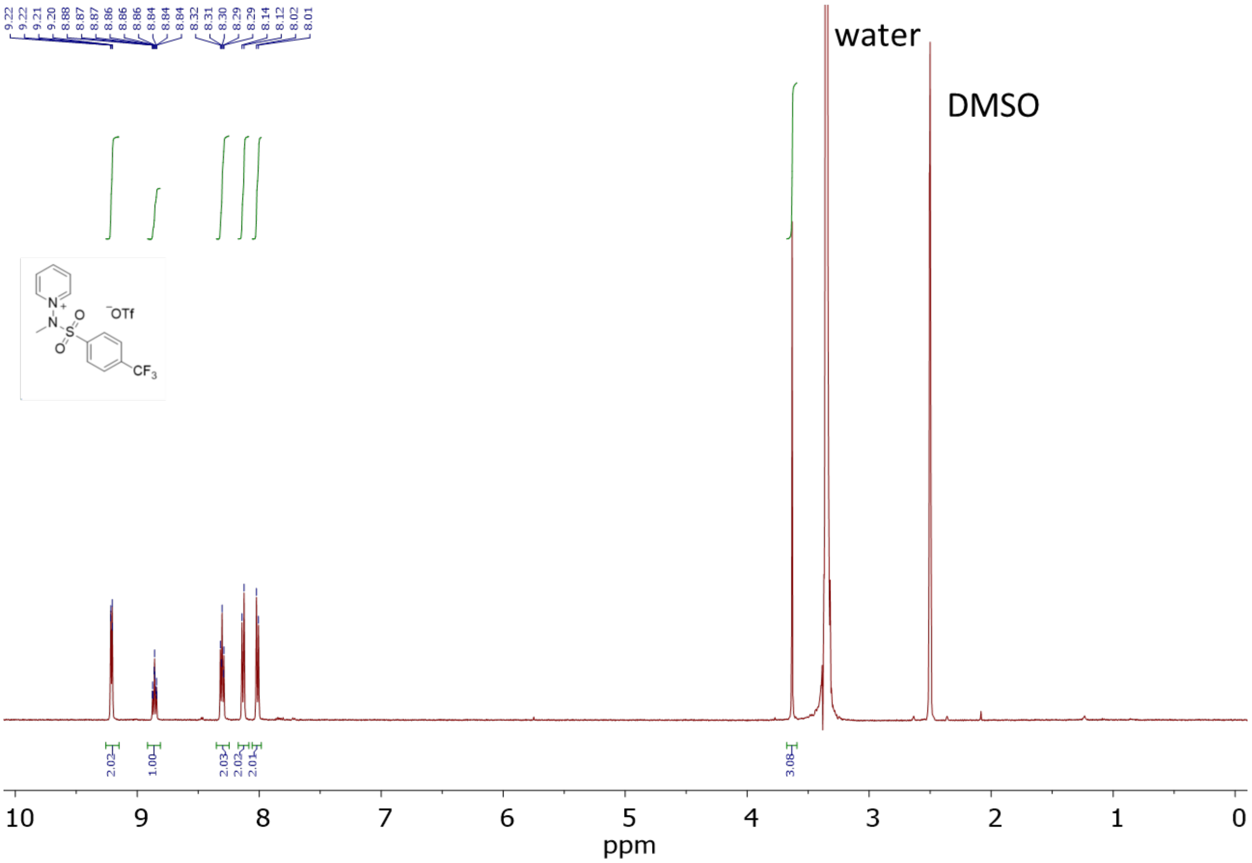

Figure S12. ^{1}H-NMR of the activated pyridine in DMSO-d_6 (400 MHz).

3. General Procedure for Catalytic Reactions and Control Experiments

General procedure for DAAQ-COF-catalyzed C-N coupling between C-H bonds and diethyl azodicarboxylate (DEAD).

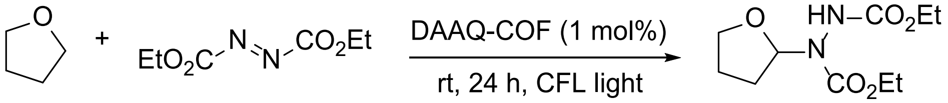

DAAQ-COF (2 μmol based on AQ sites) was dispersed in dry THF (2 mL). Diethyl azodicarboxylate (0.2 mmol) was added to the mixture. The resulting dispersion was stirred under CFL light irradiation at room temperature in an N_2 atmosphere for 24 hours. After the reaction, the

COF catalyst was removed by filtration, and the solvent was evaporated under vacuum. The product was purified by column chromatography using *n*-hexane and ethyl acetate as eluent.

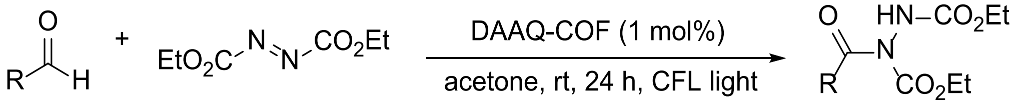

DAAQ-COF (2 μmol based on AQ sites) was dispersed in dry acetone (2 mL). Diethyl azodicarboxylate (0.2 mmol) and aldehyde (2.0 mmol) were added to the mixture. The resulting dispersion was stirred under CFL light irradiation at room temperature in an N_2 atmosphere for 24 hours. After the reaction, the COF catalyst was removed by filtration, and the solvent was evaporated under vacuum. The product was purified by column chromatography using *n*-hexane and ethyl acetate as eluent.

General procedure for DAAQ-COF-catalyzed C-C coupling between C-H bonds and activated pyridine.

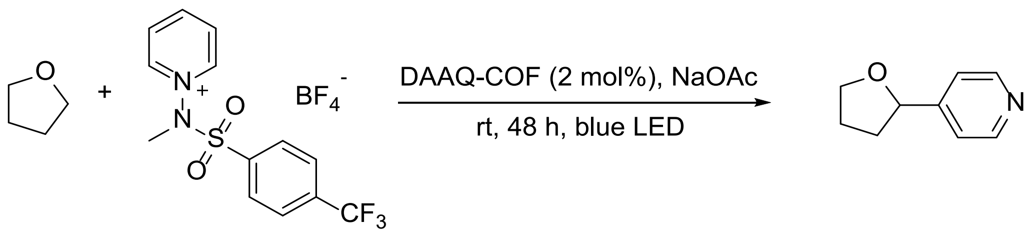

DAAQ-COF (4 μmol based on AQ sites), N-aminopyridinium salt (0.2 mmol), and sodium acetate (0.4 mmol) were dispersed in 2 mL dry THF. The resulting solution was stirred under blue LED irradiation at room temperature in an N_2 atmosphere for 48 hours. After the reaction, the COF catalyst was removed by filtration, and the solvent was evaporated under vacuum. The product was purified by column using *n*-hexane, ethyl acetate and 1% triethylamine as eluent.

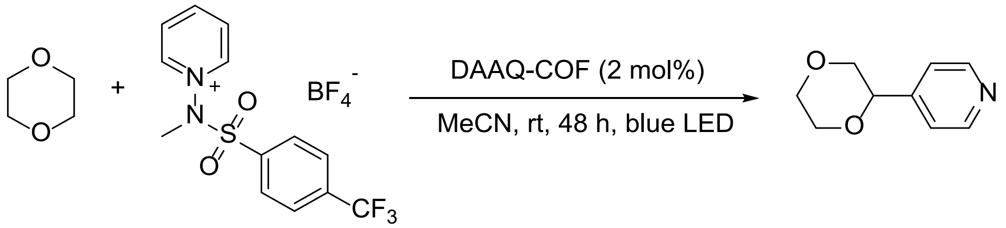

DAAQ-COF (4 μmol based on AQ sites), N-aminopyridinium salt (0.2 mmol), and sodium acetate (0.4 mmol) were dispersed in 1 mL of dry dioxane and 1 mL of dry acetonitrile. The resulting mixture was stirred under blue LED irradiation at room temperature in an N_2 atmosphere for 48 hours. After the reaction, the COF catalyst was removed by filtration, and the solvent was evaporated under vacuum. The product was purified by column using *n*-hexane, ethyl acetate and 1% triethylamine as eluent.

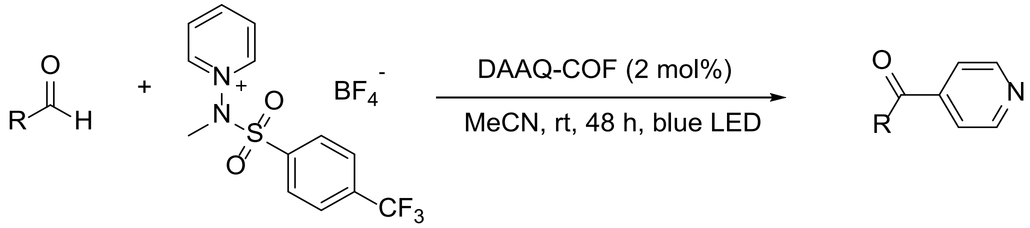

DAAQ-COF (4 μmol based on AQ sites), N-aminopyridinium salt (0.2 mmol), and sodium acetate (0.4 mmol) were dispersed in 2 mL of dry acetonitrile. Aldehyde (2.0 mmol) was added to the dispersion. The resulting mixture was stirred under blue LED irradiation at room temperature in an N_2 atmosphere for 48 hours. After the reaction, the COF catalyst was removed by filtration, and the solvent was evaporated under vacuum. The product was purified by column using *n*-hexane, ethyl acetate and 1% triethylamine as eluent.

General procedure of DAAQ-COF-catalyzed C-N coupling between THF and diethyl azodicarboxylate (DEAD) with TEMPO added as radical scavenger.

DAAQ-COF (2 μmol based on AQ sites) was dispersed in dry THF (2 mL). Diethyl azodicarboxylate (0.2 mmol) and TEMPO (0.2 mmol) was added to the mixture. The resulting dispersion was stirred under CFL light irradiation at room temperature in an N_2 atmosphere for 24 hours. After the reaction, the COF catalyst was removed by filtration, and the solvent was evaporated under vacuum. The residue was dissolved in $CHCl_3$-*d* for ^1H NMR analysis. The yield of the target product was 0%.

Recycle procedure in DAAQ-COF-catalyzed C-N coupling between THF and diethyl azodicarboxylate (DEAD).

DAAQ-COF (5 μmol based on AQ sites) was dispersed in dry THF (5 mL). Diethyl azodicarboxylate (0.1 mmol) was added to the mixture. The resulting dispersion was stirred under CFL light irradiation at room temperature in an N_2 atmosphere for 24 hours. After the reaction, the COF catalyst was collected via centrifugation and washed three times with THF to remove any organic species. The solvent of filtrate was evaporated under vacuum and the residue was analyzed by ^1H NMR. The recycled COF catalyst was directly used in the next reaction run.

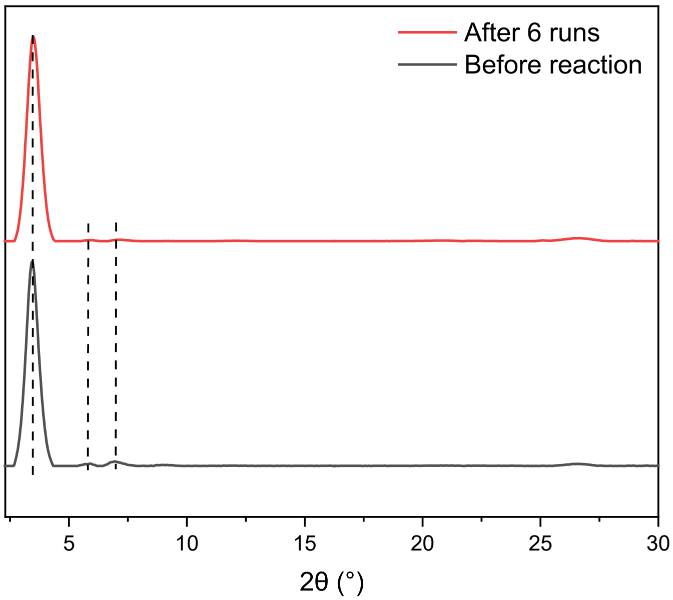

Figure S13. PXRD patterns of DAAQ-COF before the C-N coupling reaction between THF and DEAD and after 6 reaction runs.

Recycle procedure in DAAQ-COF-catalyzed C-N coupling between THF and activated pyridine.

DAAQ-COF (10 μmol based on AQ sites), N-aminopyridinium salt (0.1 mmol), and sodium acetate (0.2 mmol) were dispersed in 2 mL dry THF. The resulting solution was stirred under blue LED irradiation at room temperature in an N_2 atmosphere for 48 hours. After the reaction, the COF catalyst was collected via centrifugation and washed three times with THF to remove any organic species. The solvent of filtrate was evaporated under vacuum and the residue was analyzed by ^1H NMR. The recycled COF catalyst was directly used in the next reaction run.

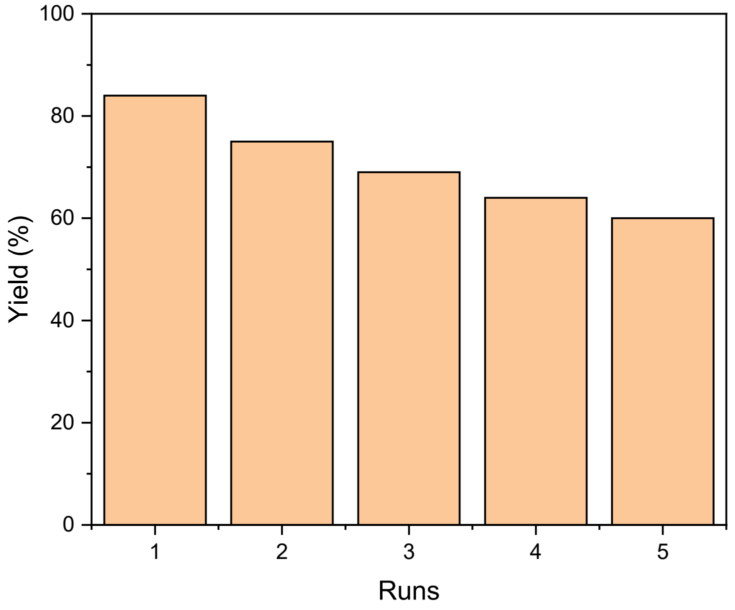

Figure S14. Yield of the C-C coupling product between N-aminopyridinium salt and THF in five consecutive runs. The decrease in the product yield is likely due to slow decomposition of DAAQ-COF under basic conditions.

General procedure for one-pot C-H functionalization

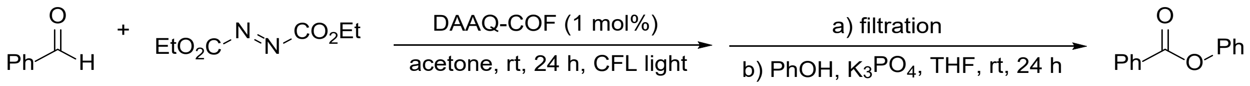

DAAQ-COF (2 μmol based on AQ sites) was dispersed in dry acetone (2 mL). Diethyl azodicarboxylate (0.2 mmol) and benzaldehyde (2.0 mmol) were added to the mixture. The resulting dispersion was stirred under CFL light irradiation at room temperature in an N_2 atmosphere for 24 hours. After the reaction, the COF catalyst was removed by filtration, and the solvent was evaporated under vacuum. K_3PO_4 (0.4 mmol), phenol (0.6 mmol), and THF (1 mL) were added to the residue. The mixture was stirred under N_2 at room temperature for 24 hours. The

generated phenyl benzoate was purified by column chromatography using *n*-hexane and ethyl acetate as eluent. The overall isolated yield after the two steps was 82%.

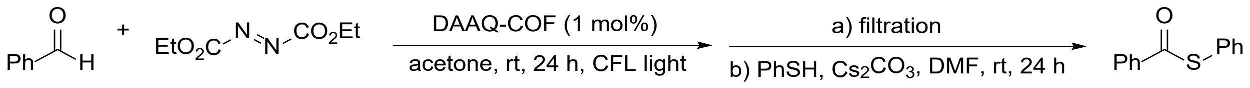

DAAQ-COF (2 μmol based on AQ sites) was dispersed in dry acetone (2 mL). Diethyl azodicarboxylate (0.2 mmol) and benzaldehyde (2.0 mmol) were added to the mixture. The resulting dispersion was stirred under CFL light irradiation at room temperature in an N_2 atmosphere for 24 hours. After the reaction, the COF catalyst was removed by filtration, and the solvent was evaporated under vacuum. Cs_2CO_3 (0.2 mmol), thiophenol (0.6 mmol) and DMF (1 mL) was added to the residue. The mixture was stirred under N_2 at room temperature for 24 hours. The generating S-phenyl benzothioate was purified by column chromatography using *n*-hexane and ethyl acetate as eluent. The overall isolated yield after the two steps was 90%.

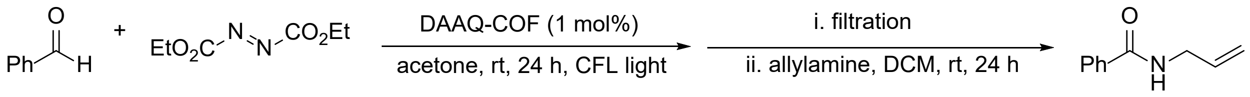

DAAQ-COF (2 μmol based on AQ sites) was dispersed in dry acetone (2 mL). Diethyl azodicarboxylate (0.2 mmol) and benzaldehyde (2.0 mmol) were added to the mixture. The resulting dispersion was stirred under CFL light irradiation at room temperature in an N_2 atmosphere for 24 hours. After the reaction, the COF catalyst was removed by filtration, and the solvent was evaporated under vacuum. allylamine (2.0 mmol) and DCM (1 mL) was added to the residue. The mixture was stirred under N_2 at room temperature for 24 hours. The generated N-allylbenzamide was purified by column chromatography using *n*-hexane and ethyl acetate as eluent. The overall isolated yield after the two steps was 85%.

4. Mechanistic study

Sample preparation for diffuse reflectance UV-vis spectroscopy

5 mg DAAQ-COF was mixed with 95 mg KBr (as a non-absorbing matrix) by grinding. The resulting powder was loaded into a Praying Mantis diffuse reflectance cell and the diffuse reflectance UV-vis spectrum was acquired on a CARY 5000 spectrophotometer. The Kubelka-Munk conversion of the raw diffuse reflectance spectrum was obtained by applying the formula $F(R) = (1-R)^2/2R$.

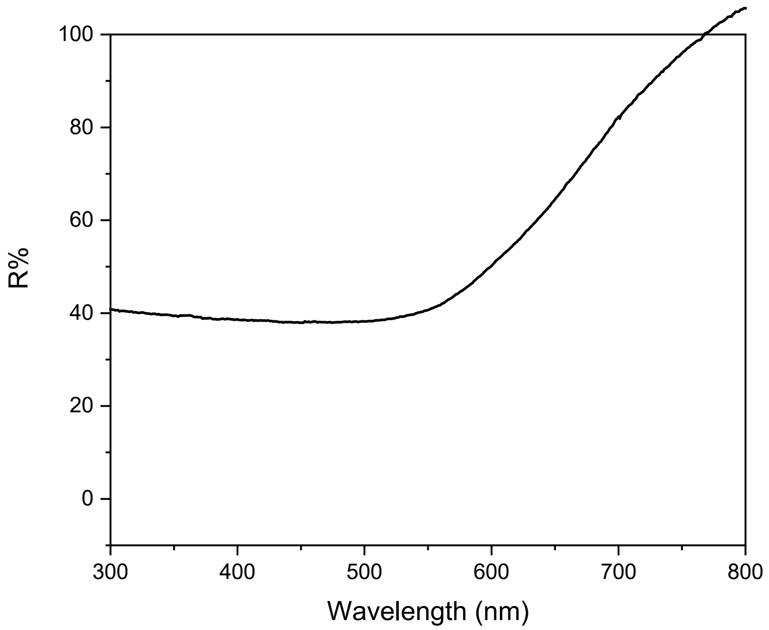

Figure S15. The original diffuse reflectance UV-vis spectrum of DAAQ-COF before the Kubelka-Munk conversion.

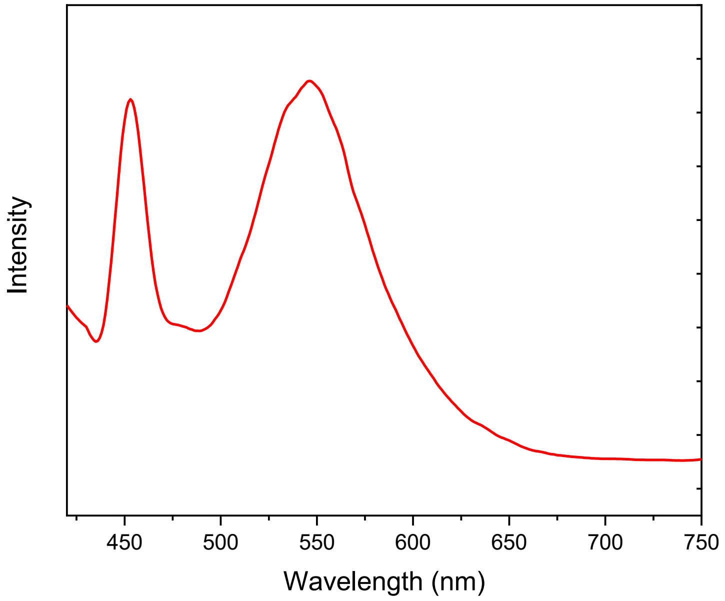

Figure S16. The emission spectrum of DAAQ-COF dispersion in acetone with 400 nm excitation.

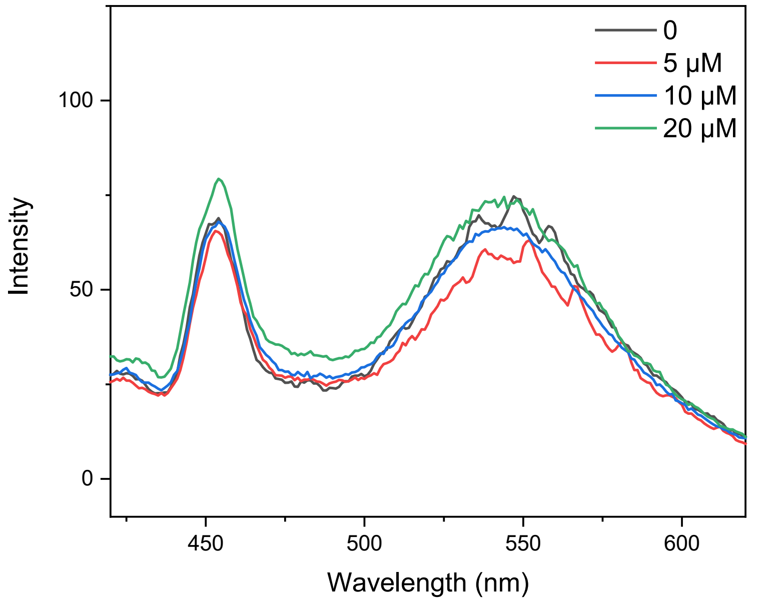

Figure S17. The emission spectra of DAAQ-COF dispersions in acetone with the addition of different concentrations of benzaldehyde (400 nm excitation). No significant quenching by benzaldehyde was observed, excluding the possibility of electron transfer between the photoexcited DAAQ-COF and benzaldehyde.

General procedure for intermolecular kinetic isotope effect (KIE) measurement

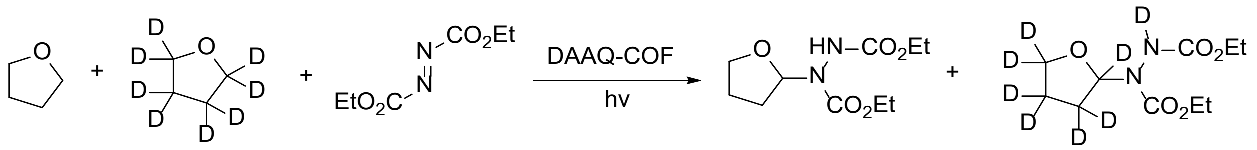

DAAQ-COF (2 μmol based on AQ sites) was dispersed in a mixture of 1 mL of THF and 1 mL of THF-d_8. Diethyl azodicarboxylate (0.2 mmol) was added to the mixture. The resulting dispersion was stirred under CFL light irradiation at room temperature in an N_2 atmosphere for 24 hours. After the reaction, the COF catalyst was removed by filtration, and the solvent was evaporated under vacuum. The residue was dissolved in $CHCl_3$-d for ^{1}H NMR analysis.

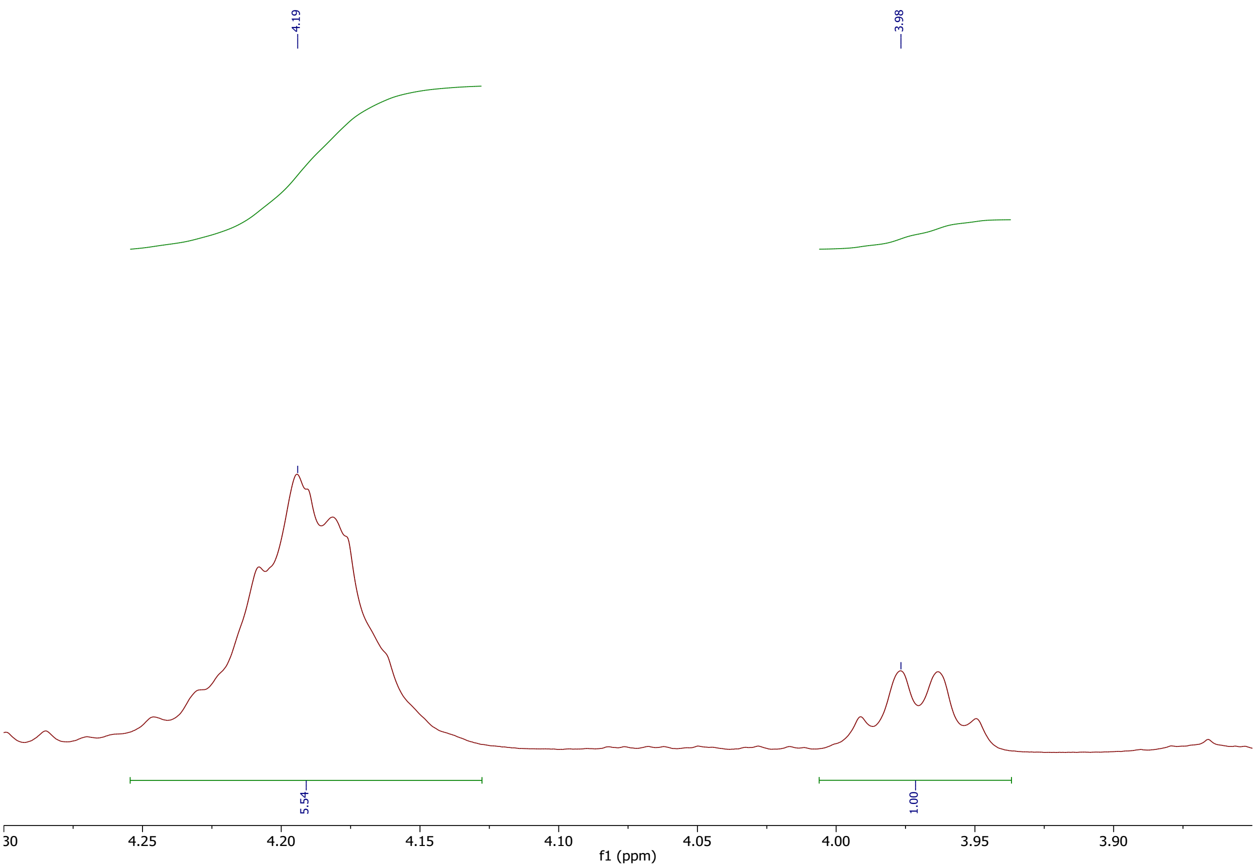

Figure S18. ^{1}H NMR spectrum of the reaction mixture after the KIE experiment. The ratio of the integrals between the two peaks is expected to be 4:1 in a non-deuterated C-N coupling product between THF and DEAD. Thus, the intermolecular KIE was calculated as:

$$\frac{k_H}{k_D} = \frac{[P_H]}{[P_D]} = \frac{1.00}{(\frac{5.54}{4} - 1.00)} = 2.60$$

This is a typical primary KIE, suggesting that the C-H bond cleavage is the rate-determining step in this reaction.

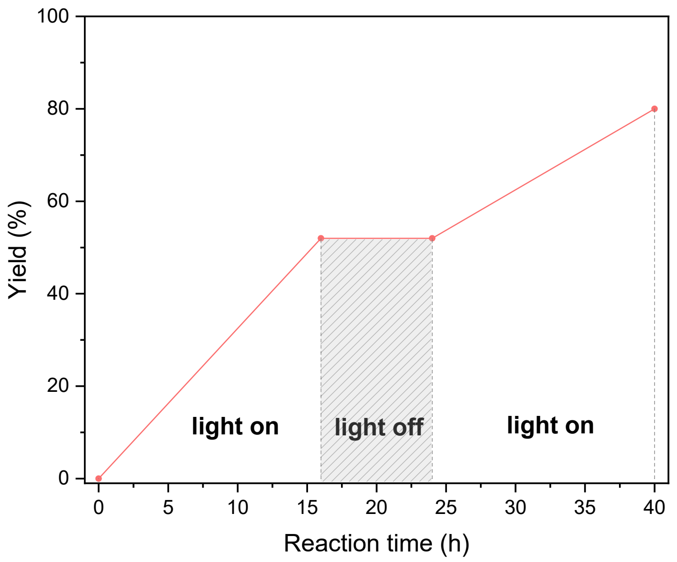

Figure S19. Light on/off experiment of the C-C coupling reaction between benzaldehyde and activated pyridine to rule out the involvement of a radical chain process in the reaction.

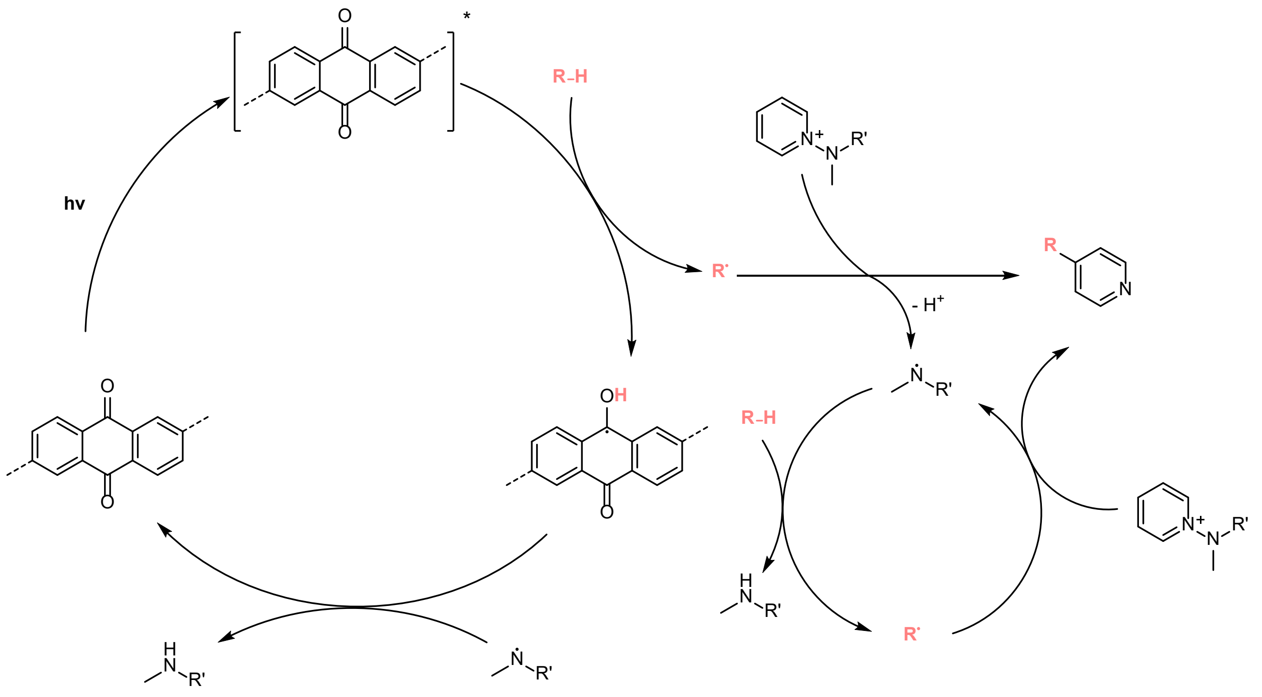

Figure S20. Proposed mechanism for C-C coupling reaction catalyzed by DAAQ-COF under visible light irradiation.

5. Product Characterization

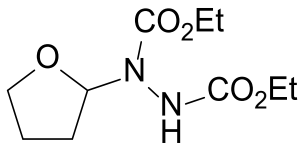

Yield: 94%. ^{1}H NMR (400 MHz, Chloroform-*d*): δ 6.43 (br, 1H), 5.99 (br, 1H), 4.21 (qt, J = 7.3, 4.5 Hz, 4H), 4.08 – 3.87 (q, J = 7.2 Hz, 1H), 3.76 (q, J = 7.2 Hz, 1H), 2.16 – 1.81 (m, 4H), 1.27 (m, J = 7.1, 2.9 Hz, 6H). ^{13}C NMR (101 MHz, Chloroform-*d*): δ 156.85, 155.70, 87.74, 68.84, 62.98, 62.30, 28.39, 25.43, 14.57, 14.53. HRMS (ESI, positive ion mode): calc'd for $[M+Na]^+$ ($C_{10}H_{18}N_2O_5Na$), 269.1113; observed, 269.1124.

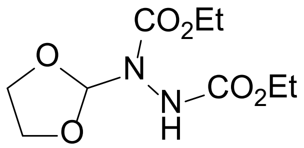

Yield: 58%. ^{1}H NMR (400 MHz, Chloroform-*d*): δ 6.67 (s, 1H), 6.46 (br, 1H), 4.20 (qd, *J* = 7.0, 4.2 Hz, 4H), 4.12 (dd, *J* = 14.6, 9.3 Hz, 2H), 3.94 (d, *J* = 5.8 Hz, 2H), 1.26 (m, *J* = 7.2, 3.1 Hz, 6H). ^{13}C NMR (101 MHz, Chloroform-*d*): 161.23, 154.51, 104.84, 65.51, 62.98, 62.28, 14.51, 14.43. HRMS (ESI, positive ion mode): calc'd for $[M+Na]^+$ ($C_9H_{16}N_2O_6Na$), 271.0906; observed, 271.0910.

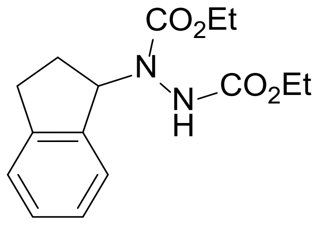

Yield: 50%. ^{1}H NMR (400 MHz, Chloroform-*d*): δ 7.33 – 7.15 (m, 4H), 6.17 (s, 1H), 5.86 (br, 1H), 4.35 – 4.04 (m, 4H), 2.98 (ddd, *J* = 13.7, 9.0, 4.4 Hz, 1H), 2.82 (dt, *J* = 15.9, 7.9 Hz, 1H), 2.41 (m, 1H), 2.19 (m, 1H), 1.25 (m, *J* = 39.0, 7.1 Hz, 6H). ^{13}C NMR (101 MHz, Chloroform-*d*): 156.78, 156.29, 144.18, 140.63, 128.24, 126.71, 125.12, 124.14, 62.79, 62.05, 30.53, 29.08, 14.65, 14.50. HRMS (ESI, positive ion mode): calc'd for $[M+Na]^+$ ($C_{15}H_{20}N_2O_4Na$), 315.1320; observed, 315.1320.

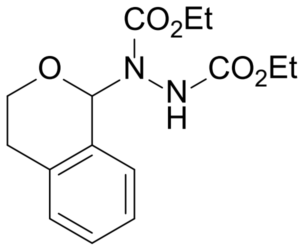

Yield: 75%. ^{1}H NMR (400 MHz, Chloroform-*d*): δ 7.39 (s, 1H), 7.22 (m, 2H), 7.11 (d, *J* = 6.7 Hz, 1H), 6.61 (s, 1H), 6.31 (d, *J* = 53.4 Hz, 1H), 4.35 – 4.24 (m, 2H), 4.18 – 3.93 (m, 4H), 2.93 (m, 1H), 2.73 (m, 1H), 1.30 (t, *J* = 7.1 Hz, 3H), 1.16 (m, 3H). ^{13}C NMR (101 MHz, Chloroform-*d*): δ 156.33, 155.74, 136.03, 135.63, 131.78, 128.52, 128.01, 126.54, 84.04, 63.27, 61.99, 29.83, 28.47, 14.51, 14.47. HRMS (ESI, positive ion mode): calc'd for $[M+Na]^+$ ($C_{15}H_{20}N_2O_5Na$), 331.1269; observed, 331.1269.

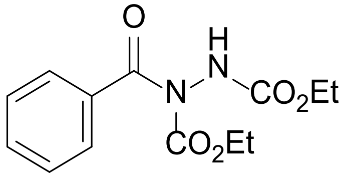

Yield: 95%. ^{1}H NMR (400 MHz, Chloroform-*d*): δ 7.69 (d, *J* = 7.7 Hz, 2H), 7.58 – 7.49 (m, 1H), 7.42 (tt, *J* = 6.6, 1.3 Hz, 2H), 6.97 (br, 1H), 4.25 (q, *J* = 7.1 Hz, 2H), 4.15 (q, *J* = 7.1 Hz, 2H), 1.30 (t, *J* = 7.1 Hz, 3H), 1.07 (t, *J* = 7.1 Hz, 3H). ^{13}C NMR (101 MHz, Chloroform-*d*): δ 171.21

153.56, 134.93, 132.22, 128.30, 64.13, 62.90, 29.84, 14.51, 13.87. HRMS (ESI, positive ion mode): calc'd for $[M+Na]^+$ ($C_{13}H_{16}N_2O_5Na$), 303.0957; observed, 303.0954.

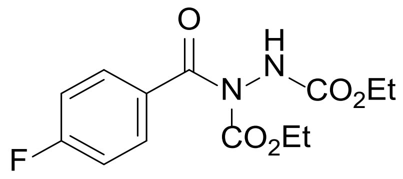

Yield: 98%. ^{1}H NMR (400 MHz, Chloroform-*d*): δ 7.73 (s, 2H), 7.17 – 7.05 (m, 2H), 6.98 (s, 1H), 4.25 (q, J = 7.1 Hz, 2H), 4.18 (q, J = 7.1 Hz, 2H), 1.30 (t, J = 7.1 Hz, 3H), 1.13 (t, J = 7.1 Hz, 3H). ^{13}C NMR (101 MHz, Chloroform-*d*): δ 170.11, 165.50 (d, J = 254 Hz, 1C), 155.74, 153.52, 131.14 (d, J = 8.9 Hz, 1C), 115.67, 115.45, 64.26, 62.96, 14.51, 13.99. ^{19}F {^{1}H} NMR (377 MHz, Chloroform-*d*): δ -106.04. HRMS (ESI, positive ion mode): calc'd for $[M+Na]^+$ ($C_{13}H_{15}FN_2O_5Na$), 321.0862; observed, 321.0848.

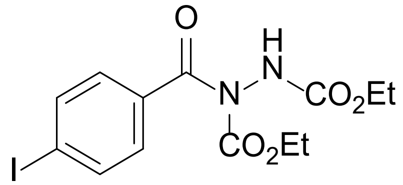

Yield: 75%. ^{1}H NMR (400 MHz, Chloroform-*d*): δ 7.81 – 7.74 (m, 2H), 7.48 – 7.29 (m, 2H), 7.02 (s, 1H), 4.24 (q, J = 7.1 Hz, 2H), 4.18 (q, J = 7.1 Hz, 2H), 1.30 (t, J = 7.2 Hz, 3H), 1.14 (t, J = 7.1 Hz, 3H). ^{13}C {^{1}H} NMR (101 MHz, Chloroform-*d*): δ 170.60, 155.69, 153.38, 137.55, 134.29, 131.63, 129.75, 64.33, 62.96, 14.48, 13.96. HRMS (ESI, positive ion mode): calc'd for $[M+Na]^+$ ($C_{13}H_{15}IN_2O_5Na$), 428.9923; observed, 428.9909.

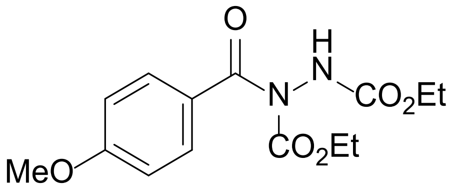

Yield: 65%. ^{1}H NMR (400 MHz, Chloroform-*d*): δ 7.73 (d, J = 8.3 Hz, 2H), 6.94 – 6.88 (m, 2H), 4.24 (q, J = 7.1 Hz, 2H), 4.18 (q, J = 7.1 Hz, 2H), 3.86 (s, 3H), 1.32 – 1.27 (m, 3H), 1.14 (t, J = 7.1 Hz, 3H). ^{13}C {^{1}H} NMR (101 MHz, Chloroform-*d*): δ 163.29, 155.82, 153.90, 132.44, 131.24, 113.91, 113.64, 64.02, 62.82, 55.61, 14.53, 13.08. HRMS (ESI, positive ion mode): calc'd for $[M+Na]^+$ ($C_{14}H_{18}N_2O_6Na$), 333.1063; observed, 333.1063.

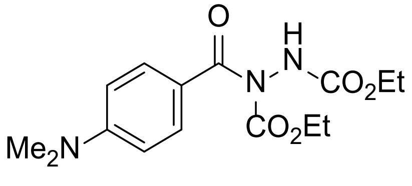

Yield: 82%. ^{1}H NMR (400 MHz, Chloroform-*d*): δ 7.71 (d, J = 8.6 Hz, 2H), 6.96 (s, 1H), 6.68 – 6.56 (m, 2H), 4.21 (m, 4H), 3.05 (s, 6H), 1.28 (m, 3H), 1.18 (t, J = 7.1 Hz, 3H). ^{13}C {^{1}H} NMR (101 MHz, Chloroform-*d*): δ 155.94, 154.36, 153.64, 131.89, 120.29, 110.57, 63.70, 62.61, 40.18, 14.54, 14.21. HRMS (ESI, positive ion mode): calc'd for $[M+Na]^+$ ($C_{15}H_{21}N_3O_5Na$), 346.1379; observed, 346.1389.

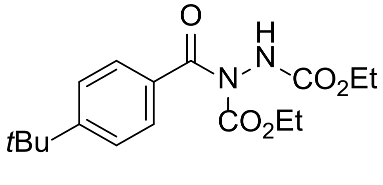

Yield: 85%. ^{1}H NMR (400 MHz, Chloroform-*d*): δ 7.64 (d, J = 7.9 Hz, 2H), 7.42 (d, J = 8.4 Hz, 2H), 7.13 (s, 1H), 4.24 (q, J = 7.1 Hz, 2H), 4.15 (q, J = 7.1 Hz, 2H), 1.32 (m, 12H), 1.05 (t, J = 7.2 Hz, 3H). ^{13}C {^{1}H} NMR (101 MHz, Chloroform-*d*): δ 156.15, 155.92, 153.85, 131.91, 130.26, 128.58, 125.30, 77.26, 64.06, 62.87, 35.27, 31.32, 14.58, 13.90. HRMS (ESI, positive ion mode): calc'd for $[M+Na]^+$ ($C_{17}H_{24}N_2O_5Na$), 359.1583; observed, 359.1589.

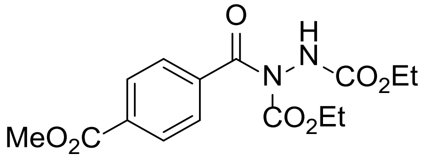

Yield: 65%. ^{1}H NMR (400 MHz, Chloroform-*d*): δ 8.11 (d, *J* = 8.4 Hz, 2H), 7.83 – 7.61 (m, 2H), 7.07 (s, 1H), 4.28 (q, *J* = 7.1 Hz, 2H), 4.17 (q, *J* = 7.1 Hz, 2H), 3.96 (s, 3H), 1.36 – 1.30 (m, 3H), 1.10 (t, *J* = 7.1 Hz, 3H). ^{13}C {^{1}H} NMR (101 MHz, Chloroform-*d*): 170.35, 166.30, 155.66, 153.20, 139.09, 132.97, 129.53, 127.92, 77.16, 64.37, 63.01, 52.57, 14.49, 13.90. HRMS (ESI, positive ion mode): calc'd for $[M+Na]^+$ ($C_{15}H_{18}N_2O_7Na$), 361.1012; observed, 361.1016.

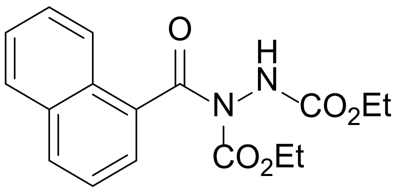

Yield: 87%. ^{1}H NMR (400 MHz, Chloroform-*d*): δ 8.17 (d, *J* = 8.3 Hz, 1H), 7.94 (d, *J* = 8.2 Hz, 1H), 7.91 – 7.82 (m, 1H), 7.65 (d, *J* = 7.2 Hz, 1H), 7.59 – 7.43 (m, 3H), 7.08 (s, 1H), 4.30 (q, *J* = 7.1 Hz, 2H), 3.94 (q, *J* = 7.2 Hz, 2H), 1.34 (t, *J* = 7.1 Hz, 3H), 0.73 (t, *J* = 7.2 Hz, 3H). ^{13}C {^{1}H} NMR (101 MHz, Chloroform-*d*): δ 170.46, 155.81, 152.82, 133.72, 133.32, 130.91, 130.02, 128.47, 127.58, 126.59, 125.09, 124.72, 64.10, 62.98, 14.54, 13.44. HRMS (ESI, positive ion mode): calc'd for $[M+Na]^+$ ($C_{17}H_{18}N_2O_5Na$), 353.1113; observed, 353.1112.

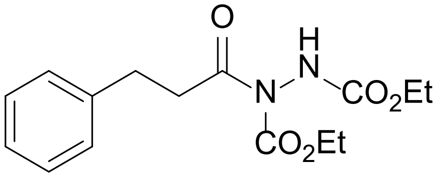

Yield: 98%. ^{1}H NMR (400 MHz, Chloroform-*d*): δ 7.31 – 7.26 (m, 2H), 7.22 – 7.15 (m, 3H), 6.90 (s, 1H), 4.28 (q, *J* = 7.1 Hz, 2H), 4.21 (d, *J* = 7.5 Hz, 2H), 3.24 (t, *J* = 7.9 Hz, 2H), 2.99 (t, *J*

= 7.8 Hz, 2H), 1.32 (m, 6H). $^{13}C\ \{^1H\}$ NMR (101 MHz, Chloroform-*d*): δ 177.45, 155.74, 153.21, 140.65, 128.54, 128.34, 126.27, 64.05, 62.70, 38.76, 30.66, 14.43, 14.22. HRMS (ESI, positive ion mode): calc'd for $[M+Na]^+$ ($C_{15}H_{20}N_2O_5Na$), 331.1270; observed, 331.1256.

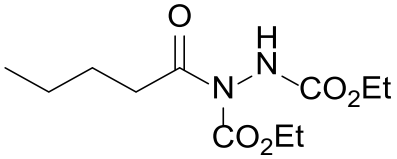

Yield: 82%. ^1H NMR (400 MHz, Chloroform-*d*): δ 6.69 (s, 1H), 4.29 (q, *J* = 7.1 Hz, 2H), 4.21 (q, *J* = 7.1 Hz, 2H), 2.90 (d, *J* = 7.8 Hz, 2H), 1.65 (p, *J* = 7.4 Hz, 2H), 1.42 – 1.25 (m, 8H), 0.92 (t, *J* = 7.3 Hz, 3H). $^{13}C\ \{^1H\}$ NMR (101 MHz, Chloroform-*d*): δ 174.00, 155.69, 153.33, 63.99, 62.69, 36.84, 26.80, 22.33, 14.50, 14.29, 13.95. HRMS (ESI, positive ion mode): calc'd for $[M+Na]^+$ ($C_{11}H_{20}N_2O_5Na$), 283.1270; observed, 283.1267.

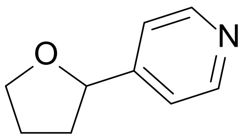

Yield: 86%. ^1H NMR (400 MHz, Chloroform-*d*): δ 8.54 (d, *J* = 5.6 Hz, 2H), 7.26 – 7.24 (m, 2H), 4.89 (t, *J* = 7.2 Hz, 1H), 4.08 (dt, *J* = 8.2, 6.7 Hz, 1H), 4.01 – 3.90 (m, 1H), 2.44 – 2.30 (m, 1H), 2.07 – 1.91 (m, 2H), 1.75 (dq, *J* = 12.3, 7.6 Hz, 1H). $^{13}C\ \{^1H\}$ NMR (101 MHz, Chloroform-*d*): δ 153.1, 149.64, 120.67, 79.18, 69.08, 34.43, 25.94. HRMS (ESI, positive ion mode): calc'd for $[M+H]^+$ ($C_9H_{12}NO$), 150.0919; observed,150.0921.

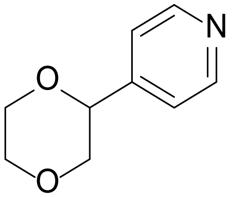

Yield: 53%. ^{1}H NMR (400 MHz, Chloroform-*d*): δ 8.62 – 8.55 (m, 2H), 7.30 – 7.25 (m, 2H), 4.63 (dd, *J* = 10.1, 2.8 Hz, 1H), 3.99 – 3.86 (m, 3H), 3.84 – 3.78 (m, 1H), 3.71 (ddd, *J* = 11.8, 10.9, 3.3 Hz, 1H), 3.38 (dd, *J* = 11.6, 10.1 Hz, 1H). ^{13}C {^{1}H} NMR (101 MHz, Chloroform-*d*): δ 150.08, 147.07, 121.01, 76.41, 71.98, 67.00, 66.49. HRMS (ESI, positive ion mode): calc'd for $[M+H]^+$ ($C_9H_{12}NO_2$), 166.0868; observed, 166.0869.

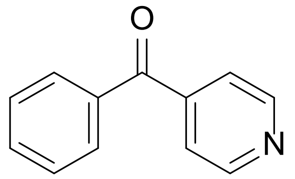

Yield: 75%. ^{1}H NMR (400 MHz, Chloroform-*d*): δ 8.86 – 8.75 (m, 2H), 7.87 – 7.78 (m, 2H), 7.71 – 7.62 (m, 1H), 7.62 – 7.56 (m, 2H), 7.56 – 7.49 (m, 2H). ^{13}C {^{1}H} NMR (101 MHz, Chloroform-*d*): δ 195.33, 150.53, 144.52, 136.06, 133.69, 130.29, 128.81, 123.01. HRMS (ESI, positive ion mode): calc'd for $[M+H]^+$ ($C_{12}H_{10}NO$), 184.0762; observed, 184.0762.

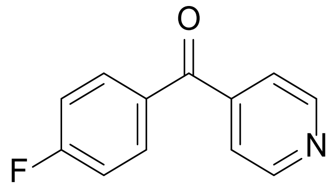

Yield: 90%. ^{1}H NMR (400 MHz, Chloroform-*d*): δ 8.88 – 8.78 (m, 2H), 7.91 – 7.82 (m, 2H), 7.62 – 7.49 (m, 2H), 7.23 – 7.14 (m, 2H). ^{13}C {^{1}H} NMR (101 MHz, Chloroform-*d*): δ 193.80, 167.45, 164.90, 150.60, 144.45, 132.96, 122.85, 116.23 (d, *J* = 21.9 Hz, 1C). ^{19}F {^{1}H} NMR (377 MHz, Chloroform-*d*): δ -103.67. HRMS (ESI, positive ion mode): calc'd for $[M+H]^+$ ($C_{12}H_9FNO$), 202.0668; observed, 202.0674.

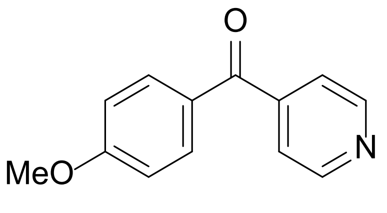

Yield: 82%. ^{1}H NMR (400 MHz, Chloroform-*d*): δ 8.82 – 8.73 (m, 2H), 7.87 – 7.80 (m, 2H), 7.59 – 7.48 (m, 2H), 7.02 – 6.93 (m, 2H), 3.89 (s, 3H). ^{13}C {^{1}H} NMR (101 MHz, Chloroform-*d*): δ 193.88, 164.19, 150.35, 145.39, 132.80, 128.76, 122.85, 114.09, 55.73. HRMS (ESI, positive ion mode): calc'd for $[M+H]^+$ ($C_{13}H_{12}NO_2$), 214.0868; observed, 214.0860.

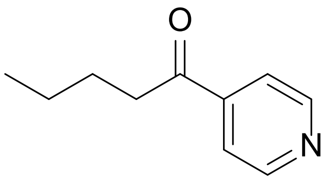

Yield: 96%. ^{1}H NMR (400 MHz, Chloroform-*d*): δ 8.86 – 8.74 (m, 2H), 7.77 – 7.68 (m, 2H), 2.96 (t, *J* = 7.3 Hz, 2H), 1.75 – 1.70 (m, 2H), 1.47 – 1.35 (m, 2H), 0.95 (t, *J* = 7.4 Hz, 3H). ^{13}C {^{1}H} NMR (101 MHz, Chloroform-*d*): δ 199.98, 151.07, 143.00, 121.21, 38.73, 26.08, 22.47, 14.02. HRMS (ESI, positive ion mode): calc'd for $[M+H]^+$ ($C_{10}H_{14}NO$), 164.1075; observed, 164.1073.

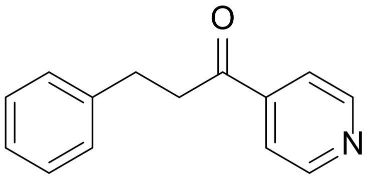

Yield: 93%. ^{1}H NMR (400 MHz, Chloroform-*d*): δ 8.84 – 8.73 (m, 2H), 7.75 – 7.65 (m, 2H), 7.30 (t, *J* = 7.6 Hz, 2H), 7.28 – 7.18 (m, 3H), 3.30 (dd, *J* = 8.2, 7.0 Hz, 2H), 3.07 (t, *J* = 7.6 Hz, 2H). ^{13}C {^{1}H} NMR (101 MHz, Chloroform-*d*): δ 198.75, 151.10, 142.71, 140.74, 128.75,

128.51, 126.49, 121.12, 40.84, 20.81. HRMS (ESI, positive ion mode): calc'd for $[M+H]^+$ ($C_{14}H_{14}NO$), 212.1075; observed, 212.1077.

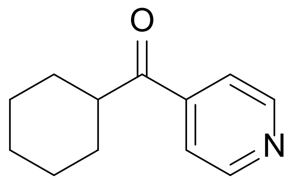

Yield: 65%. ^{1}H NMR (400 MHz, Chloroform-*d*): δ 8.89 – 8.70 (m, 2H), 7.80 – 7.62 (m, 2H), 3.19 (tt, *J* = 11.3, 3.3 Hz, 1H), 1.93 – 1.79 (m, 4H), 1.78 – 1.70 (m, 1H), 1.52 – 1.26 (m, 5H). ^{13}C {^{1}H} NMR (101 MHz, Chloroform-*d*): δ 203.28, 151.03, 142.61, 121.49, 46.15, 29.15, 25.95, 25.79. HRMS (ESI, positive ion mode): calc'd for $[M+H]^+$ ($C_{12}H_{16}NO$), 190.1232; observed, 190.1228.

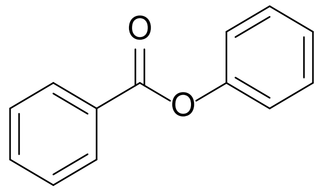

Yield: 82%. ^{1}H NMR (400 MHz, Chloroform-*d*): δ 8.26 – 8.18 (m, 2H), 7.67 – 7.61 (m, 1H), 7.52 (dd, *J* = 8.4, 7.1 Hz, 2H), 7.48 – 7.40 (m, 2H), 7.29 (m, 1H), 7.25 – 7.19 (m, 2H). ^{13}C {^{1}H} NMR (101 MHz, Chloroform-*d*): δ 165.34, 151.12, 133.73, 130.33, 129.75, 129.64, 128.72, 126.04, 121.87. HRMS (ESI, positive ion mode): calc'd for $[M+Na]^+$ ($C_{13}H_{10}O_2Na$), 221.0578; observed, 221.0585

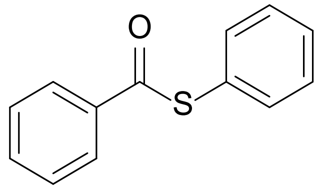

Yield: 90%. ^{1}H NMR (400 MHz, Chloroform-*d*): δ 8.07 – 8.02 (m, 2H), 7.62 (t, *J* = 7.4 Hz, 1H), 7.58 – 7.43 (m, 7H). ^{13}C {^{1}H} NMR (101 MHz, Chloroform-*d*): δ 190.28, 136.80, 135.24, 133.80, 129.67, 129.40, 128.90, 127.63, 127.50. HRMS (ESI, positive ion mode): calc'd for $[M+Na]^+$ ($C_{13}H_{10}OSNa$), 237.0350; observed, 237.0359.

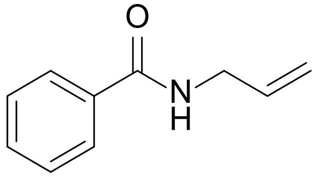

Yield: 85%. ^{1}H NMR (400 MHz, Chloroform-*d*): δ 7.85 – 7.73 (m, 2H), 7.55 – 7.47 (m, 1H), 7.43 (dd, *J* = 8.3, 6.8 Hz, 2H), 6.24 (s, 1H), 5.94 (ddt, *J* = 17.0, 10.1, 5.7 Hz, 1H), 5.27 (dq, *J* = 17.2, 1.6 Hz, 1H), 5.19 (dq, *J* = 10.3, 1.4 Hz, 1H), 4.09 (tt, *J* = 5.8, 1.6 Hz, 2H). ^{13}C NMR (101 MHz, Chloroform-*d*): δ 167.46, 134.62, 134.30, 131.64, 128.73, 127.04, 116.84, 77.16, 42.57. HRMS (ESI, positive ion mode): calc'd for $[M+Na]^+$ ($C_{10}H_{11}NO$), 184.0738; observed, 184.0740.

References

[1] C. R. DeBlase, K. E. Silberstein, T.-T. Truong, H. D. Abruña, W. R. Dichtel, *J. Am. Chem. Soc.* **2013**, *135*, 16821-16824.

[2] J. Duan, W. Wang, D. Zou, J. Liu, N. Li, J. Weng, L.-p. Xu, Y. Guan, Y. Zhang, P. Zhou, *ACS Applied Materials & Interfaces* **2022**, *14*, 31234-31244.

[3] W. Lee, S. Jung, M. Kim, S. Hong, *J. Am. Chem. Soc.* **2021**, *143*, 3003-3012.

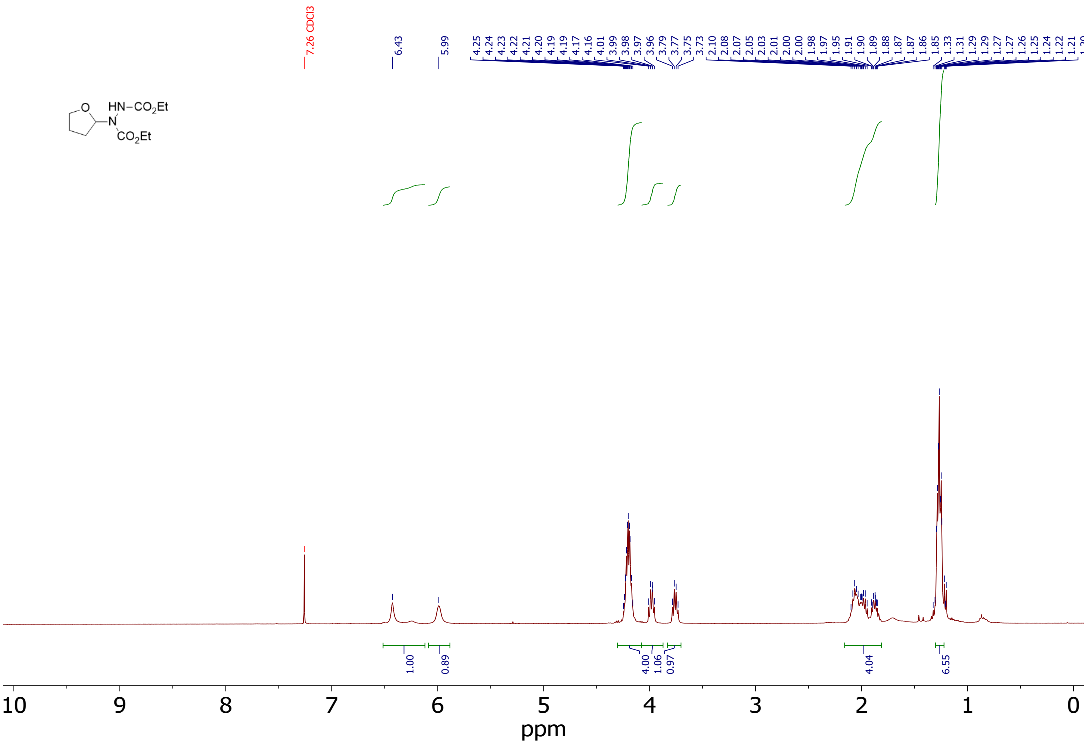

Figure S21. ^{1}H NMR spectrum of **3a** in $CDCl_3$ (400 MHz).

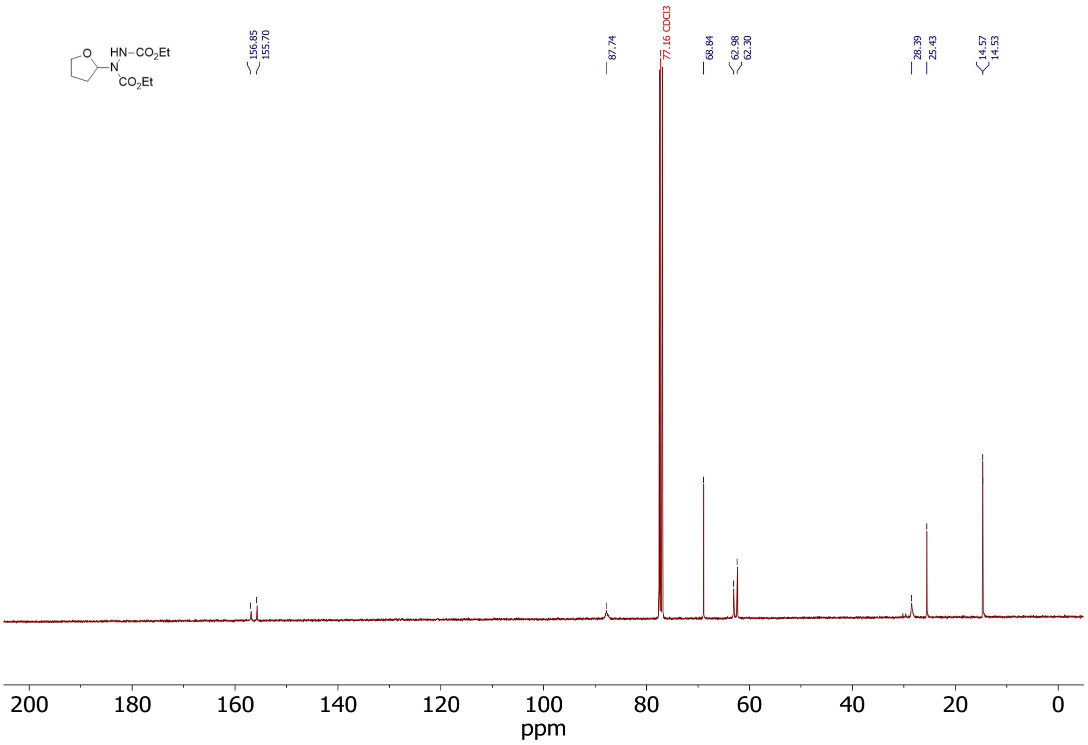

Figure S22. $^{13}C\{^{1}H\}$ NMR spectrum of **3a** in $CDCl_3$ (101 MHz).

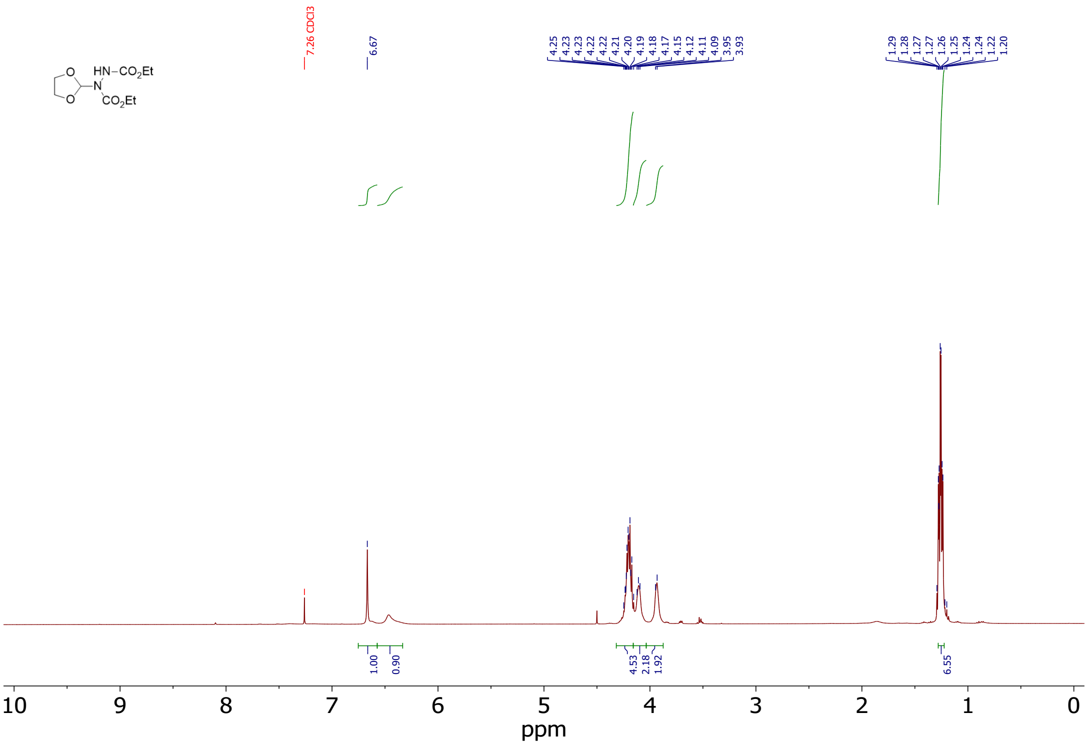

Figure S23. ^{1}H NMR spectrum of **3b** in $CDCl_3$ (400 MHz).

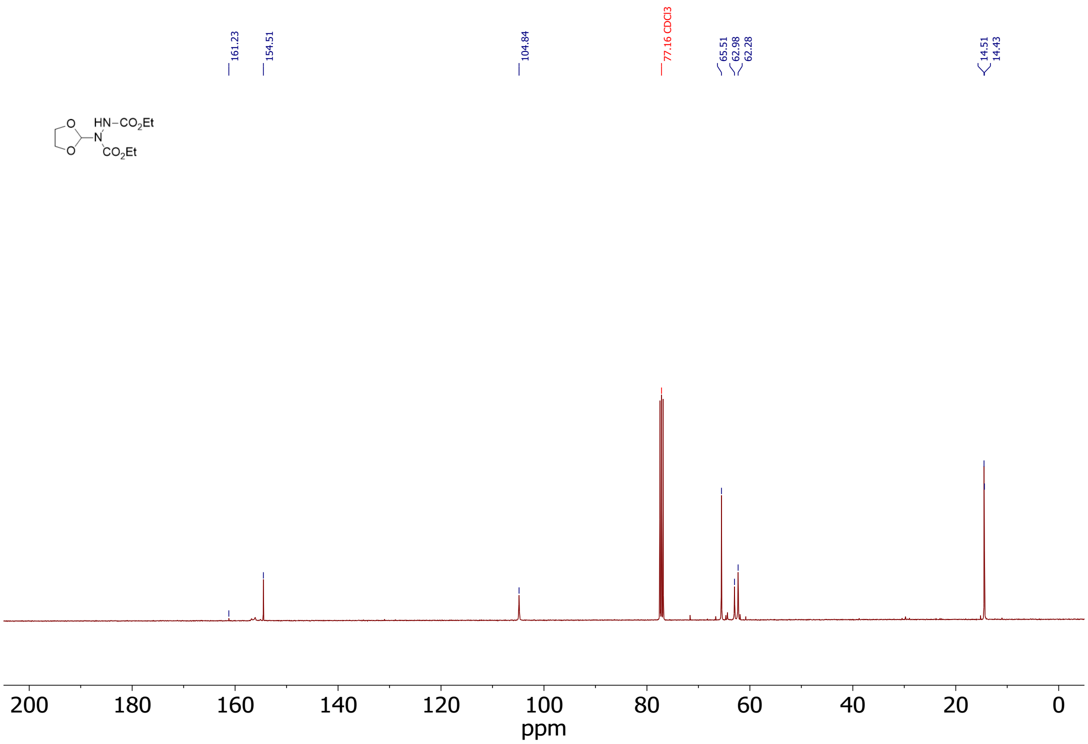

Figure S24. $^{13}C\{^{1}H\}$ NMR spectrum of **3b** in $CDCl_3$ (101 MHz).

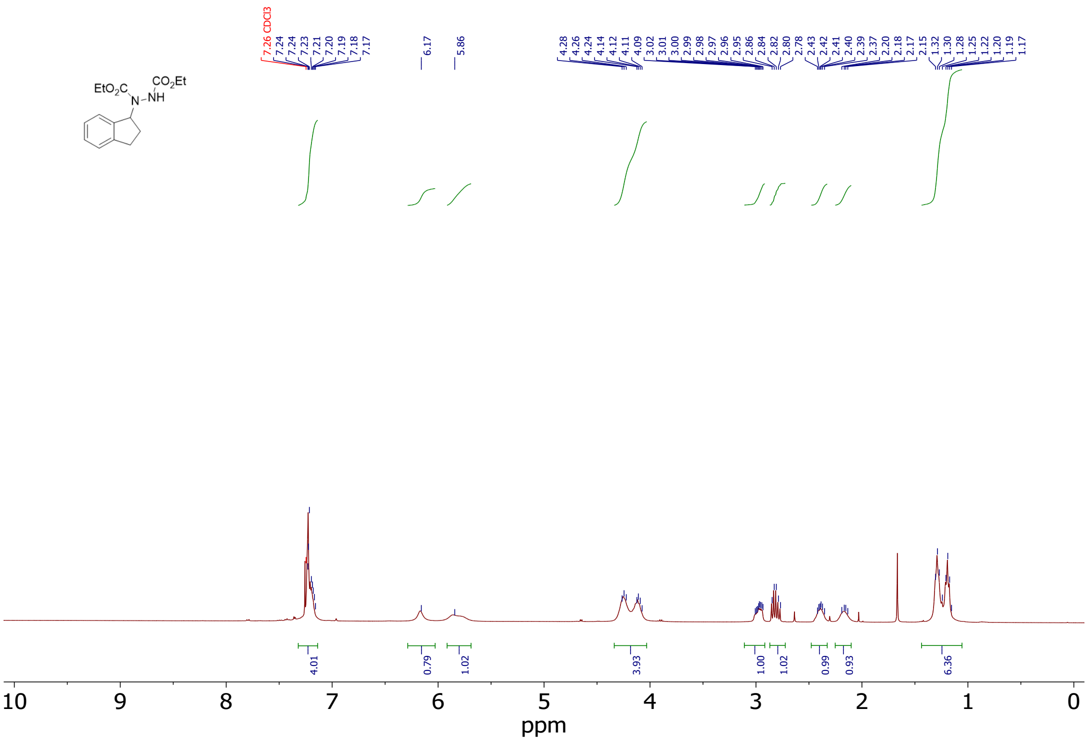

Figure S25. ^{1}H NMR spectrum of **3c** in $CDCl_3$ (400 MHz).

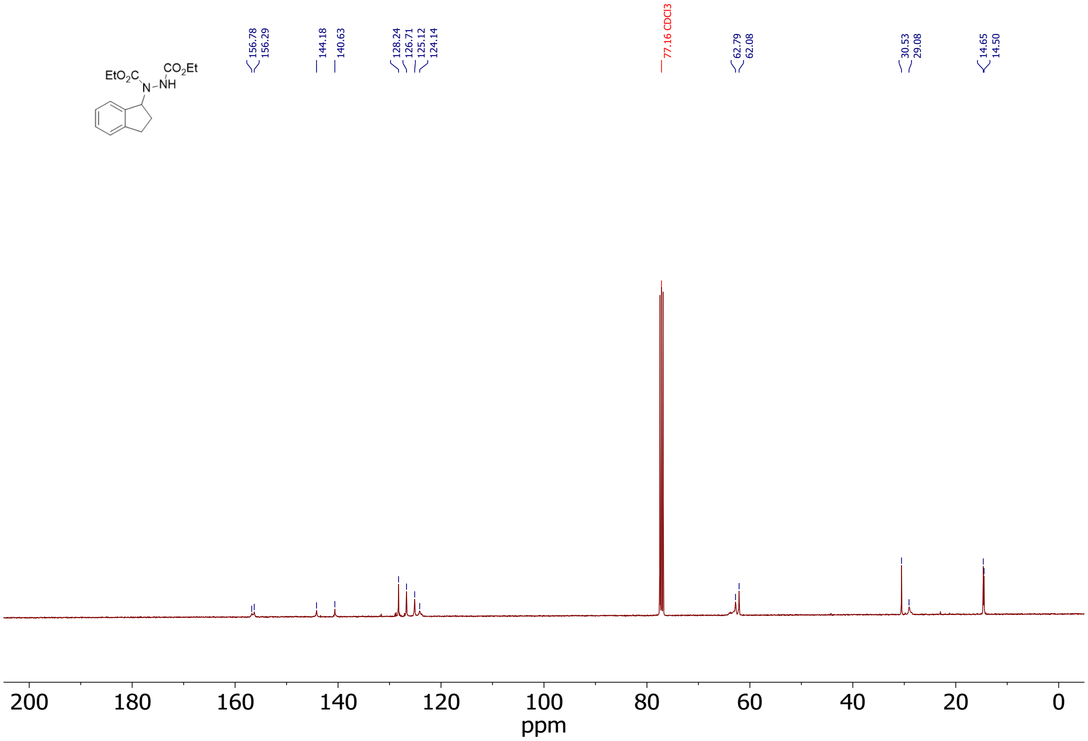

Figure S26. $^{13}C\{^{1}H\}$ NMR spectrum of **3c** in $CDCl_3$ (101 MHz).

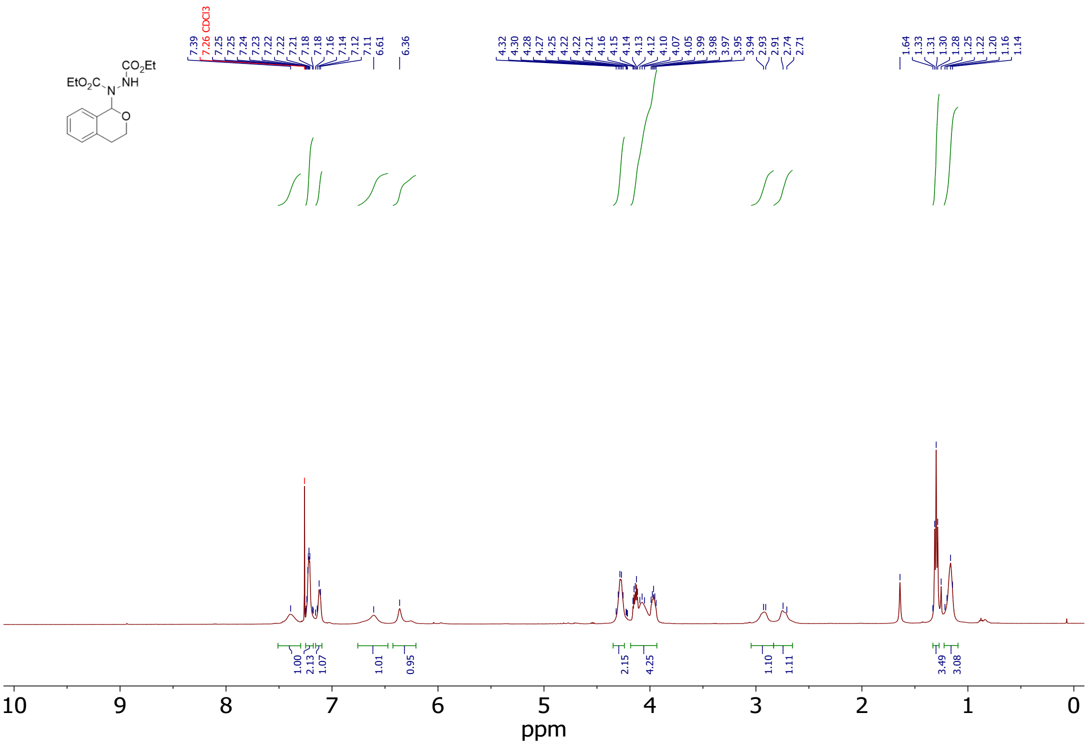

Figure S27. ^{1}H NMR spectrum of **3d** in $CDCl_3$ (400 MHz).

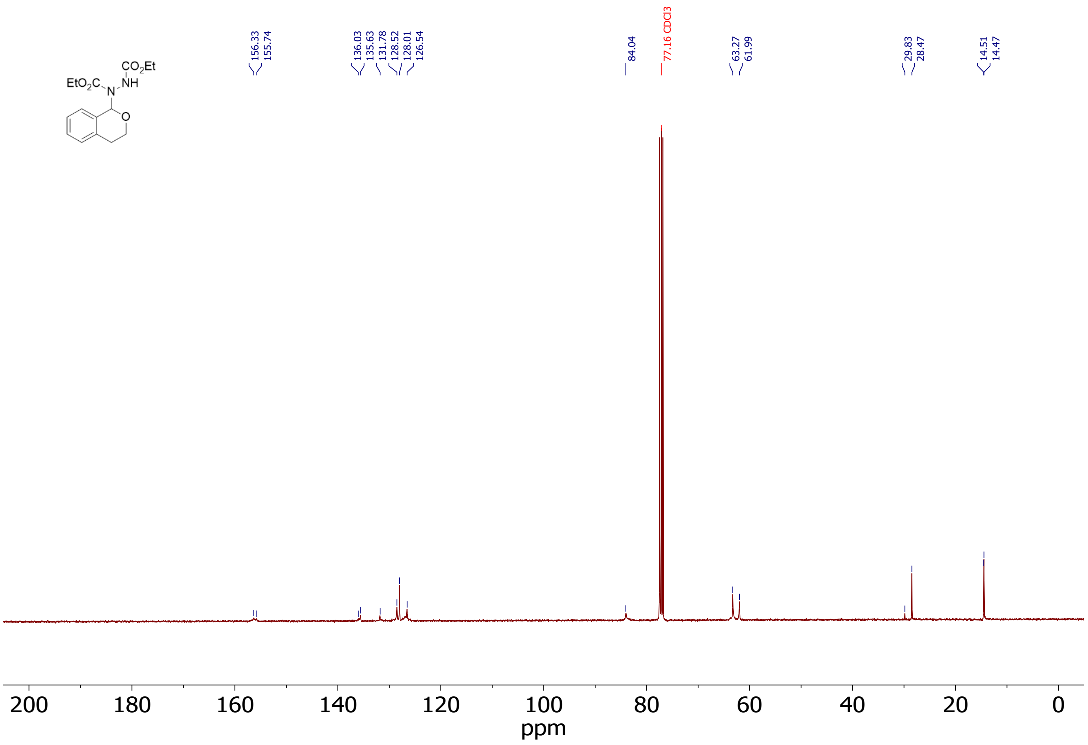

Figure S28. $^{13}C\{^{1}H\}$ NMR spectrum of **3d** in $CDCl_3$ (101 MHz).

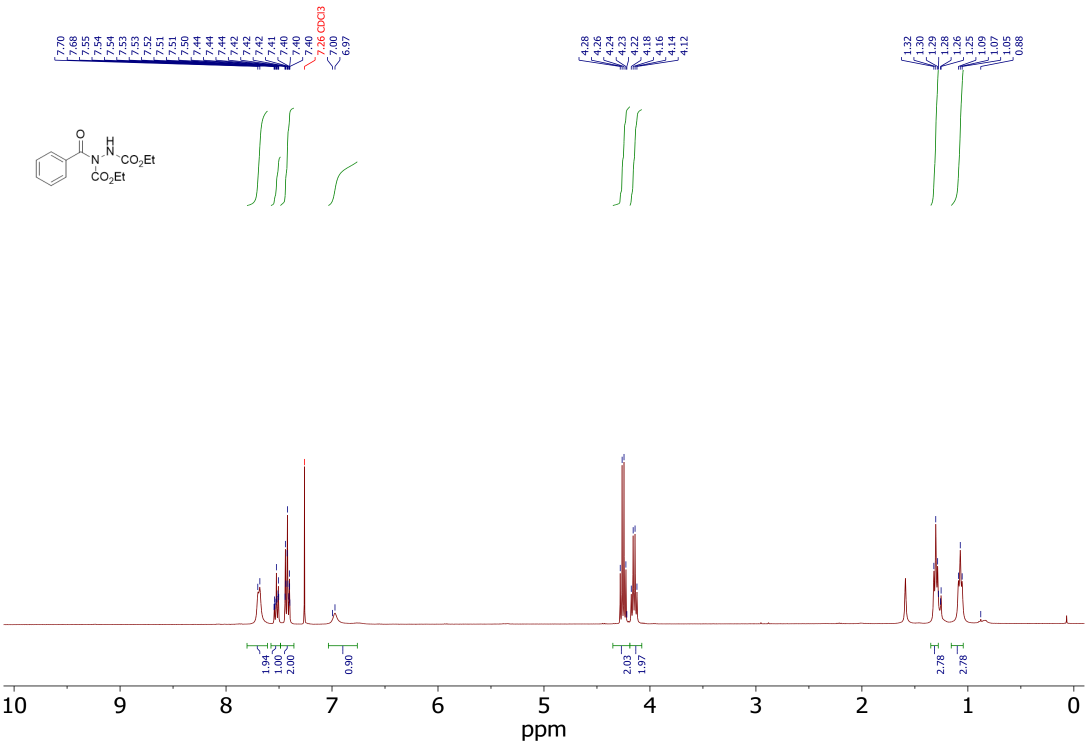

Figure S29. ^{1}H NMR spectrum of **3e** in $CDCl_3$ (400 MHz).

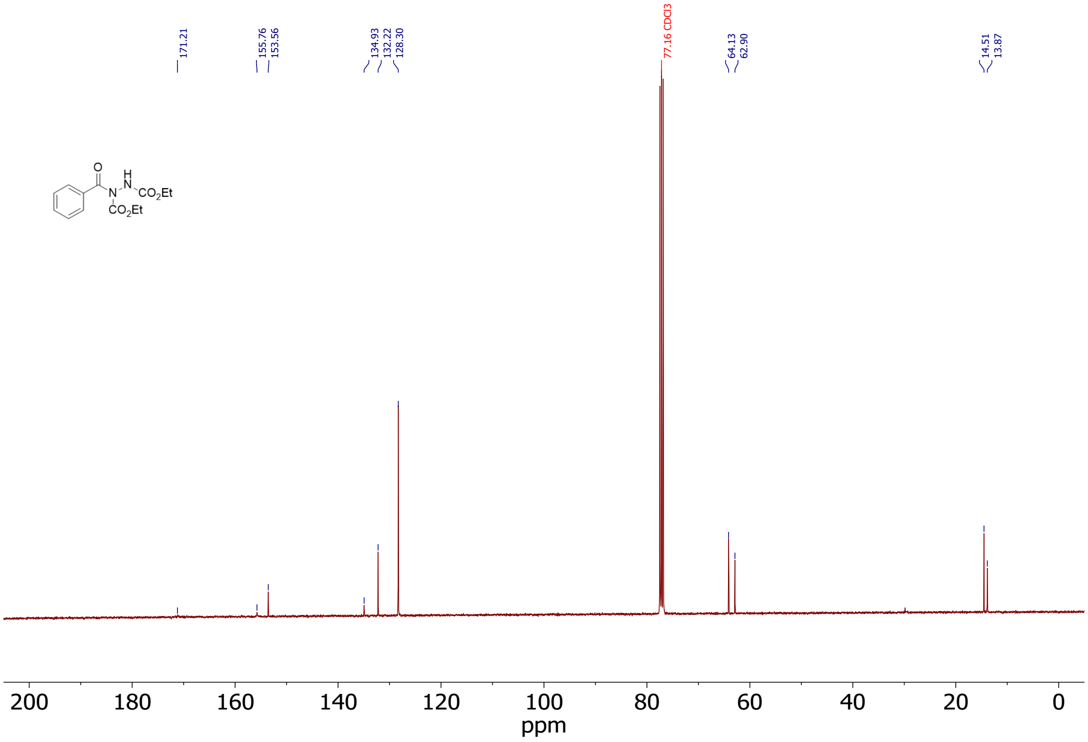

Figure S30. $^{13}C\{^{1}H\}$ NMR spectrum of **3e** in $CDCl_3$ (101 MHz).

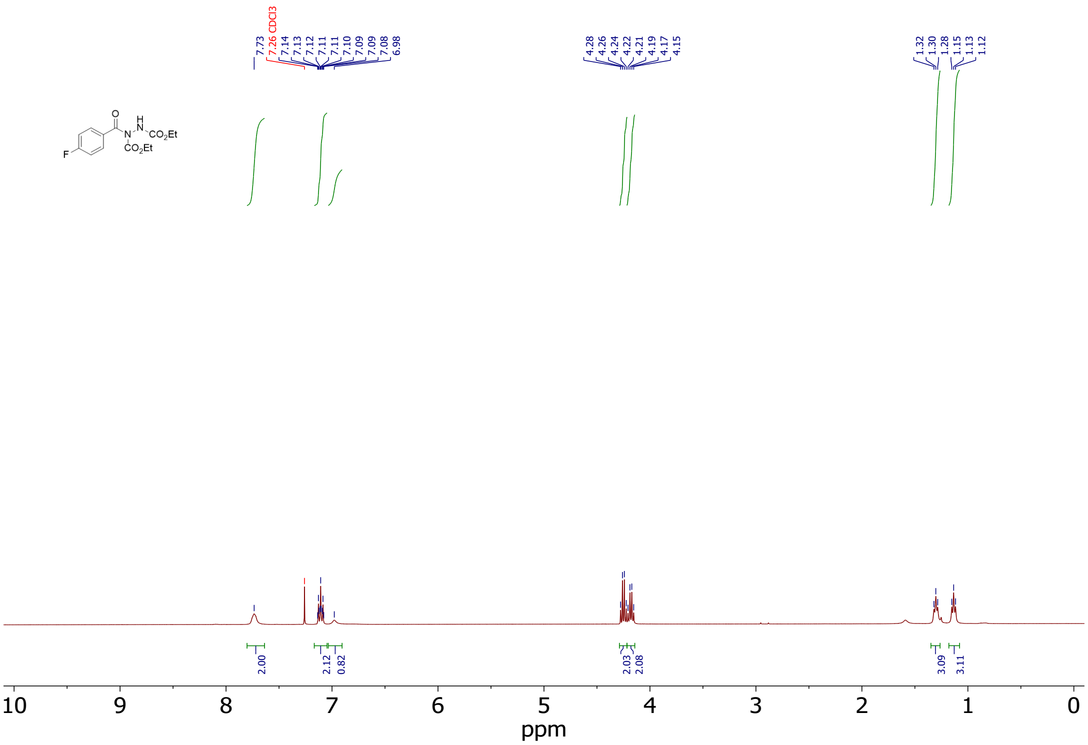

Figure S31. ^{1}H NMR spectrum of **3f** in $CDCl_3$ (400 MHz).

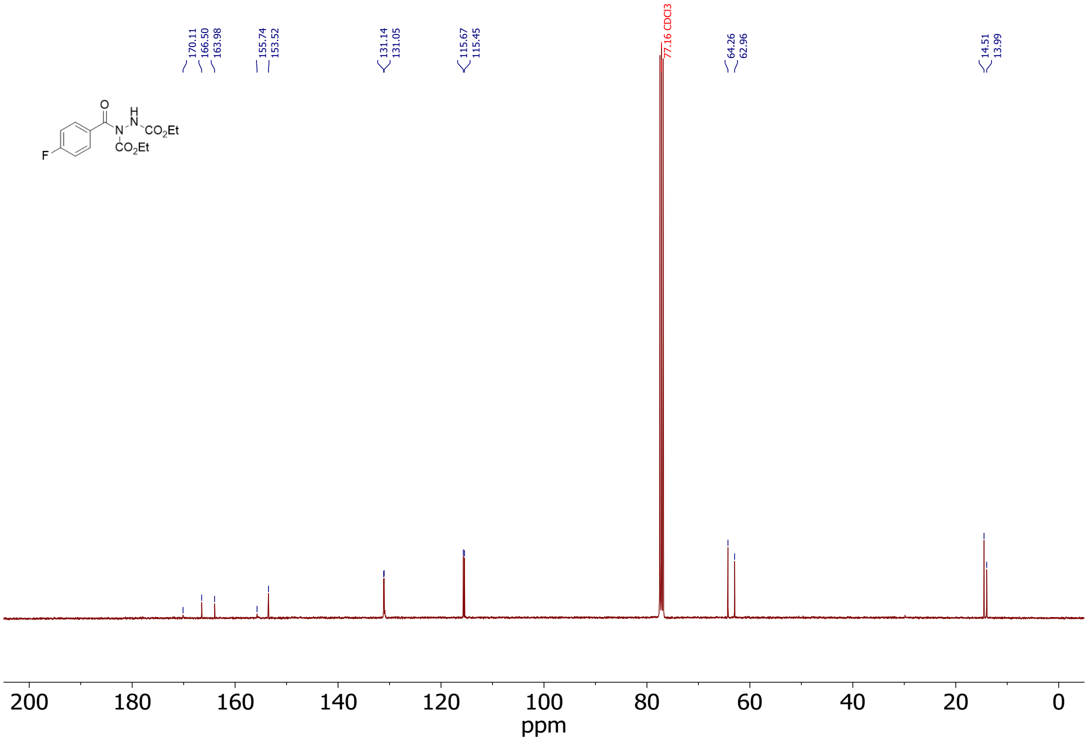

Figure S32. ^{13}C{^{1}H} NMR spectrum of **3f** in $CDCl_3$ (101 MHz).

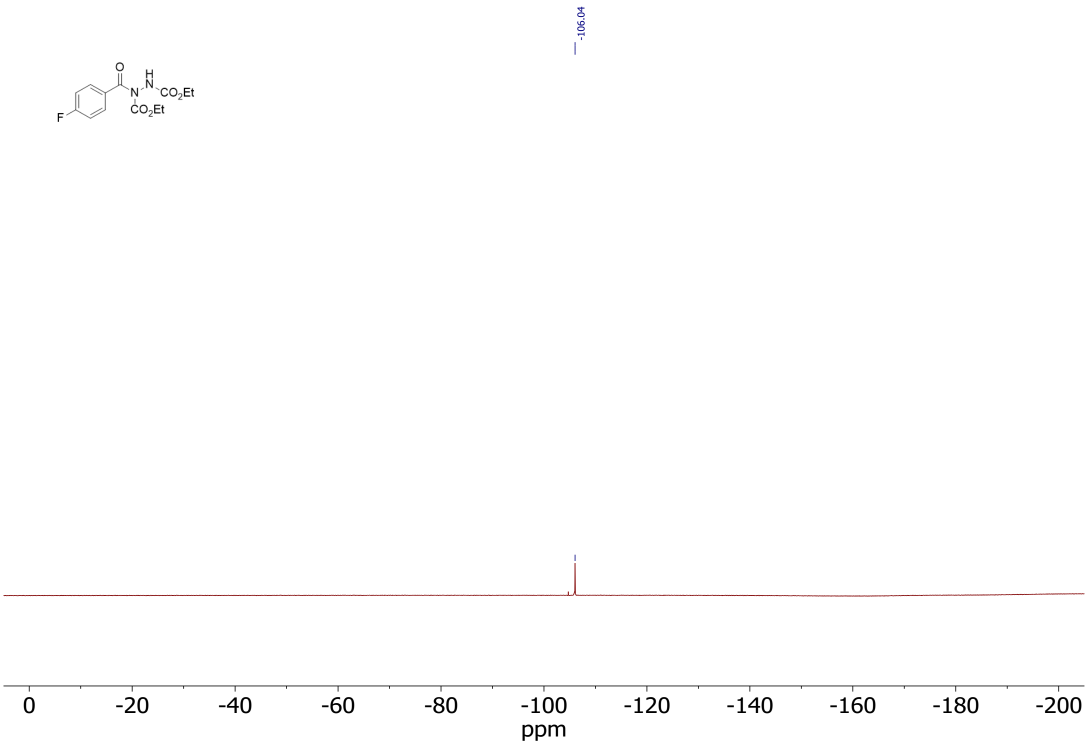

Figure S33. $^{19}F\{^{1}H\}$ NMR spectrum of **3f** in $CDCl_3$ (376 MHz).

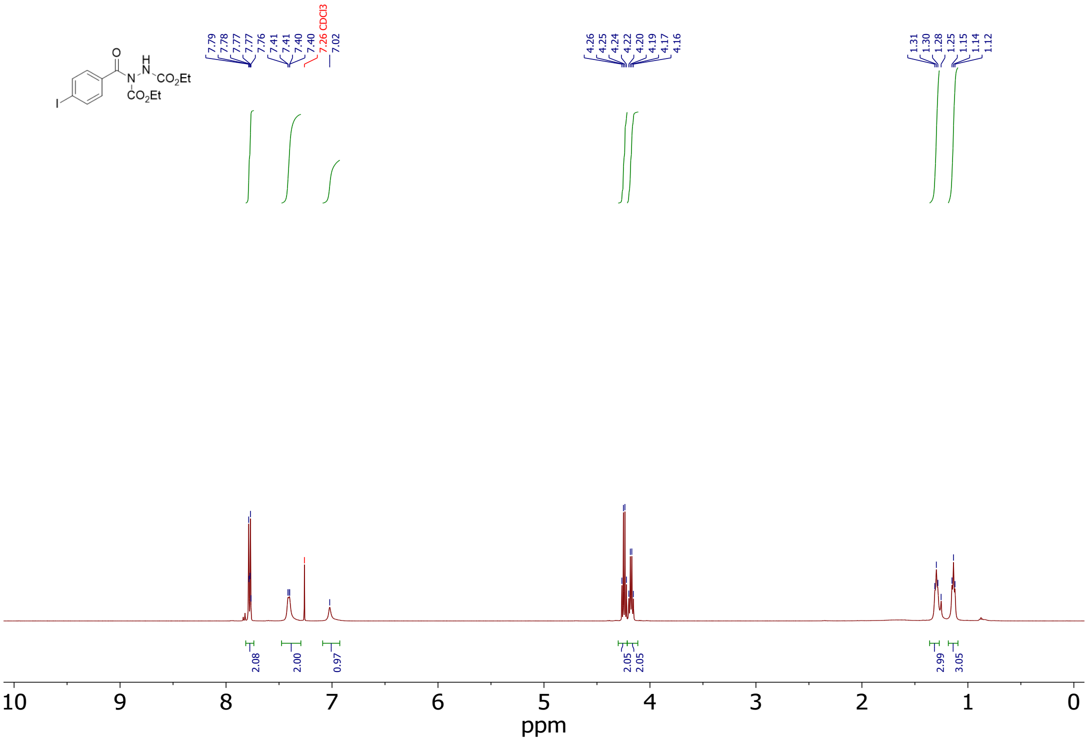

Figure S34. ^{1}H NMR spectrum of **3g** in $CDCl_3$ (400 MHz).

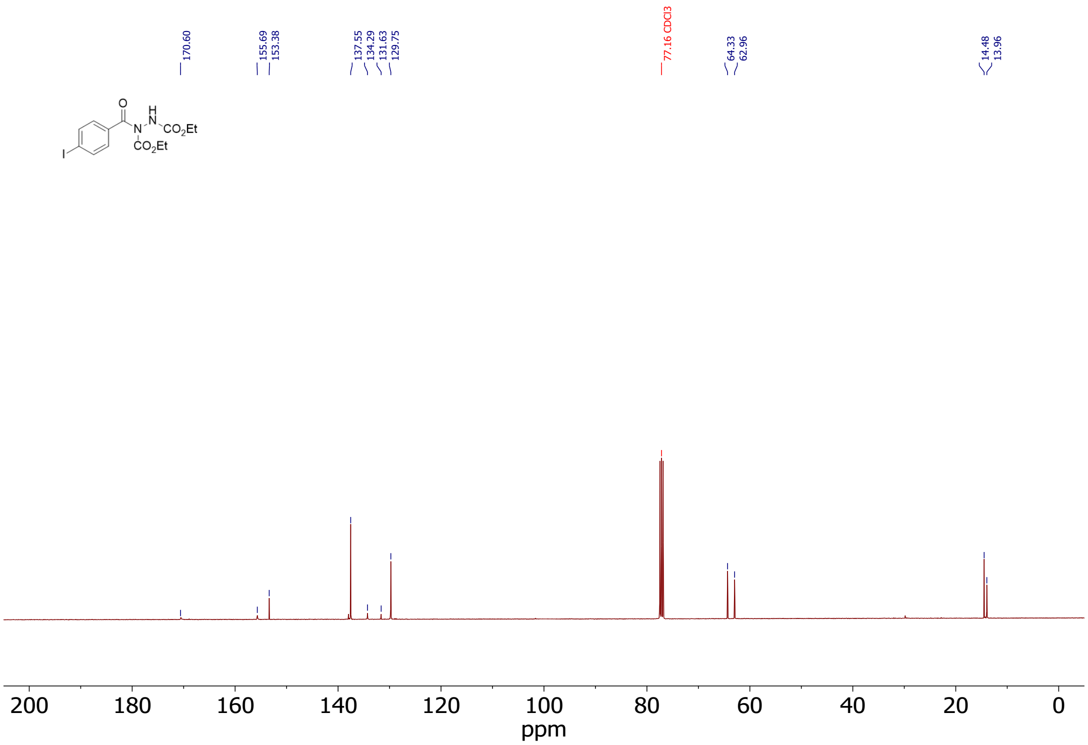

Figure S35. $^{13}C\{^{1}H\}$ NMR spectrum of **3g** in $CDCl_3$ (101 MHz).

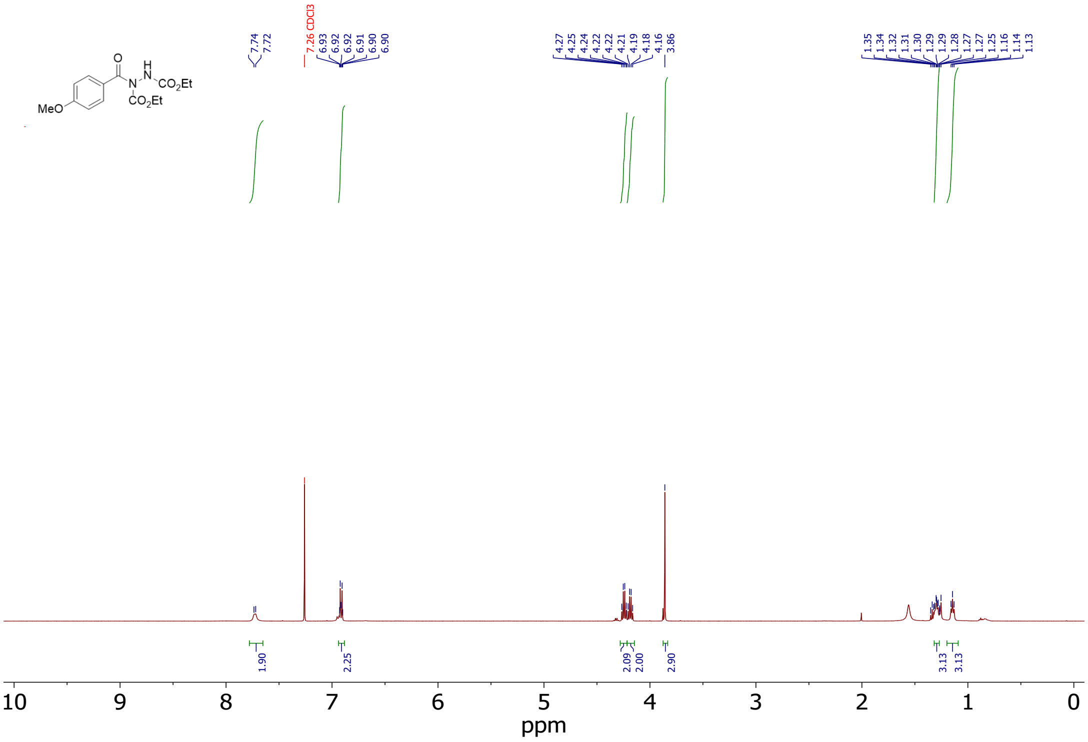

Figure S36. ^{1}H NMR spectrum of **3h** in $CDCl_3$ (400 MHz).

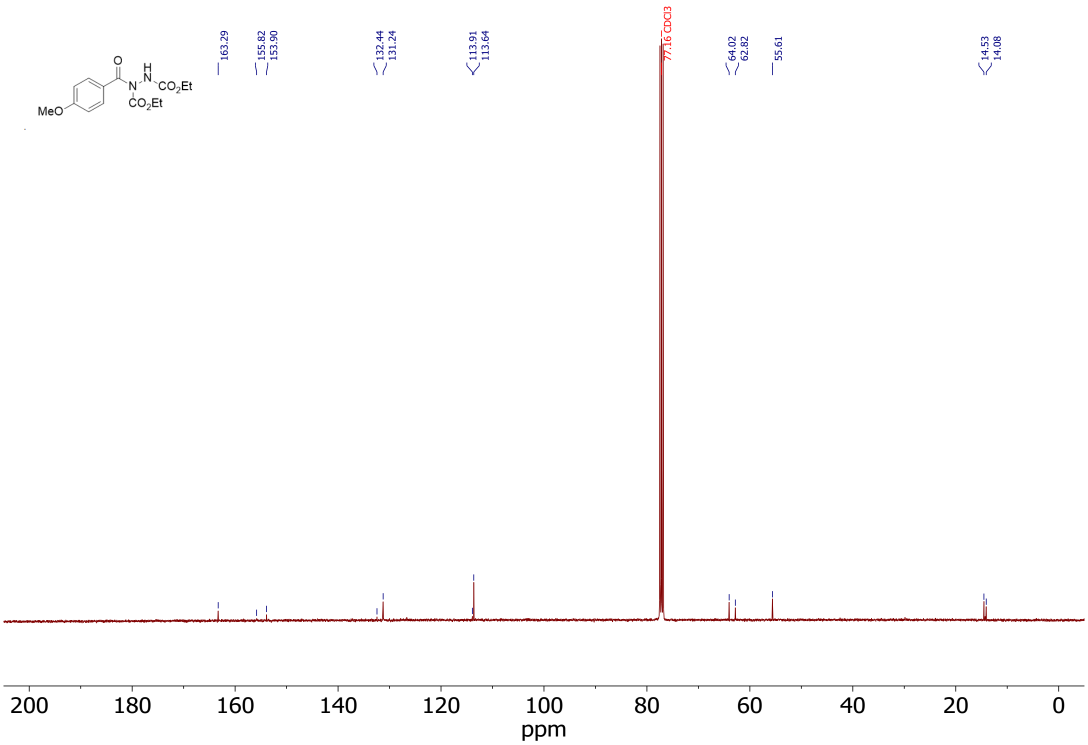

Figure S37. $^{13}C\{^{1}H\}$ NMR spectrum of **3h** in $CDCl_3$ (101 MHz).

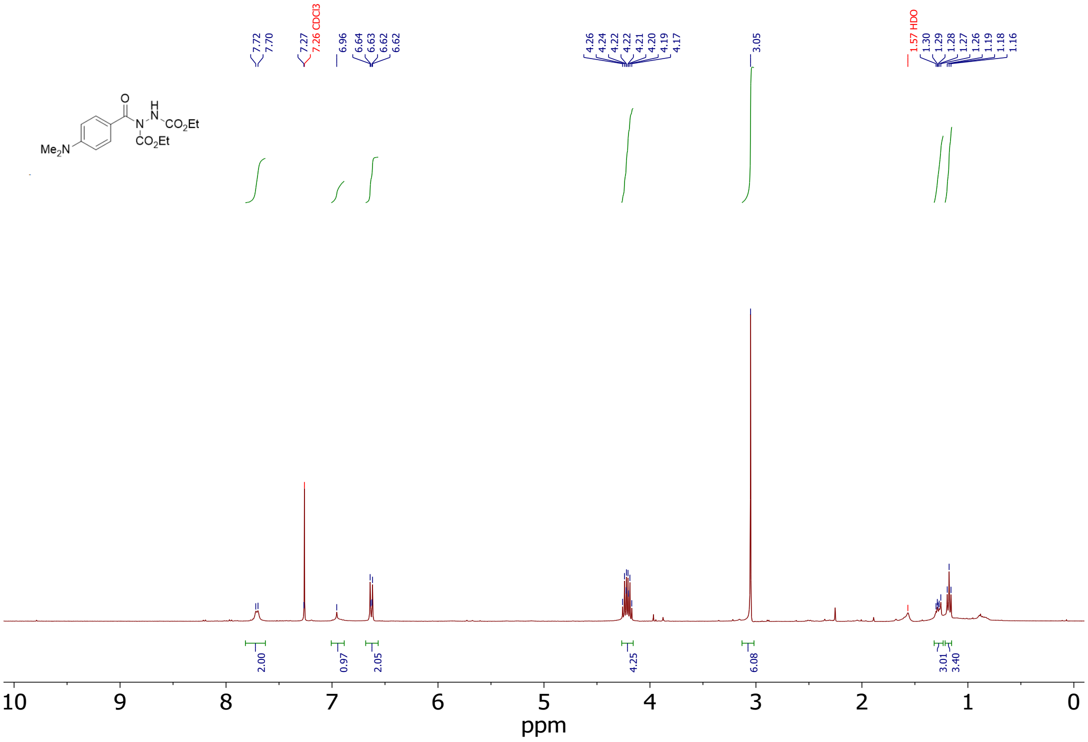

Figure S38. ^{1}H NMR spectrum of **3i** in $CDCl_3$ (400 MHz).

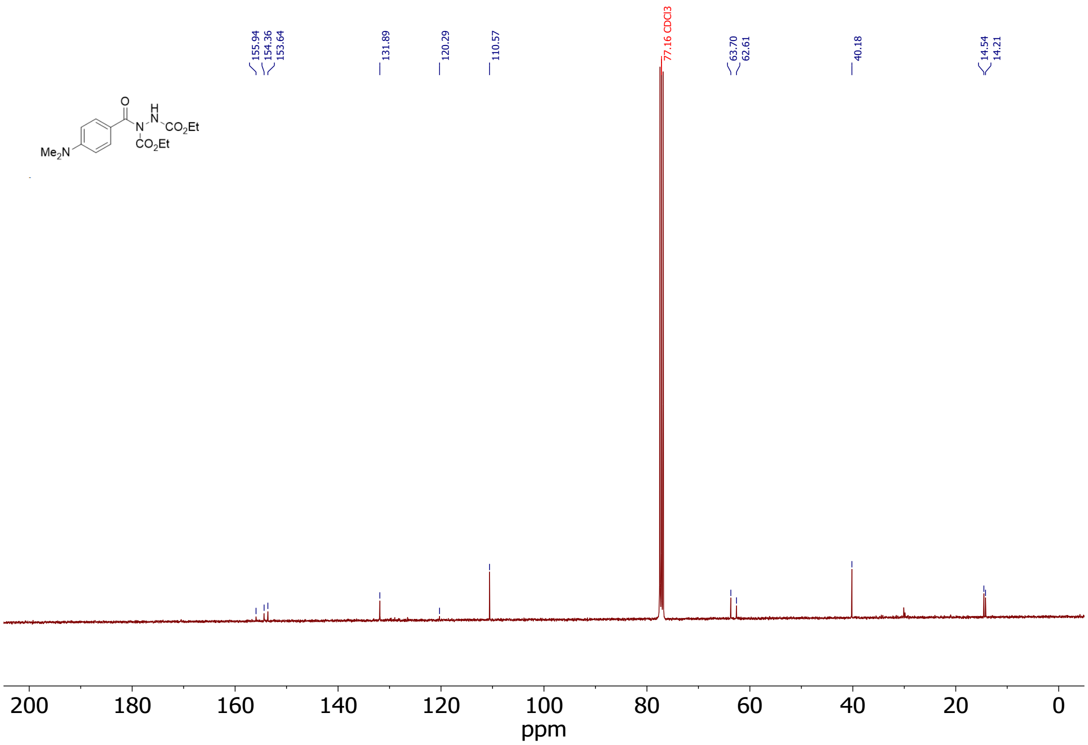

Figure S39. $^{13}C\{^{1}H\}$ NMR spectrum of **3i** in $CDCl_3$ (101 MHz).

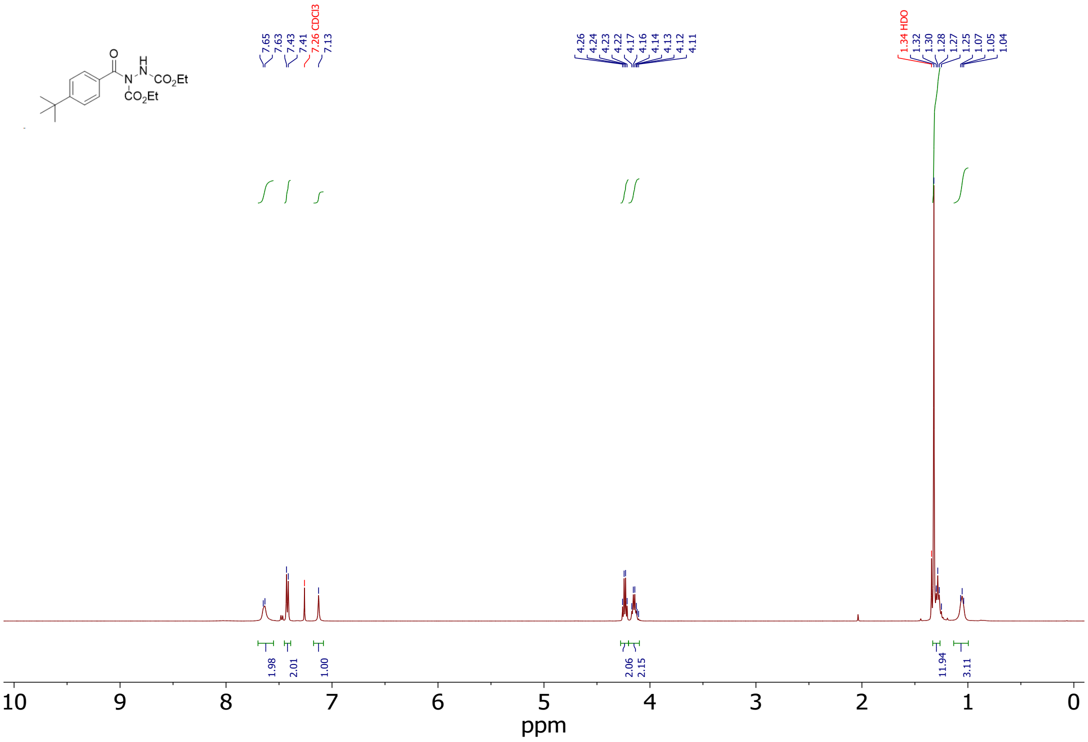

Figure S40. ^{1}H NMR spectrum of **3j** in $CDCl_3$ (400 MHz).

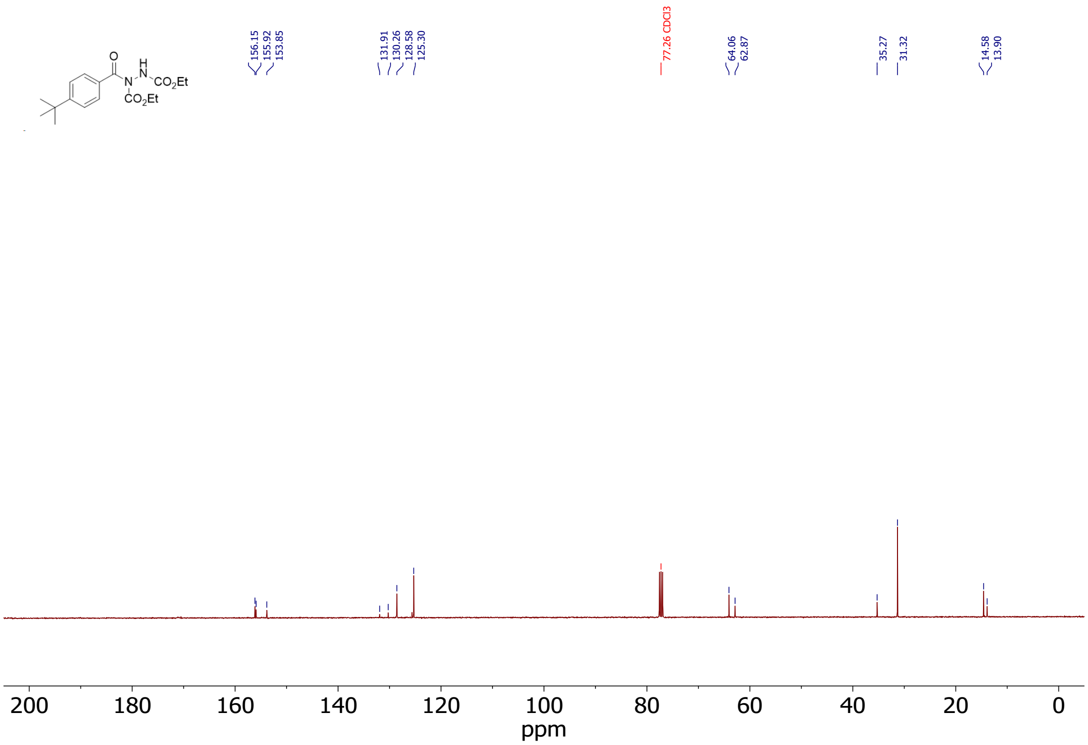

Figure S41. $^{13}C\{^{1}H\}$ NMR spectrum of **3j** in $CDCl_3$ (101 MHz).

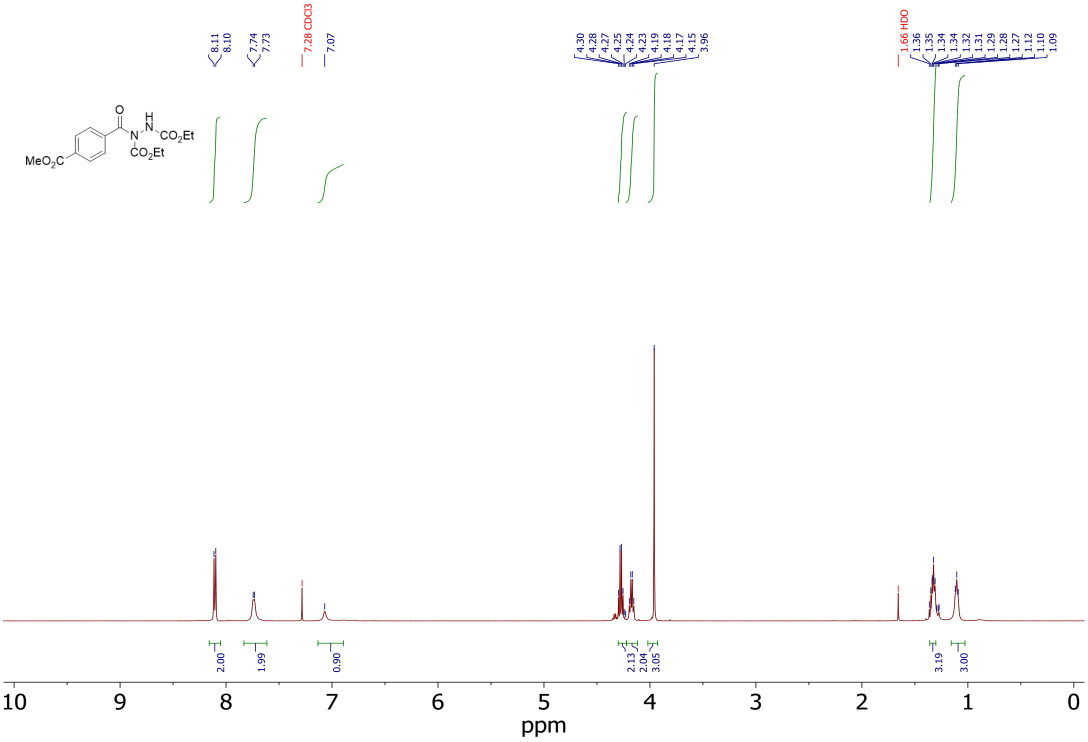

Figure S42. ^{1}H NMR spectrum of **3k** in $CDCl_3$ (400 MHz).

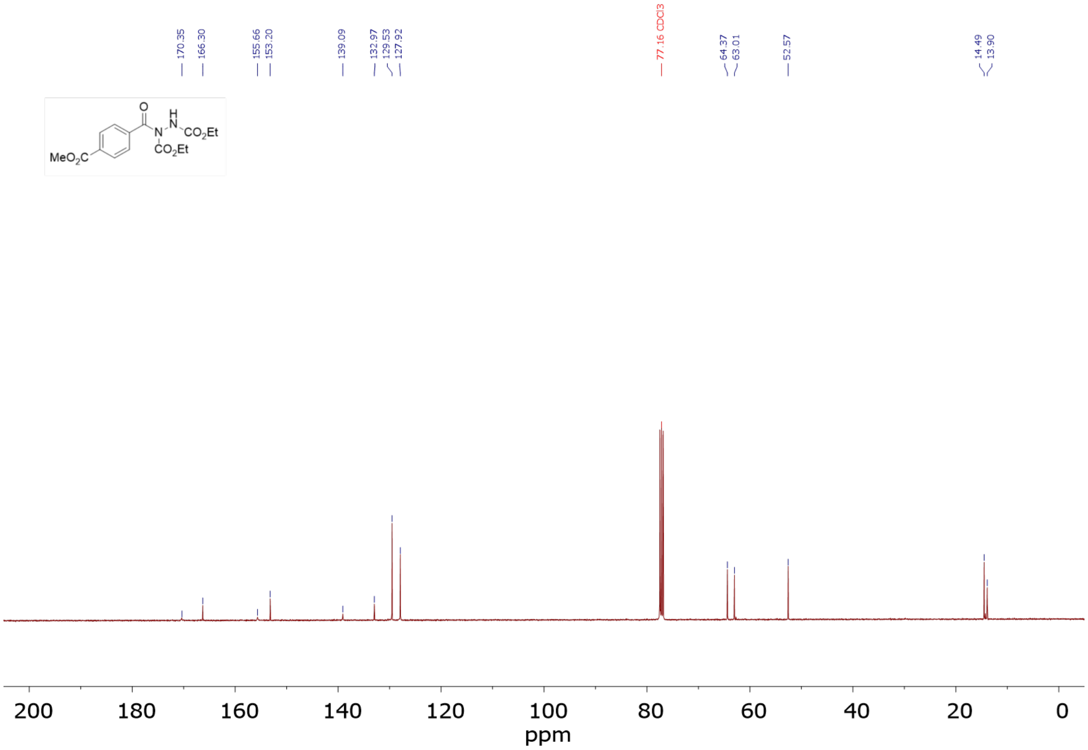

Figure S43. $^{13}C\{^{1}H\}$ NMR spectrum of **3k** in $CDCl_3$ (101 MHz).

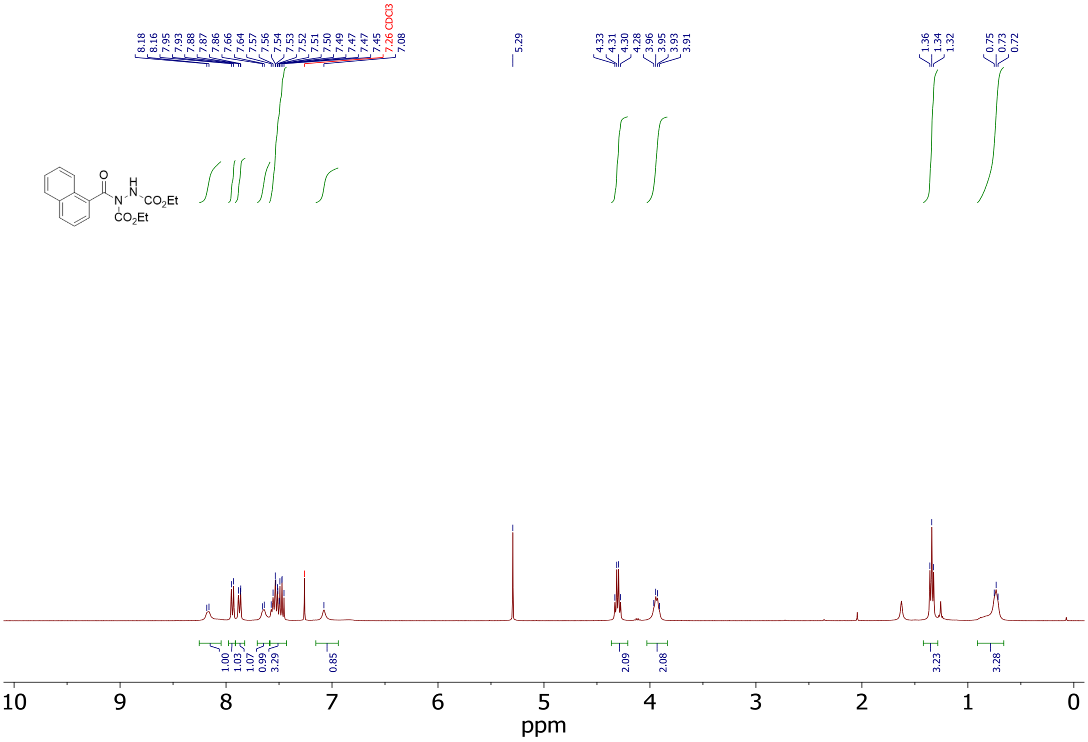

Figure S44. ^{1}H NMR spectrum of **3l** in $CDCl_3$ (400 MHz).

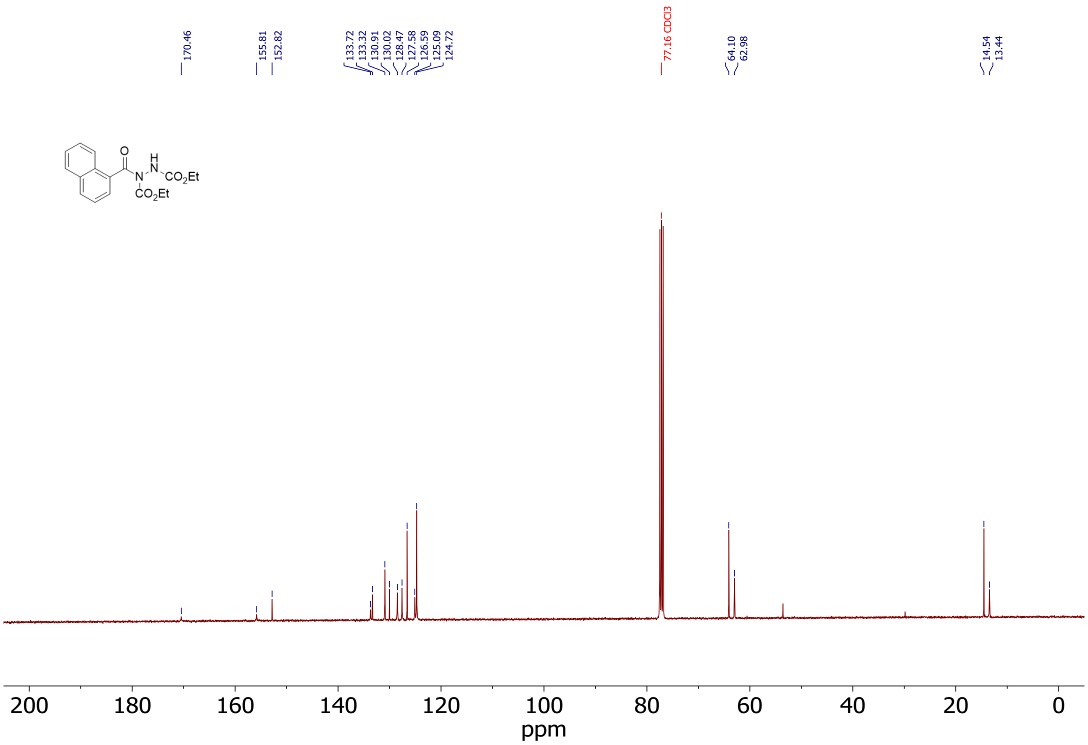

Figure S45. $^{13}C\{^{1}H\}$ NMR spectrum of **3l** in $CDCl_3$ (101 MHz).

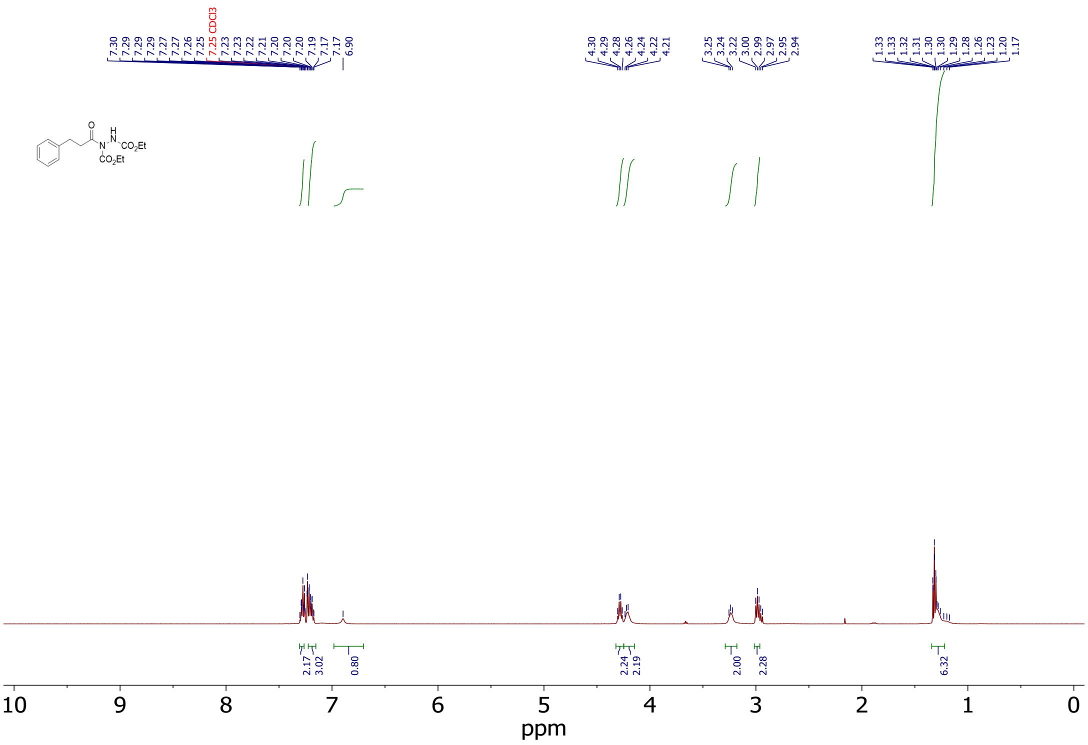

Figure S46. ^{1}H NMR spectrum of **3m** in $CDCl_3$ (400 MHz).

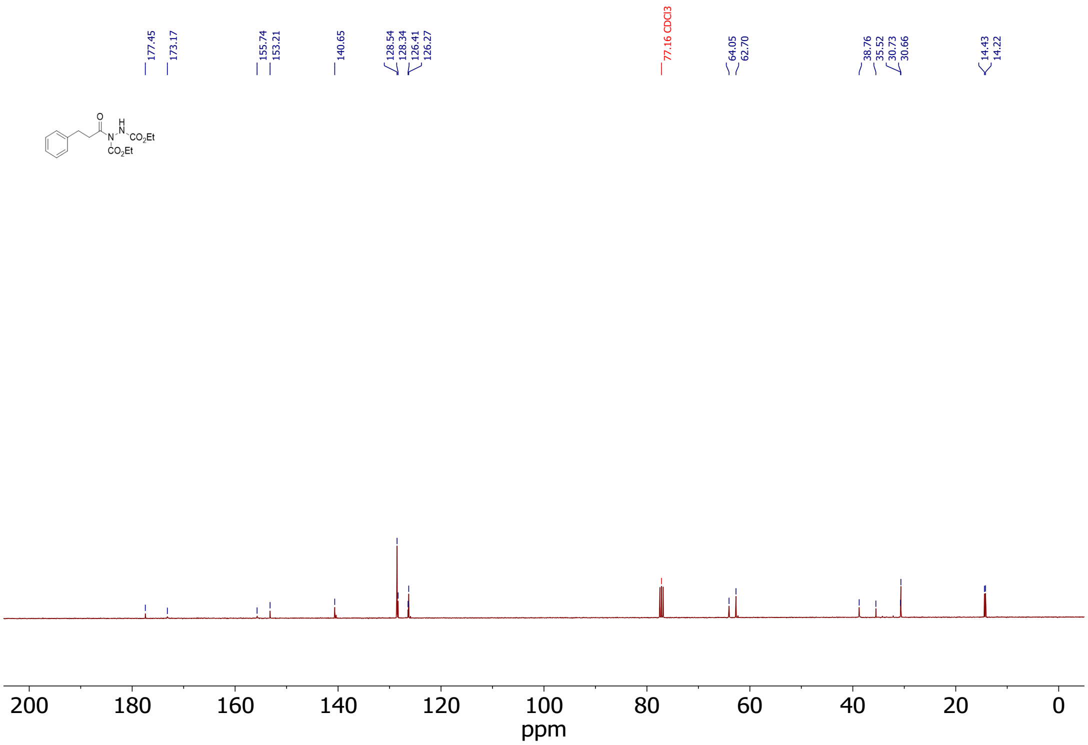

Figure S47. $^{13}C\{^{1}H\}$ NMR spectrum of **3m** in $CDCl_3$ (101 MHz).

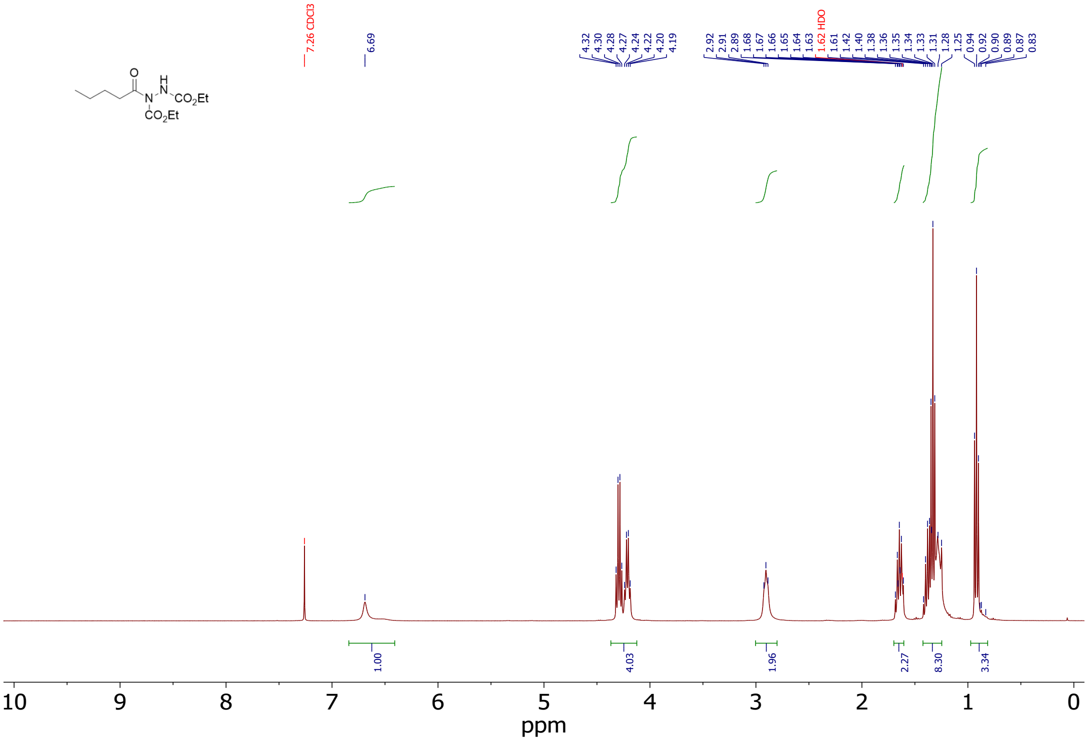

Figure S48. ^{1}H NMR spectrum of **3n** in $CDCl_3$ (400 MHz).

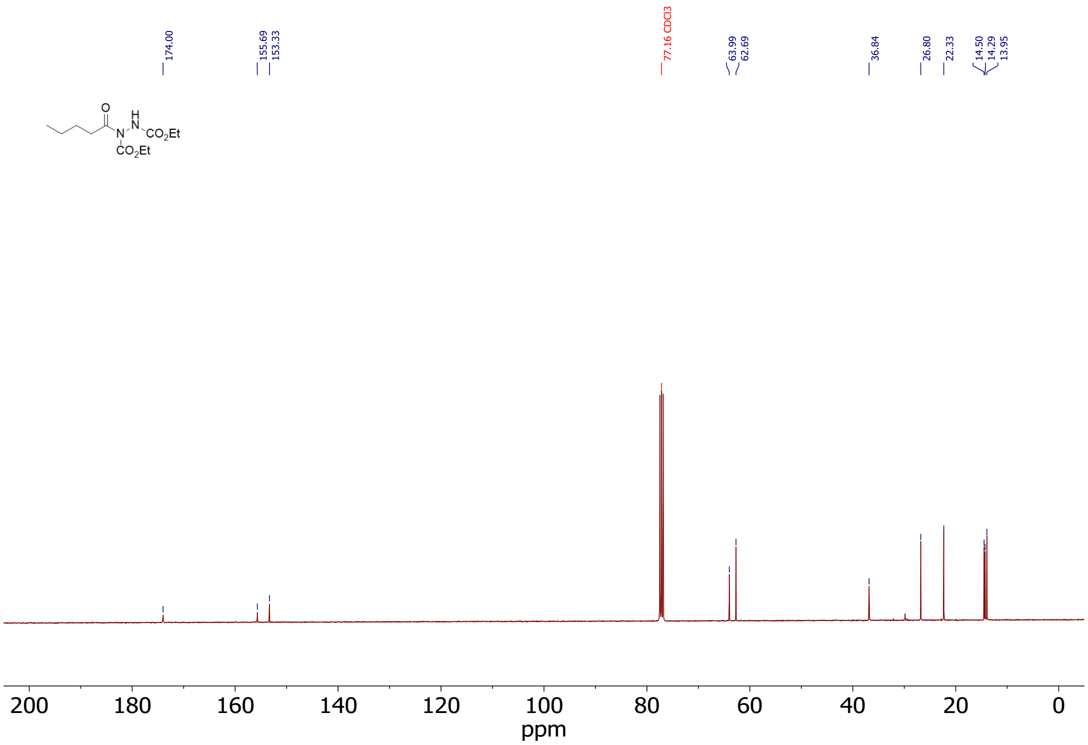

Figure S49. $^{13}C\{^1H\}$ NMR spectrum of **3n** in $CDCl_3$ (101 MHz).

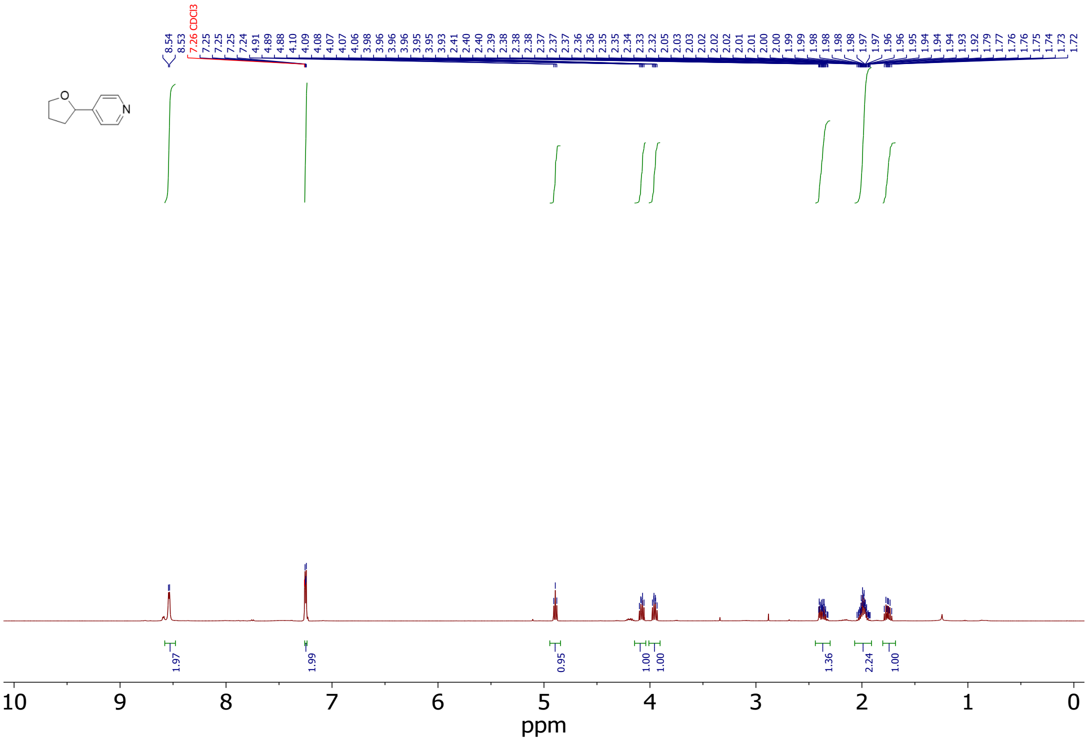

Figure S50. ^1H NMR spectrum of **6a** in $CDCl_3$ (400 MHz).

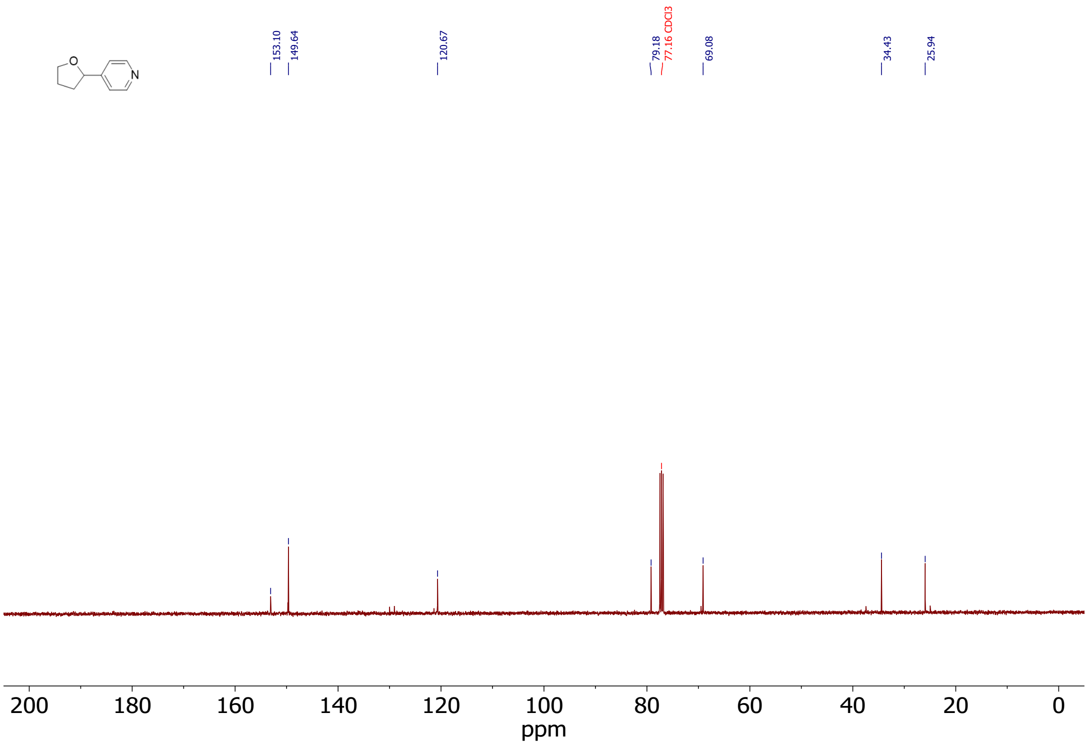

Figure S51. $^{13}C\{^{1}H\}$ NMR spectrum of **6a** in $CDCl_3$ (101 MHz).

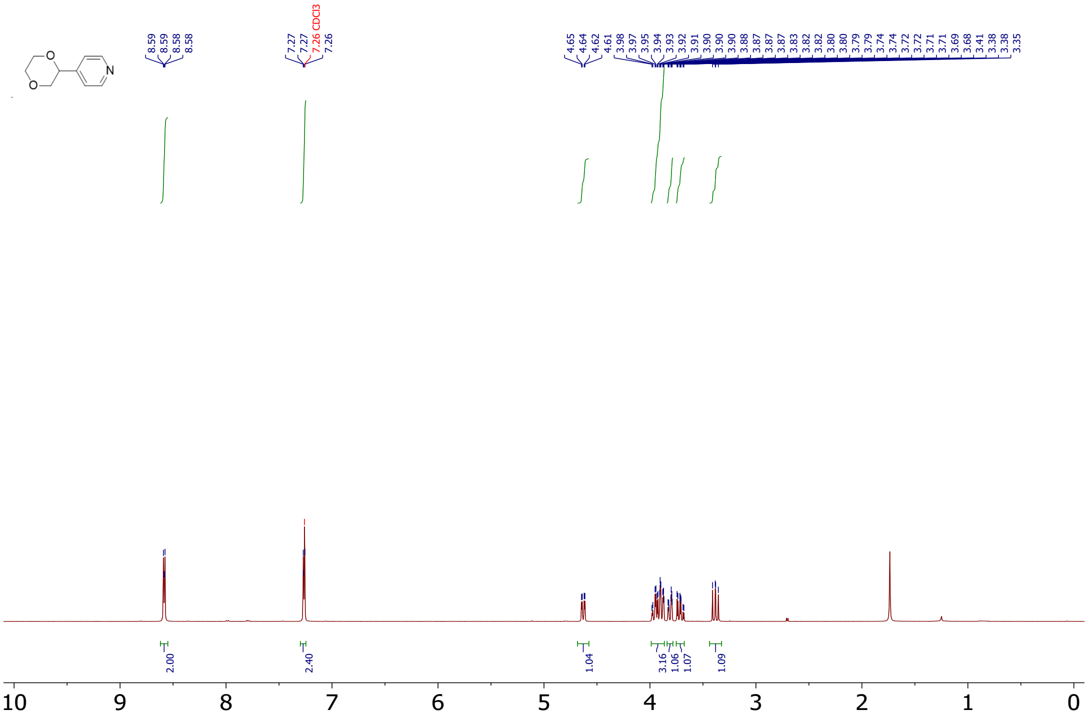

Figure S52. ^{1}H NMR spectrum of **6b** in $CDCl_3$ (400 MHz).

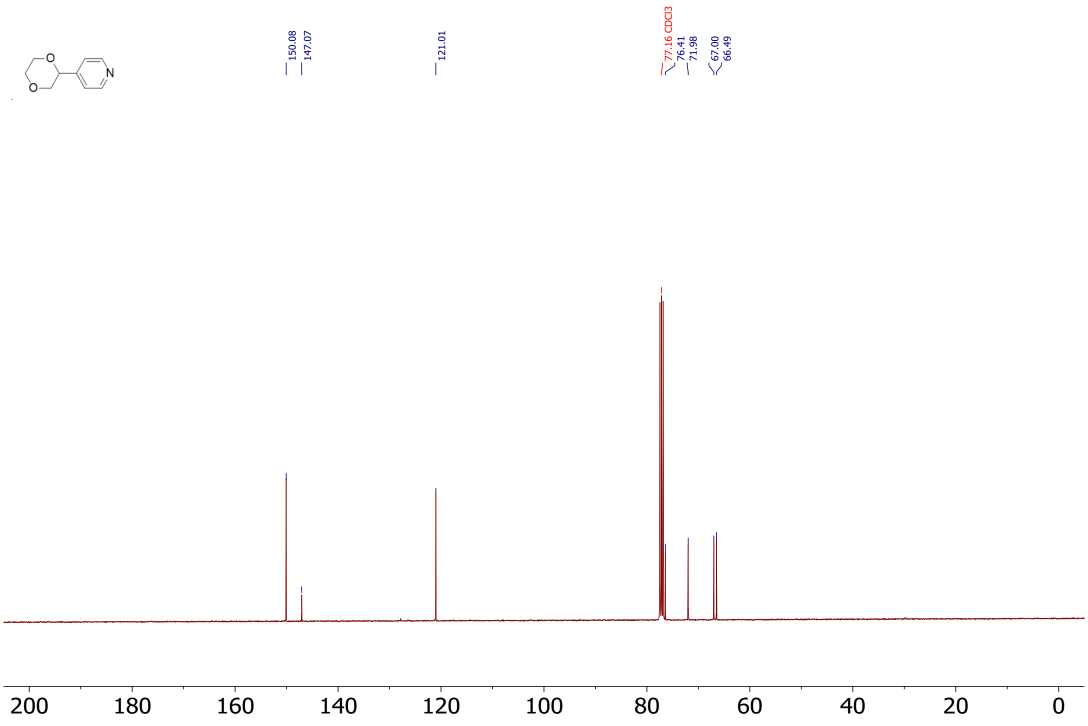

Figure S53. $^{13}C\{^{1}H\}$ NMR spectrum of **6b** in $CDCl_3$ (101 MHz).

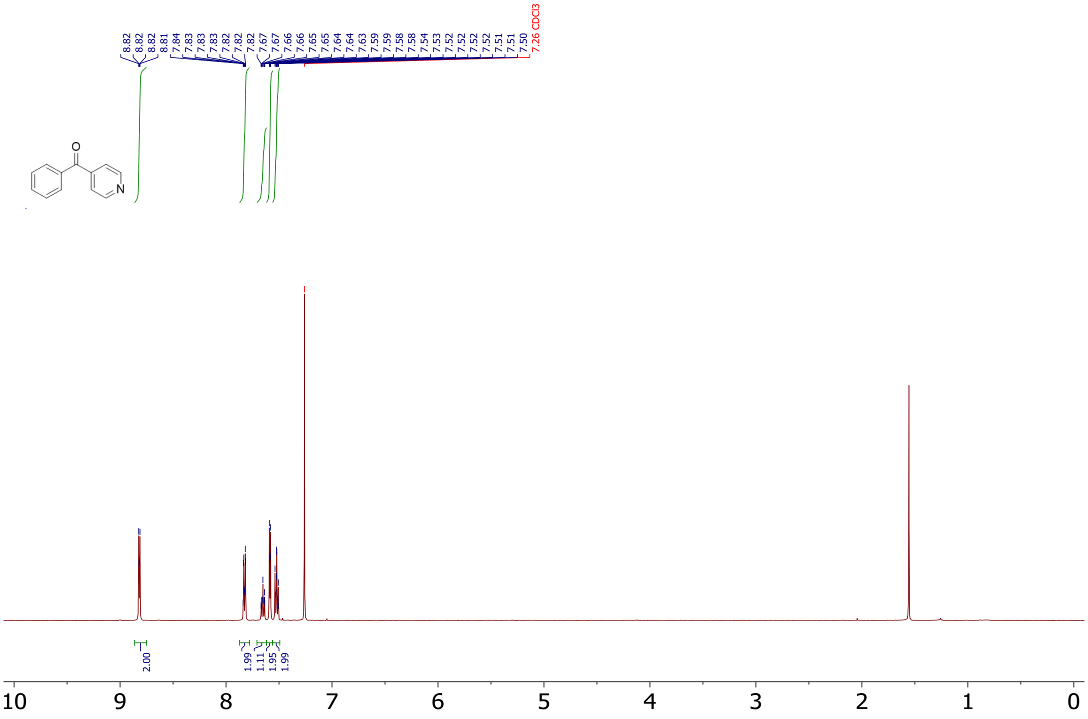

Figure S54. ^{1}H NMR spectrum of **6c** in $CDCl_3$ (400 MHz).

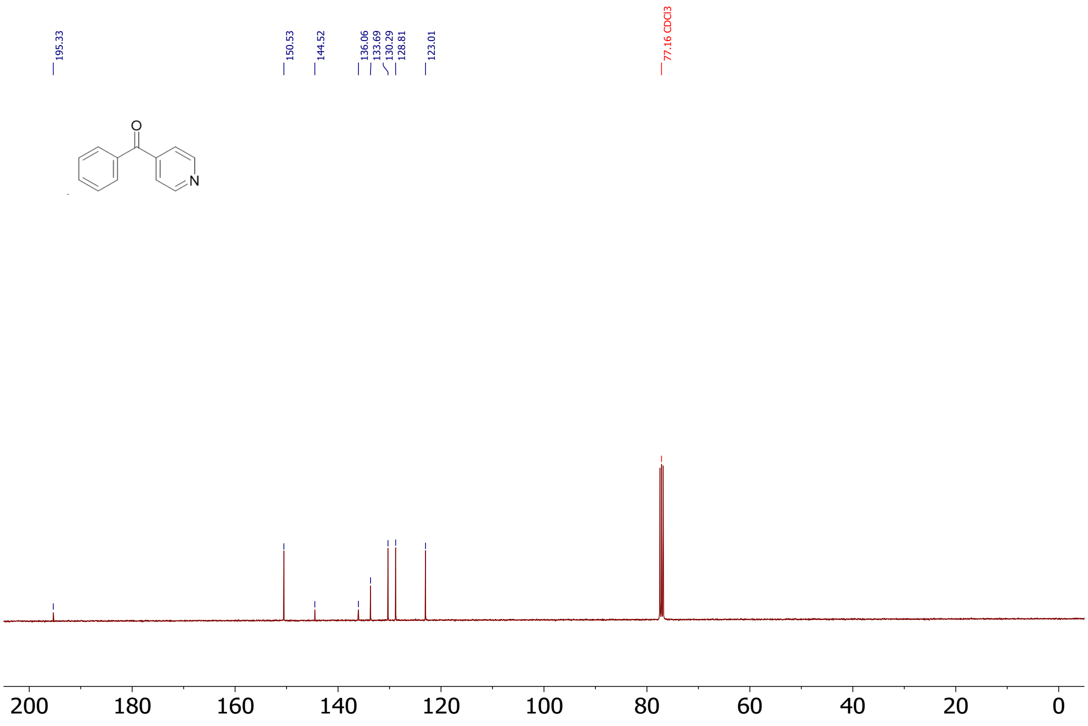

Figure S55. $^{13}C\{^{1}H\}$ NMR spectrum of **6c** in $CDCl_3$ (101 MHz).

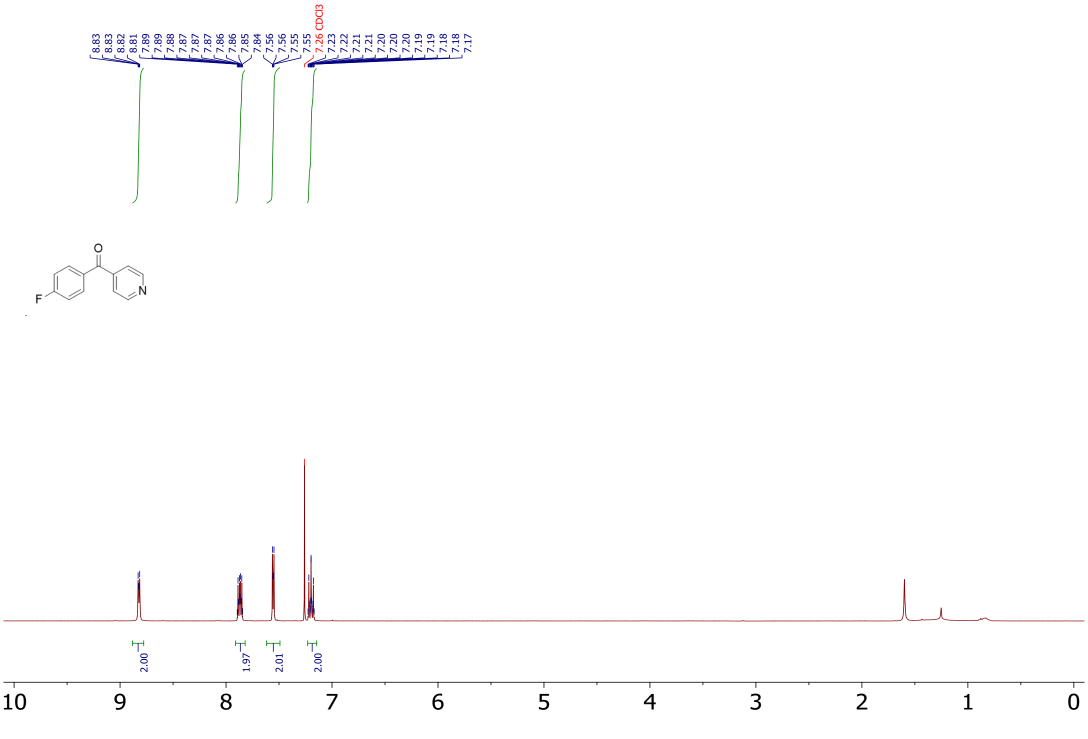

Figure S56. ^{1}H NMR spectrum of **6d** in $CDCl_3$ (400 MHz).

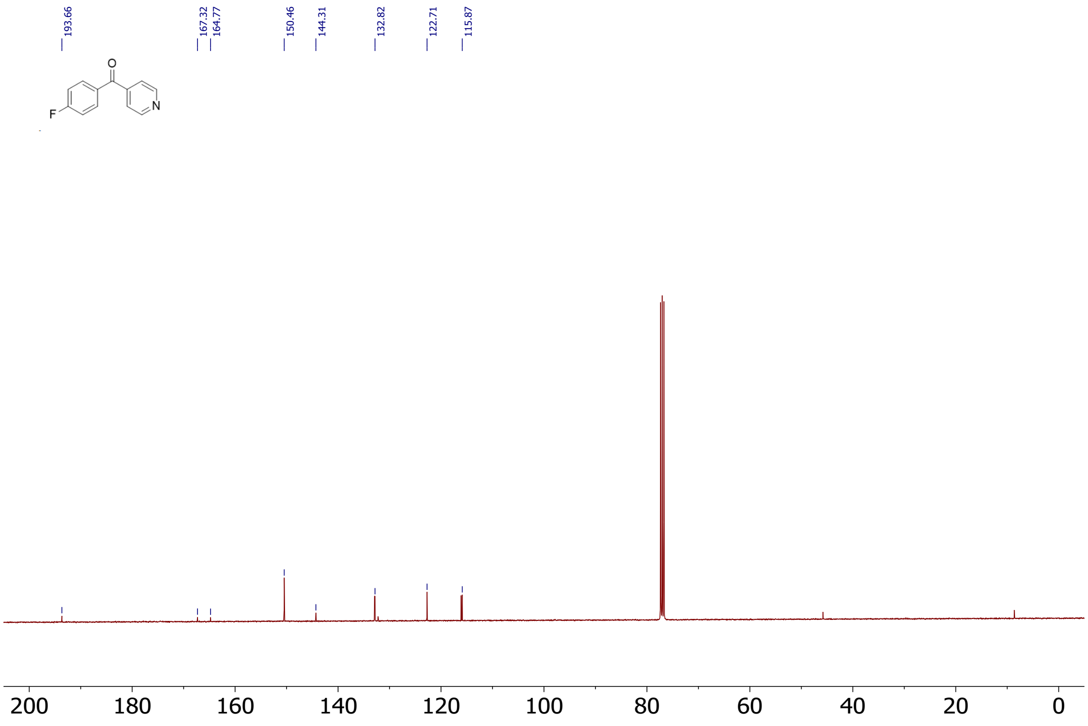

Figure S57. $^{13}C\{^{1}H\}$ NMR spectrum of **6d** in $CDCl_3$ (101 MHz).

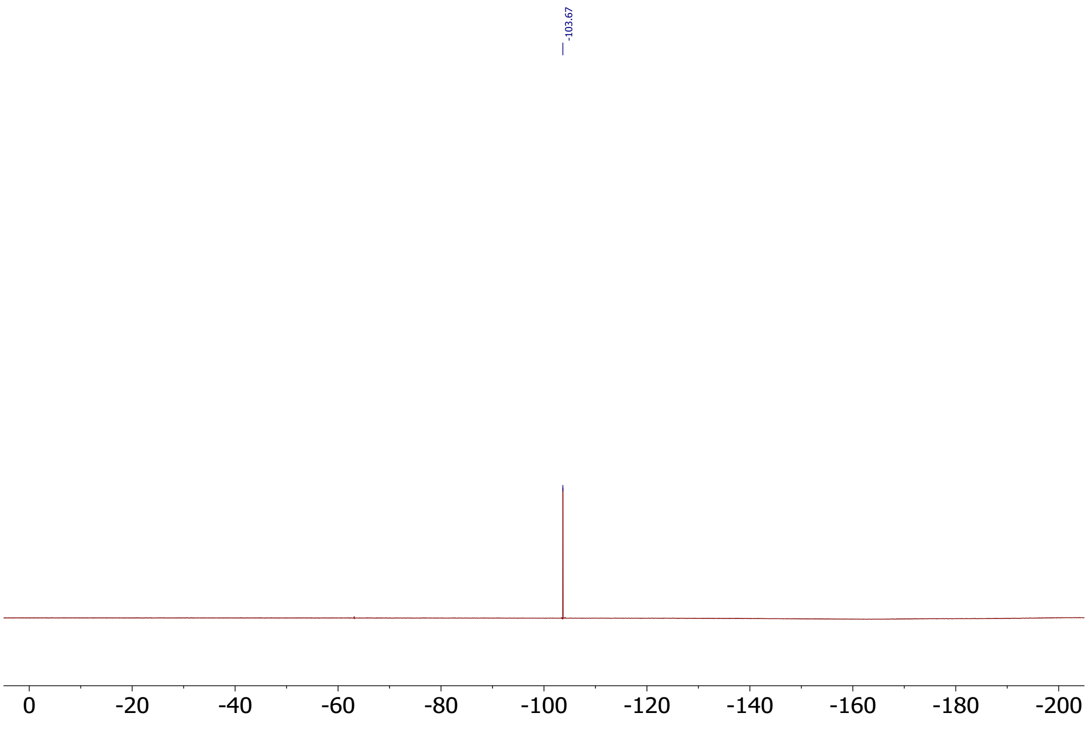

Figure S58. $^{19}F\{^{1}H\}$ NMR spectrum of **6d** in $CDCl_3$ (376 MHz).

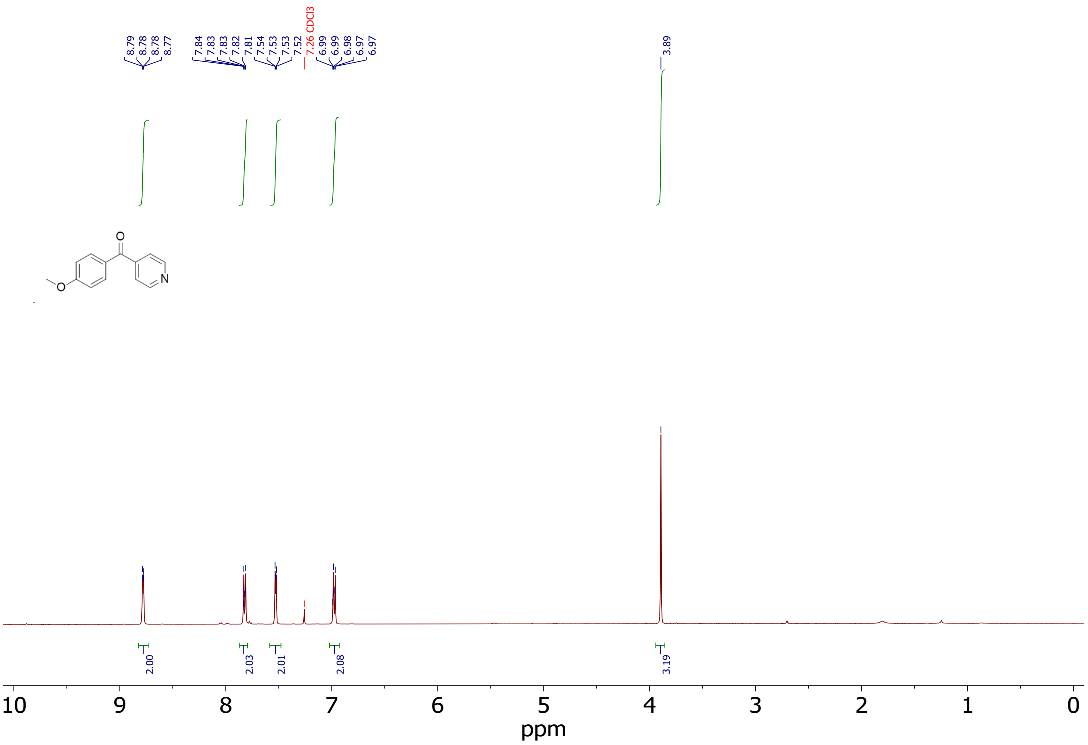

Figure S59. ^{1}H NMR spectrum of **6e** in $CDCl_3$ (400 MHz).

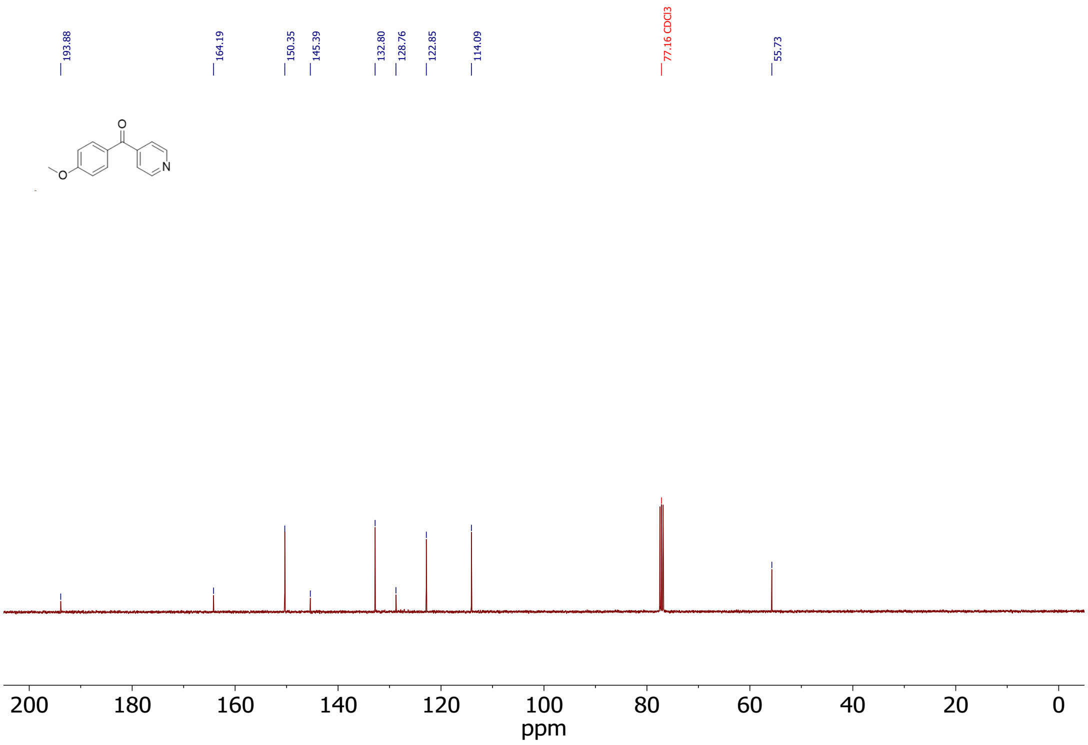

Figure S60. $^{13}C\{^{1}H\}$ NMR spectrum of **6e** in $CDCl_3$ (101 MHz).

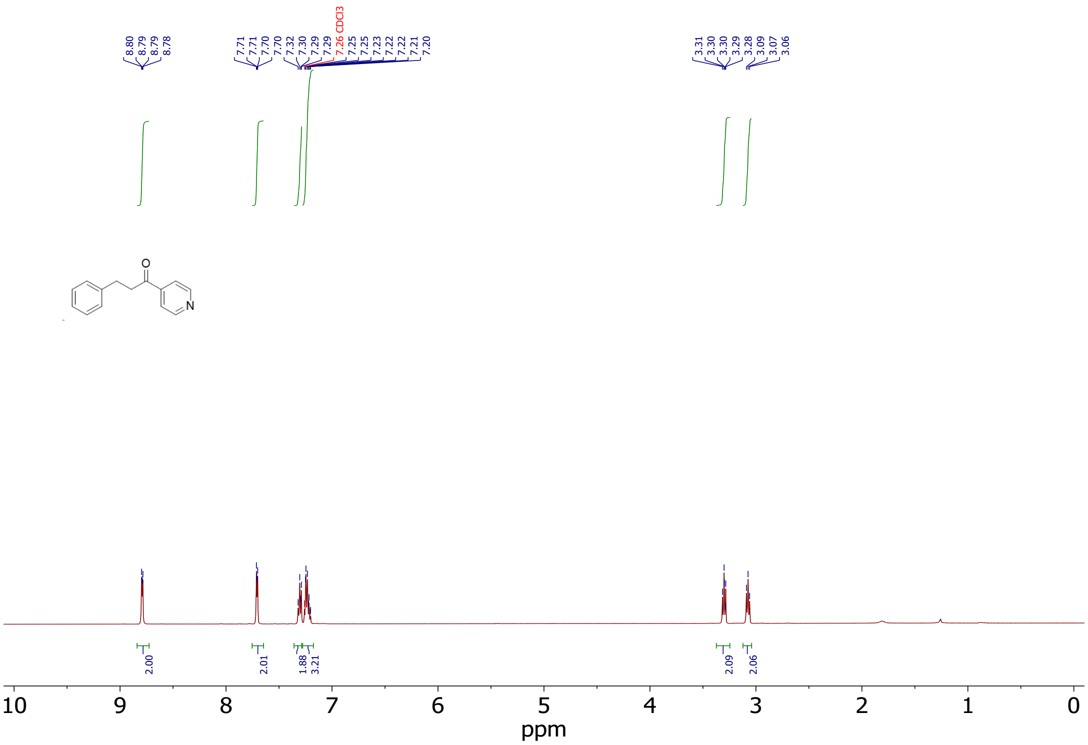

Figure S61. ^{1}H NMR spectrum of **6f** in $CDCl_3$ (400 MHz).

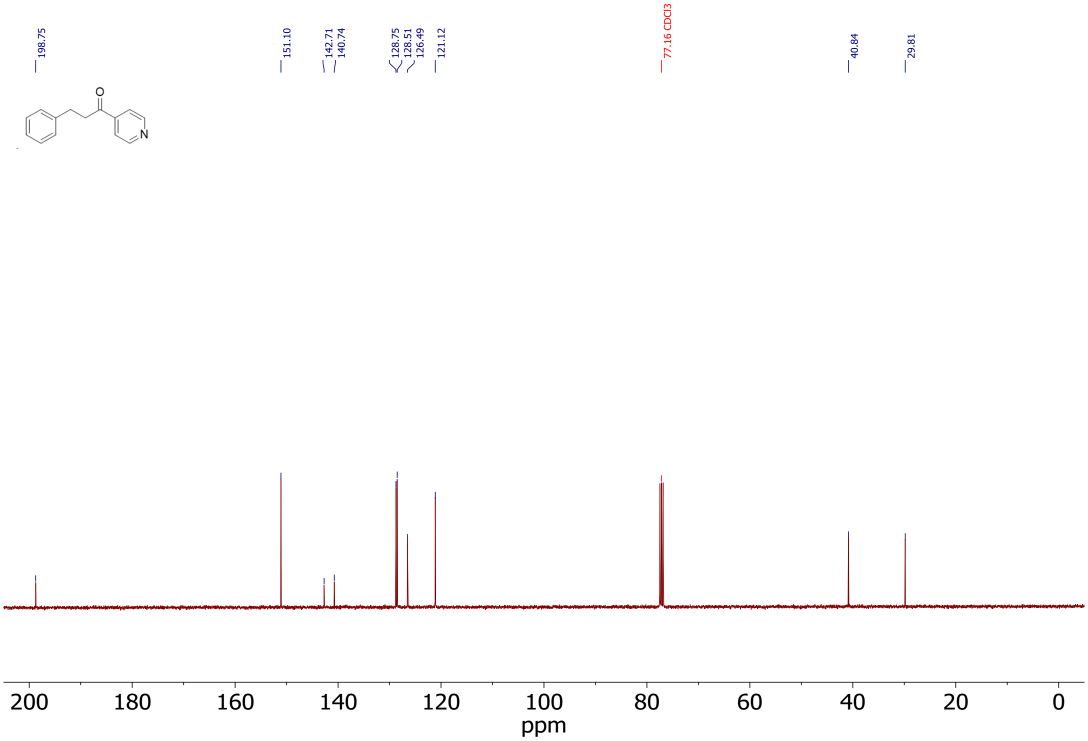

Figure S62. $^{13}C\{^{1}H\}$ NMR spectrum of **6f** in $CDCl_3$ (101 MHz).

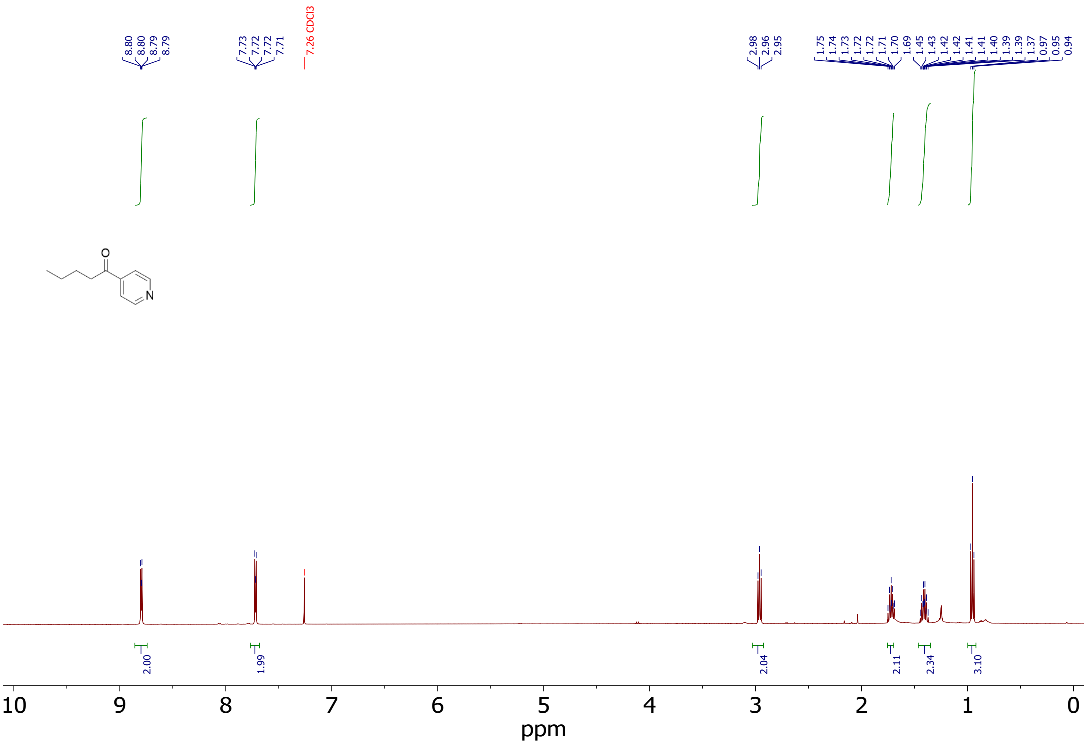

Figure S63. ^{1}H NMR spectrum of **6g** in $CDCl_3$ (400 MHz).

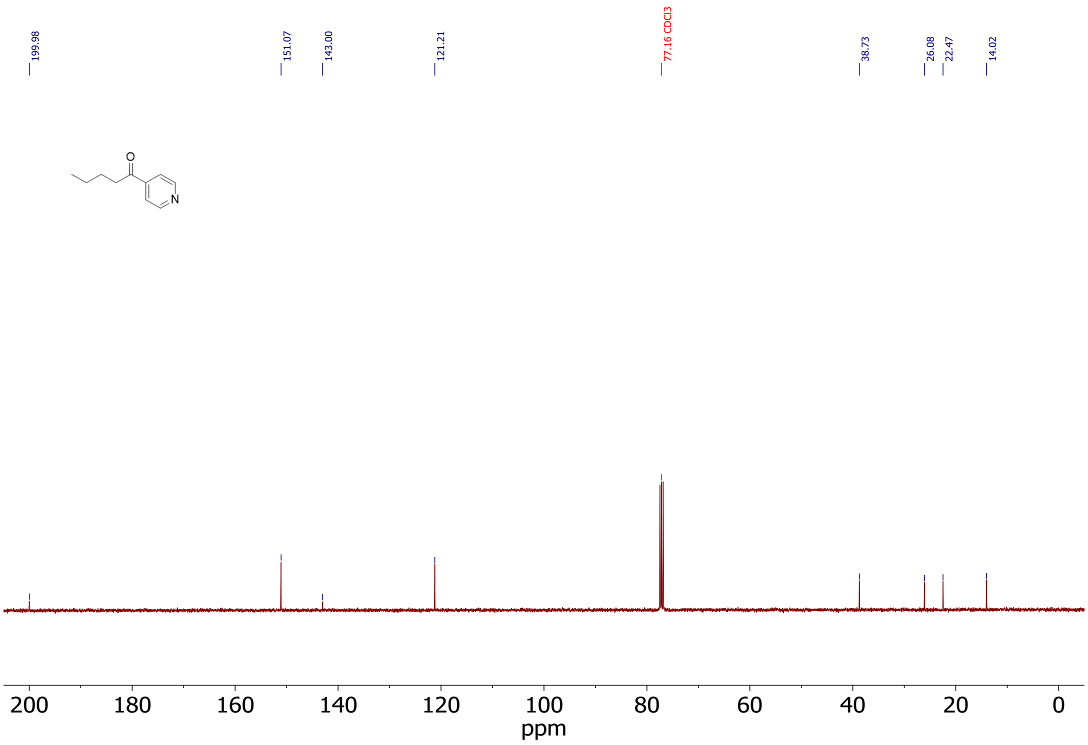

Figure S64. $^{13}C\{^{1}H\}$ NMR spectrum of **6g** in $CDCl_3$ (101 MHz).

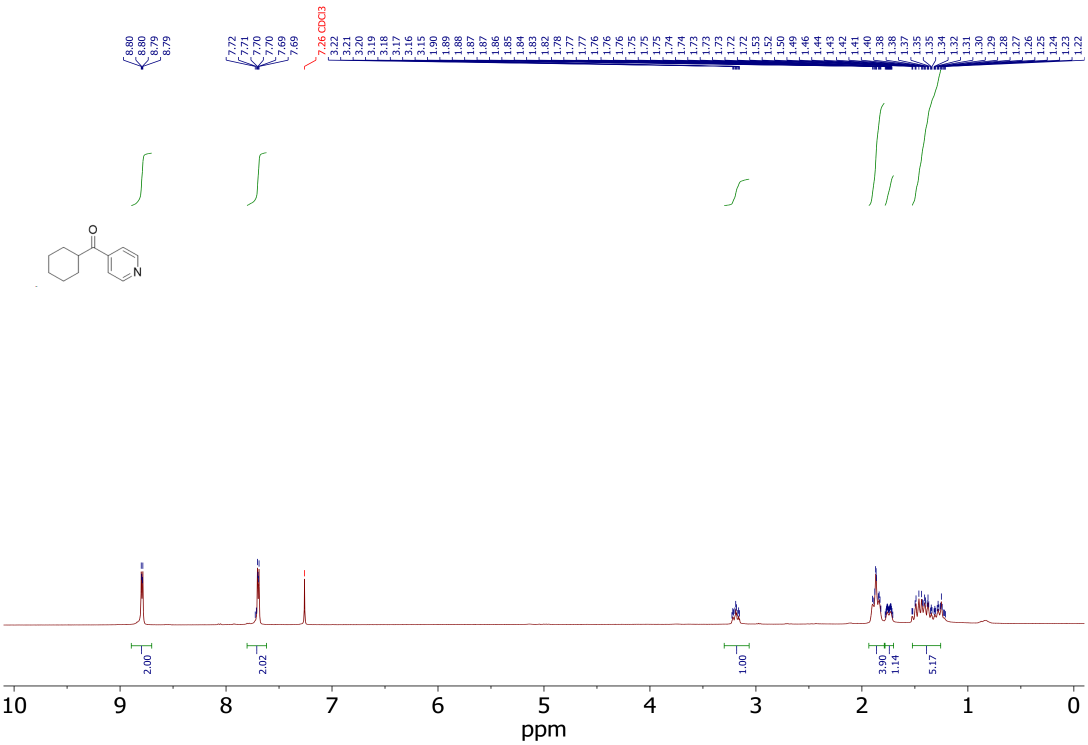

Figure S65. ^{1}H NMR spectrum of **6h** in $CDCl_3$ (400 MHz).

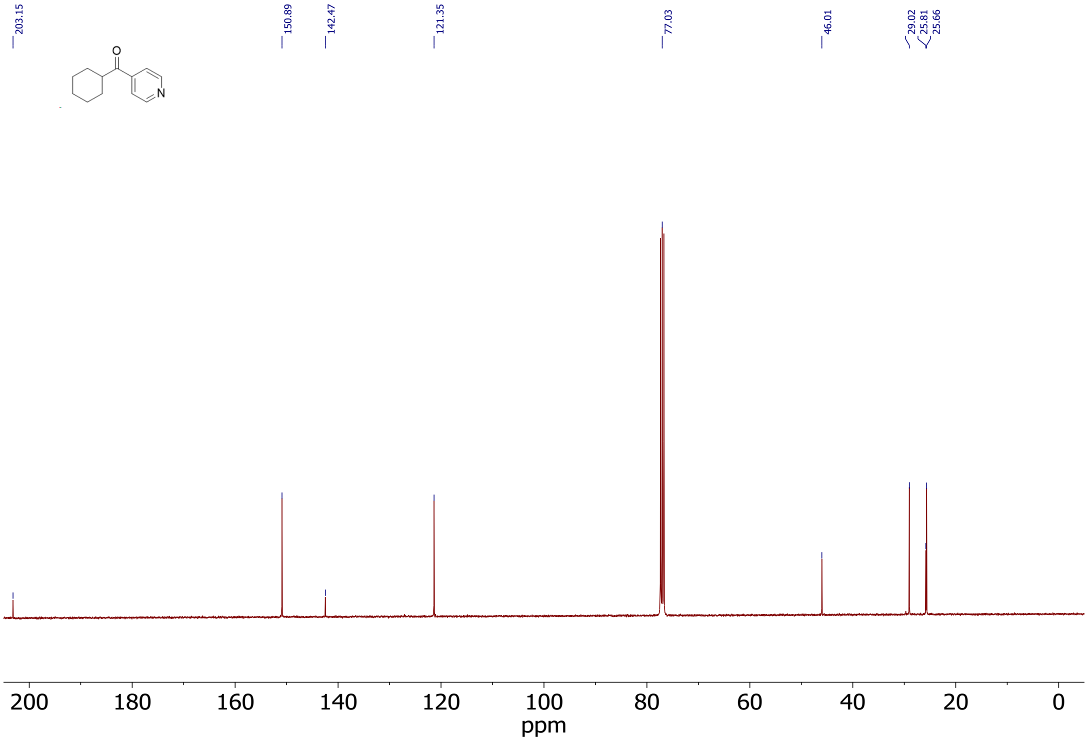

Figure S66. $^{13}C\{^1H\}$ NMR spectrum of **6h** in $CDCl_3$ (101 MHz).

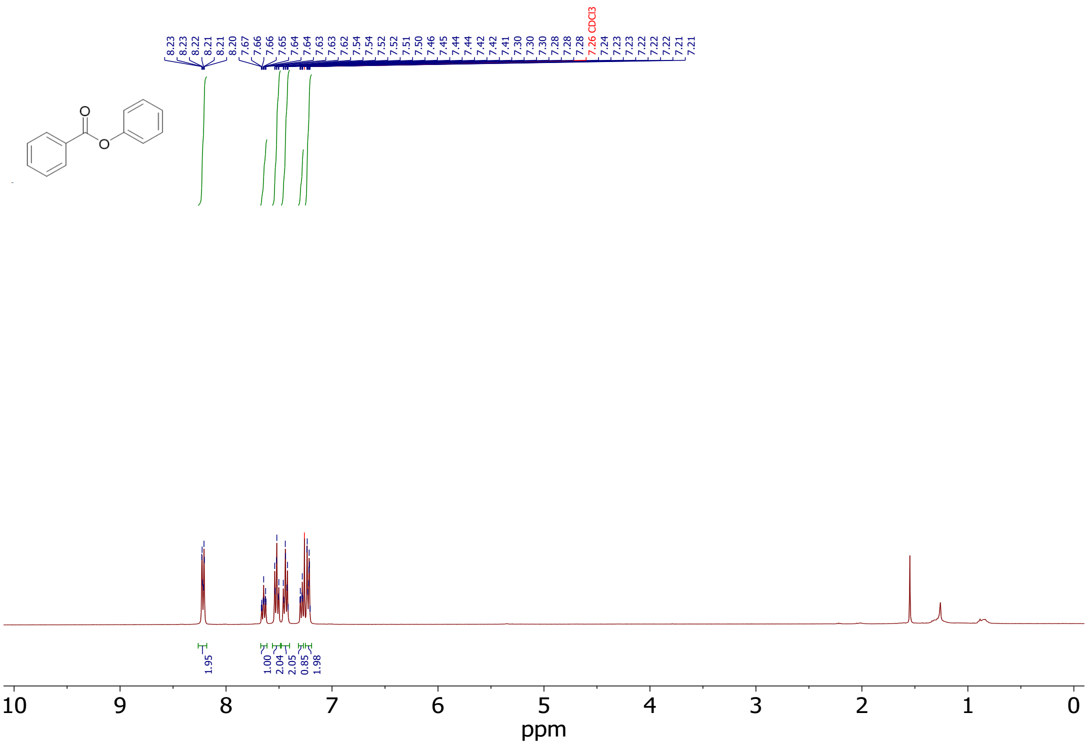

Figure S67. ^{1}H NMR spectrum of phenyl benzoate in $CDCl_3$ (400 MHz).

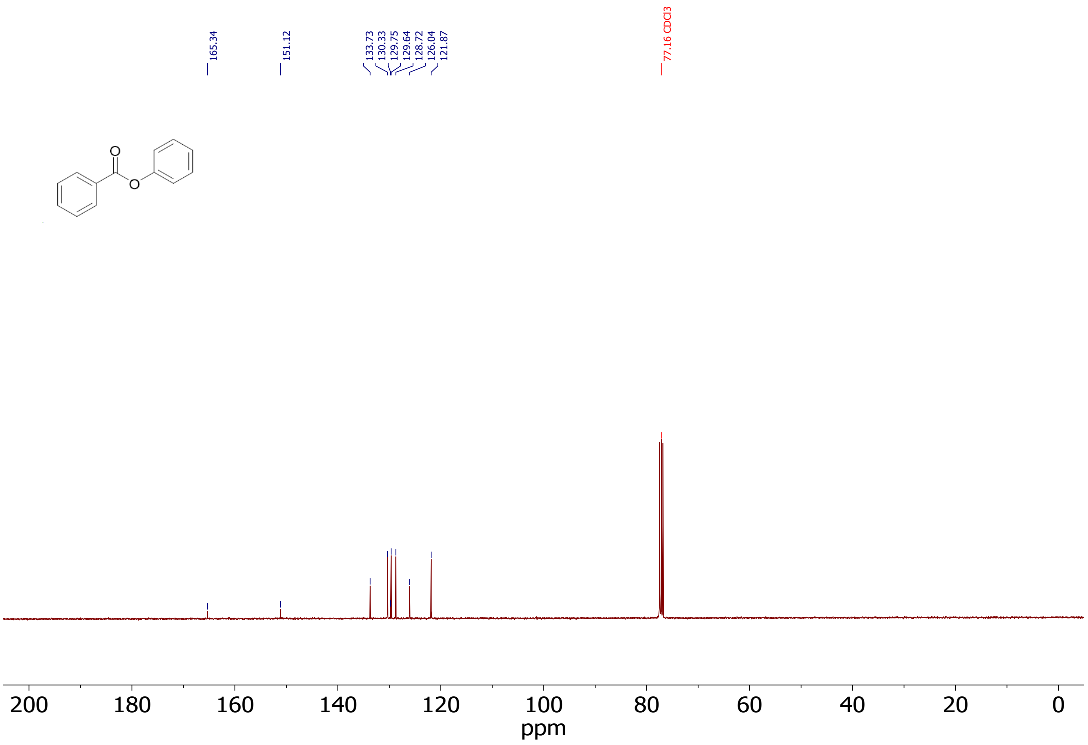

Figure S68. $^{13}C\{^{1}H\}$ NMR spectrum of phenyl benzoate in $CDCl_3$ (101 MHz).

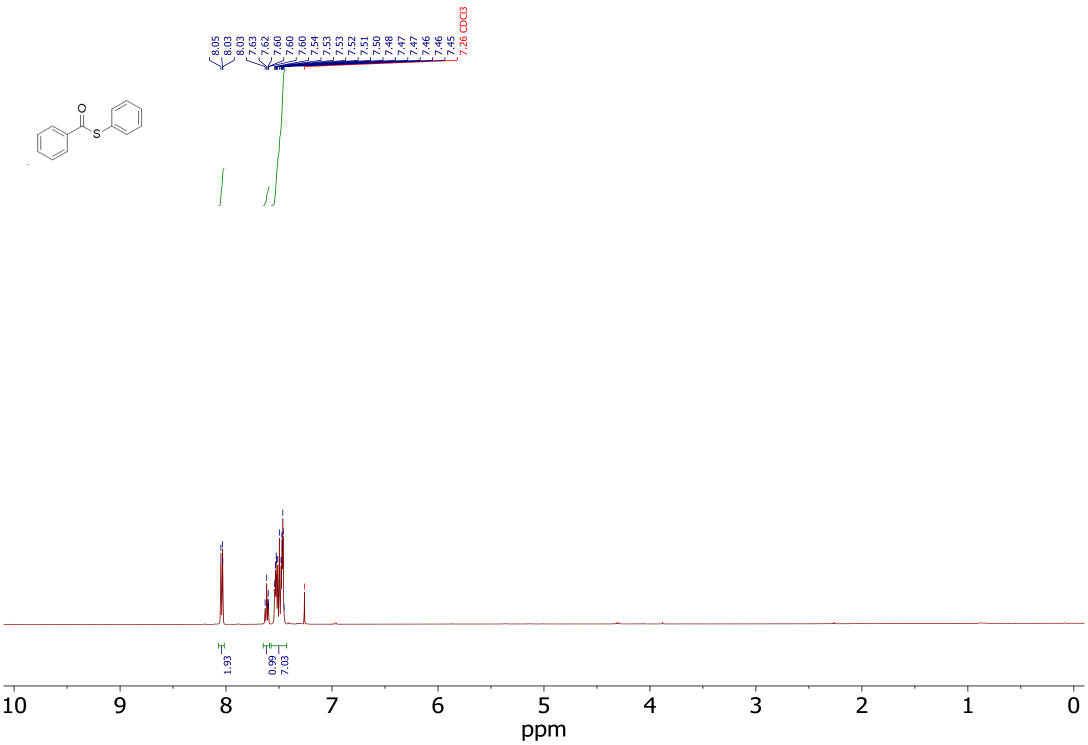

Figure S69. ^{1}H NMR spectrum of S-phenyl benzothioate in $CDCl_3$ (400 MHz).

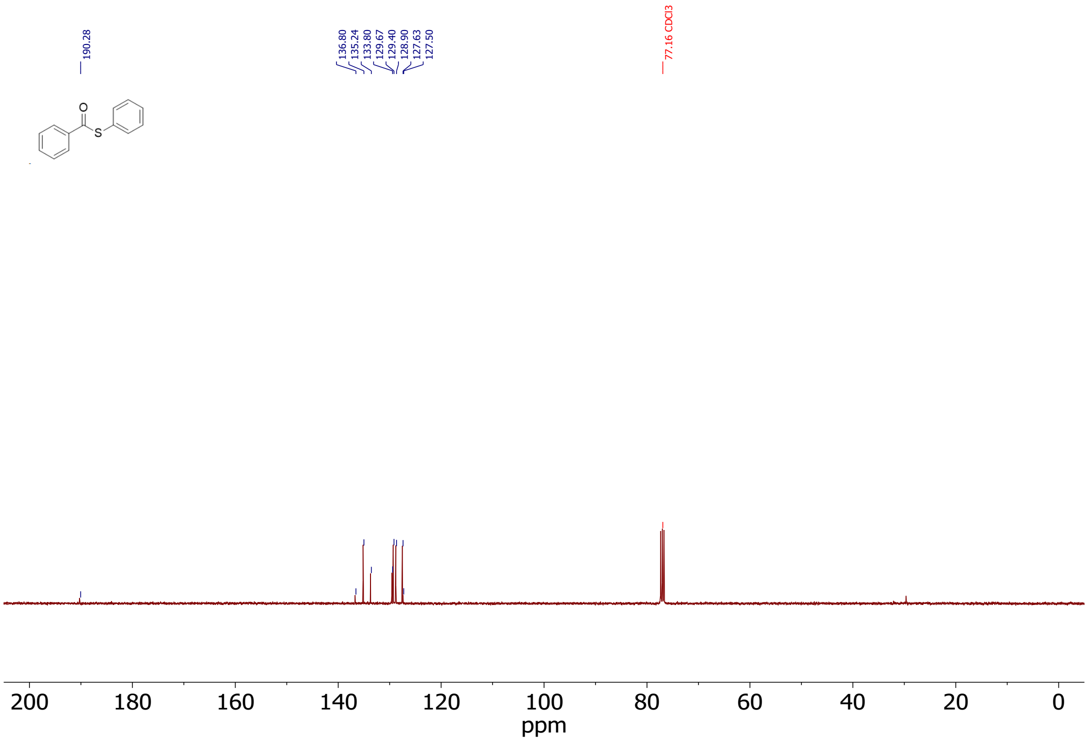

Figure S70. $^{13}C\{^{1}H\}$ NMR spectrum of phenyl benzothioate in $CDCl_3$ (101 MHz).

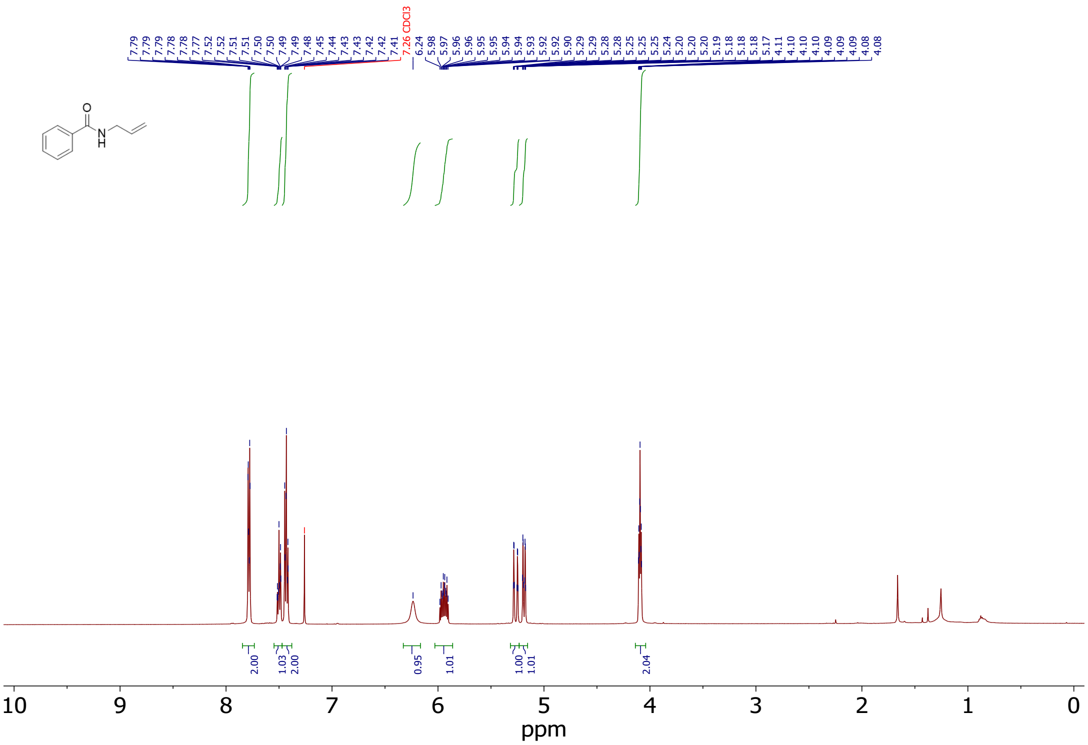

Figure S71. ^{1}H NMR spectrum of N-allylbenzamide in $CDCl_3$ (400 MHz).

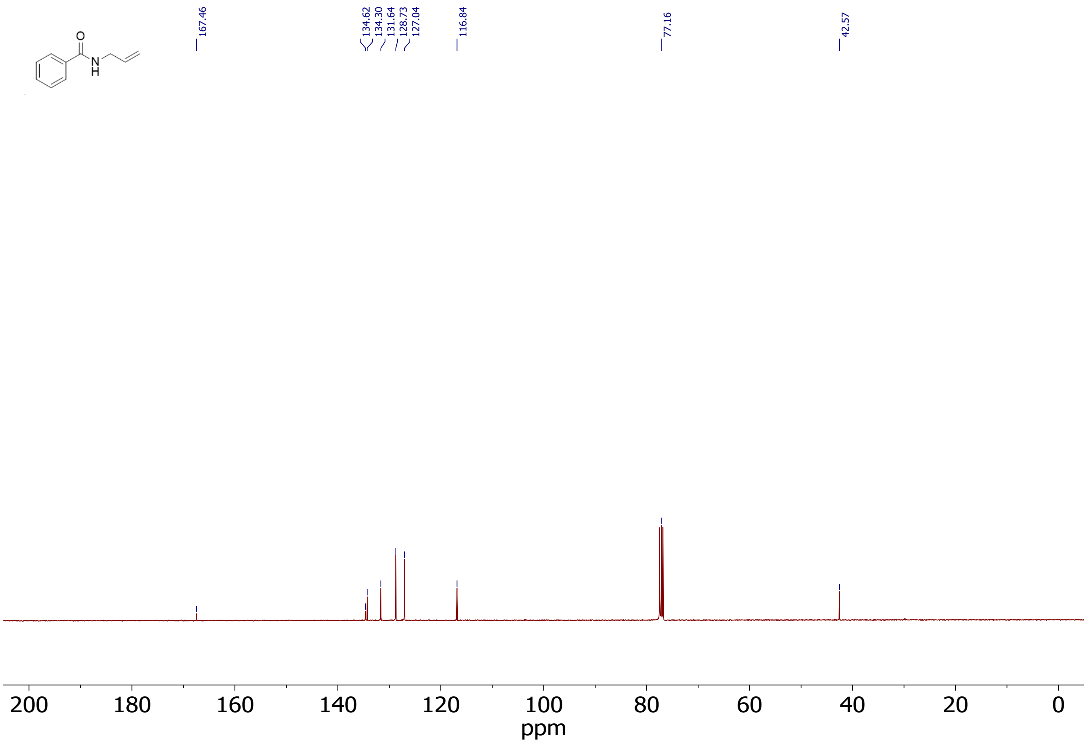

Figure S72. $^{13}C\{^1H\}$ NMR spectrum of N-allylbenzamide in $CDCl_3$ (101 MHz).
